# Healthcare use attributable to COVID-19: a propensity-matched national electronic health records cohort study of 249,390 people in Wales, UK

**DOI:** 10.1186/s12916-023-02897-5

**Published:** 2023-07-19

**Authors:** J Kennedy, M Parker, M Seaborne, M Mhereeg, A Walker, V Walker, S Denaxas, N Kennedy, S. V Katikireddi, S Brophy

**Affiliations:** 1 National Centre for Population Health and Wellbeing Research, Swansea University Medical School, Swansea, Wales UK; 2 Datalab, Nuffield Dept of Primary Care Health Science, Radcliffe Primary Care Building, Oxford, OX2 6GG UK; 3 Bristol Medical School: Population Health Sciences, University of Bristol, Bristol, UK; 4 MRC Integrative Epidemiology Unit, University of Bristol, Bristol, UK; 5 Department of Surgery, University of Pennsylvania Perelman School of Medicine, Philadelphia, PA USA; 6 Institute for Health Informatics, UCL, London, UK; 7 MRC/CSO Social and Public Health Sciences Unit, University of Glasgow, Glasgow, UK

**Keywords:** Long–COVID, COVID-19, SARS-CoV-2, Routine data, Big data, Health data

## Abstract

**Background:**

To determine the extent and nature of changes associated with COVID-19 infection in terms of healthcare utilisation, this study observed healthcare contact 1 to 4 and 5 to 24 weeks following a COVID-19 diagnosis compared to propensity-matched controls.

**Methods:**

Two hundred forty nine thousand three hundred ninety Welsh individuals with a positive reverse transcription–polymerase chain reaction (RT-PCR) test were identified from data from national PCR test results. After elimination criteria, 98,600 positive individuals were matched to test negative and never tested controls using propensity matching. Cohorts were split on test location. Tests could be taken in either the hospital or community. Controls were those who had tested negative in their respective environments. Survival analysis was utilised for first clinical outcomes which are grouped into primary and secondary. Primary outcomes include post-viral-illness and fatigue as an indication of long-COVID. Secondary outcomes include clinical terminology concepts for embolism, respiratory conditions, mental health conditions, fit notes, or hospital attendance. Increased instantaneous risk for positive individuals was quantified using hazard ratios (HR) from Cox regression, while absolute risk (AR) and relative risk were quantified using life table analysis.

**Results:**

Analysis was conducted using all individuals and stratified by test location. Cases are compared to controls from the same test location. Fatigue (HR: 1.77, 95% CI: 1.34–2.25, *p* =  < 0.001) and embolism (HR: 1.50, 95% CI: 1.15–1.97, *p* = 0.003) were more likely to occur in all positive individuals in the first 4 weeks; however, anxiety and depression (HR: 0.83, 95% CI: 0.73–0.95, *p* = 0.007) were less likely. Positive individuals continued to be more at risk of fatigue (HR: 1.47, 95% CI: 1.24–1.75, *p* =  < 0.001) and embolism (HR: 1.51, 95% CI: 1.13–2.02, *p* = 0.005) after 4 weeks. All positive individuals are also at greater risk of post-viral illness (HR: 4.57, 95% CI: 1.77–11.80, *p* = 0.002). Despite statistical association between testing positive and several conditions, life table analysis shows that only a small minority of the study population were affected.

**Conclusions:**

Community COVID-19 disease is associated with increased risks of post-viral-illness, fatigue, embolism, and respiratory conditions. Despite elevated risks, the absolute healthcare burden is low. Subsequently, either very small proportions of people experience adverse outcomes following COVID-19 or they are not presenting to healthcare.

**Supplementary Information:**

The online version contains supplementary material available at 10.1186/s12916-023-02897-5.

## Introduction

Considerable concerns exist about chronic, debilitating, and varied symptoms experienced by people who have had coronavirus disease (COVID-19) caused by severe acute respiratory syndrome coronavirus 2 (SARS-CoV-2) infection [1]. However, the natural history of morbidity and healthcare use after infection and disease remains unclear. While most people who experience COVID-19 recover quickly, an unknown minority experience prolonged symptoms that manifest as a range of post-COVID-19 illnesses [1]. As the pandemic continues, meeting the needs of the increasing numbers of people who have recovered from COVID-19 remains important. Recovery from any severe disease can often be protracted. However, there is increasing evidence that those who were not hospitalised with a COVID-19 infection may have adverse longer-term health consequences such as chronic fatigue and respiratory issues [1].

A greater understanding of the impact and prevalence of symptoms that follow a SARS-CoV-2 infection has been developed in the two years succeeding the start of lockdowns in the UK and worldwide. The sheer number of infections has enabled widespread study in numerous disparate populations. The’UK COVID Symptom Study’ data from August 2020 suggested that around 10% of individuals who had tested positive were still ‘unwell’ after 3 weeks [2]. However, data from a multistate survey in the USA indicated that among those aged 18 to 34 with no chronic comorbidities, 20% had not returned to normal health after 2 to 3 weeks [3]. Observations of those at low risk of mortality from COVID-19 in the UK found that 70% have impairment of one or more organs 4 months after symptom onset [4]. As of July 2022, a statistical bulletin from the Office for National Statistics (ONS) stated that an estimated 1.8 million people had self-reported long-COVID symptoms; 72% of individuals had their day-to-day activities adversely impacted by the symptoms. In this case, self-reported long-COVID is classified as experiencing symptoms that persist 4 weeks post infection [5]. Individuals with more severe COVID-19 were more likely to be disaffected. Follow-up observation of patients admitted with COVID-19 in Michigan US indicated that 15.1% of discharged patients were re-hospitalised within 60 days; nearly half of patients discharged felt emotionally impacted by the current state of their health [6]. In the subsequent 4 to 7 months from disease onset, 63.2% of patients hospitalised with severe to critical infection in Birmingham UK reported experiencing breathlessness, and 36.8% were still in pain [7]. These findings were also corroborated in research from Wuhan China, where fatigue was experienced by 63.0% of individuals 6 months after hospitalisation. In addition, anxiety and depression were also reported by 23.0% [8]. Despite relying on self-reporting, it is evident that many people have been disaffected by symptoms following infection with SARS-CoV-2. Naturally, severity of the initial infection appears to be associated with symptom severity and risk of having persistent symptoms well after the acute phase is over. However, younger low risk individuals are still at risk of long-COVID symptoms [9]. If the incidence of long-COVID reported through healthcare was lower than identified through self-reporting methods (questionnaires), the substantial number of infections will result in a considerable burden of unseen symptoms in the population. However, it is not clear if individuals are presenting to healthcare with these problems, which could be why the numbers present in healthcare are lower when compared to self-reporting [10].

A substantial number of people are experiencing persistent symptoms such as pain, heart palpitations, breathlessness, cognitive impairment, and fatigue [11]. Many symptoms have been reported following COVID-19 [12], with evidence from a symptom tracker in the UK suggesting the existence of six different syndromes. However, consensus on what clusters of sequelae exist is not available. The highly varied nature of symptoms and experiences reported by patients has made standardised diagnosis difficult. In particular, accurate clinical coding of long-COVID has been lacking, thereby impeding research efforts [13]. However, age, self-reported health status before the onset of symptoms, self-reported pre-existing comorbidities, and the number of symptoms during the infection were found to significantly predict the number of symptoms patients with long-COVID may experience at follow-up [14]. The most common causes for GP attendance 4 weeks after infection were joint pain (2.5%), anxiety (1.2%), and prescription of non-steroidal anti-inflammatory drugs (1.2%); these were identified using routine medical record data [15].

Health systems internationally have been under extreme pressure due to the COVID-19 pandemic. Many countries have faced large demands for healthcare, resulting in elective care being postponed with many patients foregoing or delaying necessary treatment. These stresses have resulted in large waiting lists within the UK. The large numbers of SARS-CoV-2 infections could lead to a further demand on healthcare because of long-COVID. At present, there is limited data [15] available to inform health systems about the scale of demand that might be expected and what services might be sought. However, establishing the extent to which these conditions are attributable to COVID-19 or reflect disease burden among the general population can be difficult. Misclassification may also occur because of the general misunderstanding of long-term consequences of COVID-19 and the likelihood that clinicians may attribute unrelated illness, or escalation of existing symptoms, to COVID-19.

Research to establish the natural history of the COVID-19 disease over the medium- and long- term can inform understanding of the long-term effect of COVID-19, and potentially inform expectations about future health system demands. This study therefore aims to develop an understanding of the burden on the healthcare system attributable to COVID-19, quantify the length of time of excess resource use, and categorise the different diagnostic codes that underpin any excess healthcare use.

## Methods

### Study population (28 February 2020 to 26 August 2021)

This cohort study utilised the Secure Anonymised Information Linkage (SAIL) Databank in Wales [16], which includes nation-wide electronic health records from primary and secondary care. The SAIL databank is a data repository which allows person-based data linkage across datasets. This databank includes Welsh GP data and hospital in- and out-patient records, as well as mortality data collected by the Office of National Statistics (ONS). SAIL holds over a billion anonymised records and has Welsh population coverage for 100% of hospital data and 86% of GPs. It employs a split-file approach to ensure anonymisation and overcome issues of confidentiality and disclosure in health-related data warehousing. SAIL has been benchmarked against 17 other data research platforms from 10 European countries. SAIL was recognised for numerous features including online guidance, informational resources, and dedicated public engagement expertise, as well as being recognised for the method and speed in which COVID-19 data was managed [17]. Demographic data are sent to a partner organisation, NHS Wales Informatics Service, where identifiable information is removed; clinical data are sent directly to the SAIL Databank and an individual is assigned an encrypted anonymised linking field (ALF). The ALF is used to link anonymised individuals across datasets, facilitating longitudinal analysis of an individual’s journey through multiple health, education, and social datasets [16].

**Fig. 1:**
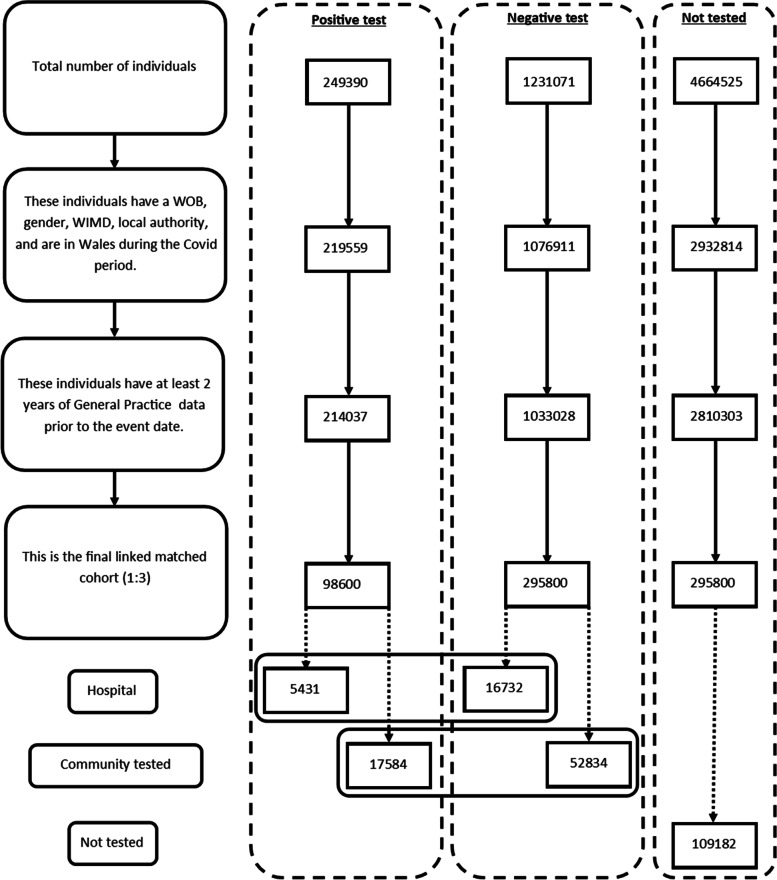
Flow diagram of participants. WOB, week of birth; WIMD, Welsh Index of Multiple Deprivation

The data linked in this study (Fig. 1) were as follows: Welsh Demographic Service to identify all patients registered with a GP practice and identify when people move in and out of Wales, primary care GP dataset to identify healthcare contacts in general practice, data collected by GPs are captured via Read Codes version 2 (5-character alphanumeric codes related to diagnosis, medication and process of care codes) [18], the hospital in-patient and out-patient data collected in the Patient Episode Database for Wales, which contains clinical information regarding patients’ hospital admissions, discharges, diagnoses, and operations using the International Classification of Diseases 10th revision (ICD-10) clinical classification system. The ONS Mortality dataset contains demographic data, place of death, underlying cause of death (also ICD-10), and test results from the laboratory management information system to identify individuals who have had a laboratory COVID-19 test as well as the test result.

### Identifying SARS-CoV-2

The study identified all people in Wales registered with a Welsh GP and stratified them as having had a SARS-CoV-2 positive test result, a negative result, or no SARS-CoV-2 test between 28 February 2020 and 26 August 2021. Exposure to SARS-CoV-2 infection is defined as starting from the first date of a positive reverse transcription–polymerase chain reaction (RT-PCR) test result. In addition, given that the consequences of COVID-19 may differ depending on the severity of the initial disease, tests were also stratified by test site location. Tests were classified to have occurred at community sites, hospital sites, or unknown sites (see Additional file 1: Table S1 for what constitutes a ‘community’ and ‘hospital’ testing site).

### Study design

Individuals could be enrolled into the study between 28 February 2020 and 26 August 2021. An individual could be allocated to one of three groups at any given time: positive case, negative control, or never tested control. The index date is the following: date of the first positive test, date of the first negative test, or an allocated pseudo date for these three groups respectively. It indicates the start of the follow-up period for that individual. Individuals were followed up for 6 months from their index date at which point they would be censored. Individuals were also censored before the end of the follow-up if the end of study date was reached (26 August2021), they move out of Wales, or they died. Individuals were able to move groups upon change of test status. If a negative control received a positive test, they would be censored from the control group and start a new follow-up period in the case group. Similarly, if a never tested control received a RT-PCR, their current follow-up would be censored, and they would start a new follow-up as part of the negative control or positive case group depending on the result of the test.

**Fig. 2:**
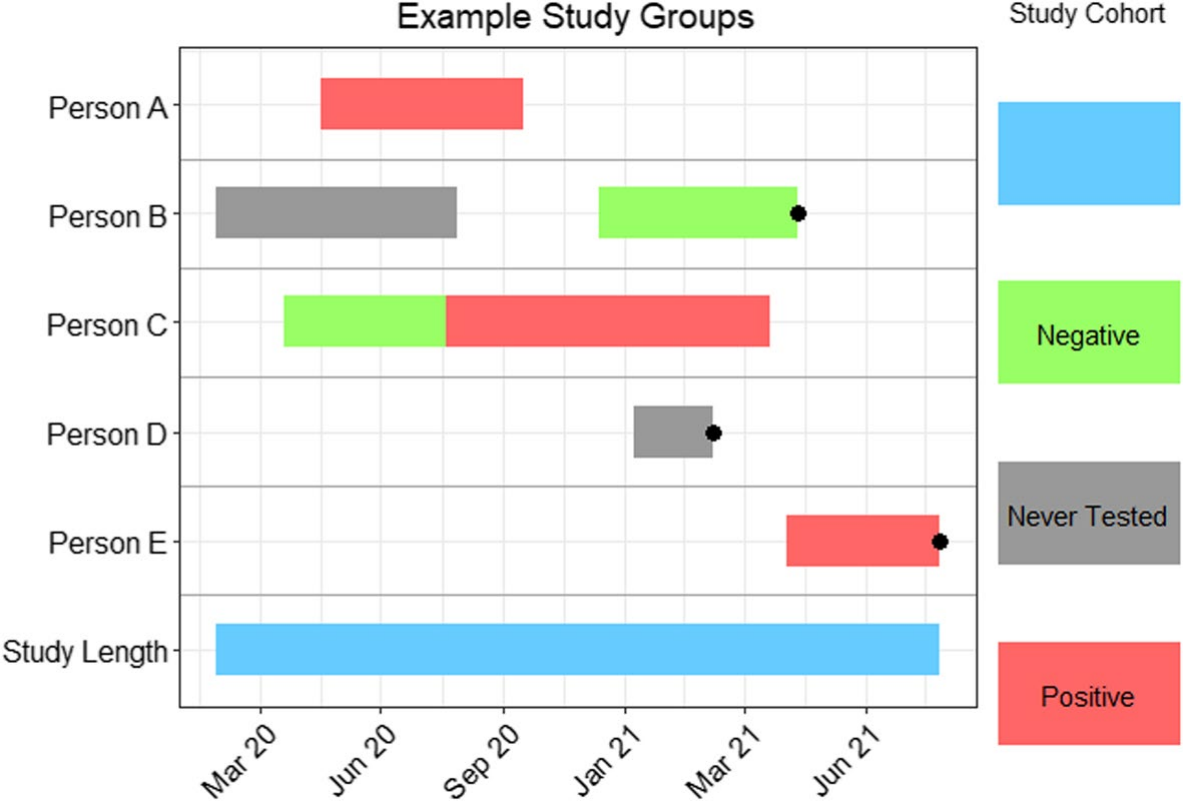
Timeline plot showing mock data to demonstrate how individuals can move between case and control groups. Black bullet points indicate premature censoring

See Fig. 2 which displays study group movement for fabricated individuals. The above plot shows how individuals may move and be censored between the different study groups. Person A tests positive with no other tests on record in May 2020; they go through the 6 month follow-up, and they are censored in October 2020. Person B is allocated to the never tested cohort for which they go through the whole follow-up and are censored in August 2020. They then test negative in December 2020 and proceed to have a follow-up; due to one of the censorship reasons stated, they end the follow-up early, either through death or moving out of Wales. Person C tests negative in early 2020 but part way through the follow-up they test positive and complete a new follow-up period in the positive group.

A combination of propensity and exact matching methods were utilised to adjust for confounders in the data, allowing the investigation of primary and secondary healthcare use after a positive RT-PCR test result. Examples of the variables utilised for propensity include number of previous COVID-19 tests and examples of exact matching includes variables such as age and gender [19]. A positive case was matched with three controls from their respective test environment and three never tested controls. Table 1 shows how each variable was used in the matching process. In this study, variables used for matching were the following: Welsh Index of Multiple Deprivation Quintile (WIMD), comorbidities using the Charlson Comorbidity Index (CCI), number of people in the household, and number of previous SARS-CoV-2 tests. In addition to the propensity score, the cases and controls were exact matched on gender, local authority area, week of birth (± 1 year from date), and location of the test (community/hospital/other). Propensity scores were used to adjust for confounders in testing positive for COVID. Propensity matching was selected for simplicity of interpretation as it provides one score for matching as opposed to controlling for multiple confounders in a regression analysis. The sample size was of sufficient size to enable high match rates.

**Table 1:** Information on how each variable was used in the matching process

Variable	Controlled by
Deprivation of local area	Included in propensity-matched score
Co-morbidities	Included in propensity-matched score
Number in household	Included in propensity-matched score
Number of previous COVID tests	Included in propensity-matched score
Gender	Exact matching
Region	Exact matching
Age	Exact matching
Testing location	Exact matching

### Data cleaning

Data were checked for patterns of missingness and implausible values for all analytical variables investigated. A record of reasons for exclusion from analysis was maintained. Individuals with no recorded test location (excluding the never tested population) were excluded from the analysis.

### Study outcomes

The primary outcome was to determine whether testing positive for SARS-CoV-2 results in different use of primary and secondary care in the first 6 months following the test, compared to those who had currently not tested positive. The primary objective is to identify conditions presenting in healthcare that could signify long-COVID such as fatigue or post-viral illness. However, a further aim is to identify other conditions that may be exacerbated by a positive SARS-CoV-2 infection and result in presentation to a healthcare setting.

The never tested population was compared with the negative group to understand bias in who attends for testing as those who have never been tested are also unlikely to attend for healthcare for other conditions. The negative test population was also set as the reference group when the data are stratified by test location (community or hospital). Follow-up starts on the day of being classified as exposed (or the date of being matched for controls). Follow-up ends when an individual experiences the outcome of interest or has been censored, due to study end date, moving out of Wales, or death.

### Statistical analysis

Descriptive statistics were undertaken on the negative test, positive test, and never tested populations to assess the adequacy of the propensity matching. The frequency of deaths and care visits (primary and secondary) were tracked each month and adjusted for the population size, accounting for individuals who had been censored. General groups of codes used to define the clinical outcomes of this study can be observed in Additional file 2: Table S2 (full list in Additional file 3: Table S3). Sick notes only refer to those issued by the GP and not self-certified notes, whether the codes originated in primary or secondary and additional notes are also included in Additional file 2: Table S2. Outcomes include death, first secondary care visit, diabetes, embolism, fatigue, mental and behavioural disorders, respiratory conditions, post viral illness, and sick notes. Alternatively, an individual could be censored. Reasons for censorship include death (from any cause), end of follow-up period (28 days or 168 days), end of study period (01 August 2021), and changing COVID-19 test status through a confirmed RT-PCR test or leaving Wales.

Secondary analyses examined the following: (a) healthcare use and (b) the length of time by which the excess risk associated with SARS-CoV-2 infection and COVID-19 disease has ended.

Survival analysis was utilised to examine the time between an individual’s first RT-PCR and the first occurrence of an outcome or endpoint. The time between the index date and the endpoint was calculated for each different outcome independently. Age has been calculated using the week of birth and the date of first test or index date divided by 365.25 to provide the age in years.

Cox proportional hazard models were used to produce hazard ratios (HR) to quantify the likelihood of the first instantaneous occurrence of an outcome within (a) 0–28 days (1 month) and (b) 29–168 days (5 months) following a RT-PCR. The never tested population was compared with the negative group to understand bias in who attends for testing as those who are never tested are also unlikely to attend for healthcare for other conditions. The negative test population was also set as the reference group when the data are stratified by test location (community or hospital). Follow-up starts on the day of being classified as exposed (or the date of being matched for controls). Follow-up ends when an individual experiences the outcome of interest or has been censored, due to study end date, moving out of Wales, or death.

Individual models were run for each outcome, time frame (1 to 4 weeks and 5 to 24 weeks), and location (combined location, hospital only, and community only). Dataset conditions were dependent on the time frame being studied: (a) 1 to 4 weeks: the end of the follow-up period was 28 days from the index date and (b) 5 to 24 weeks: the follow-up period was between 29 and 168 days from the index date. If an RT-PCR positive individual died within the first 28 days, all their propensity-matched partners were also removed from the analysis. Life table analysis utilises the full follow-up period between day 0 and 168 from the first test or index date.

### Life table analysis

Risk ratios (RR) showing the relative risk of an outcome every 4 weeks compared to a reference group were calculated through life table analysis. The analysis creates a ratio of absolute risk (AR) for each outcome adjusting the population size as individuals are censored. The reference groups for those tested in the community and hospital settings are negative tests in their respective environments. The reference group for the never tested population were negative tests in the community only as this enabled the exploration of the bias in healthcare use for those who have not been tested.

### Software

**Fig. 3:**
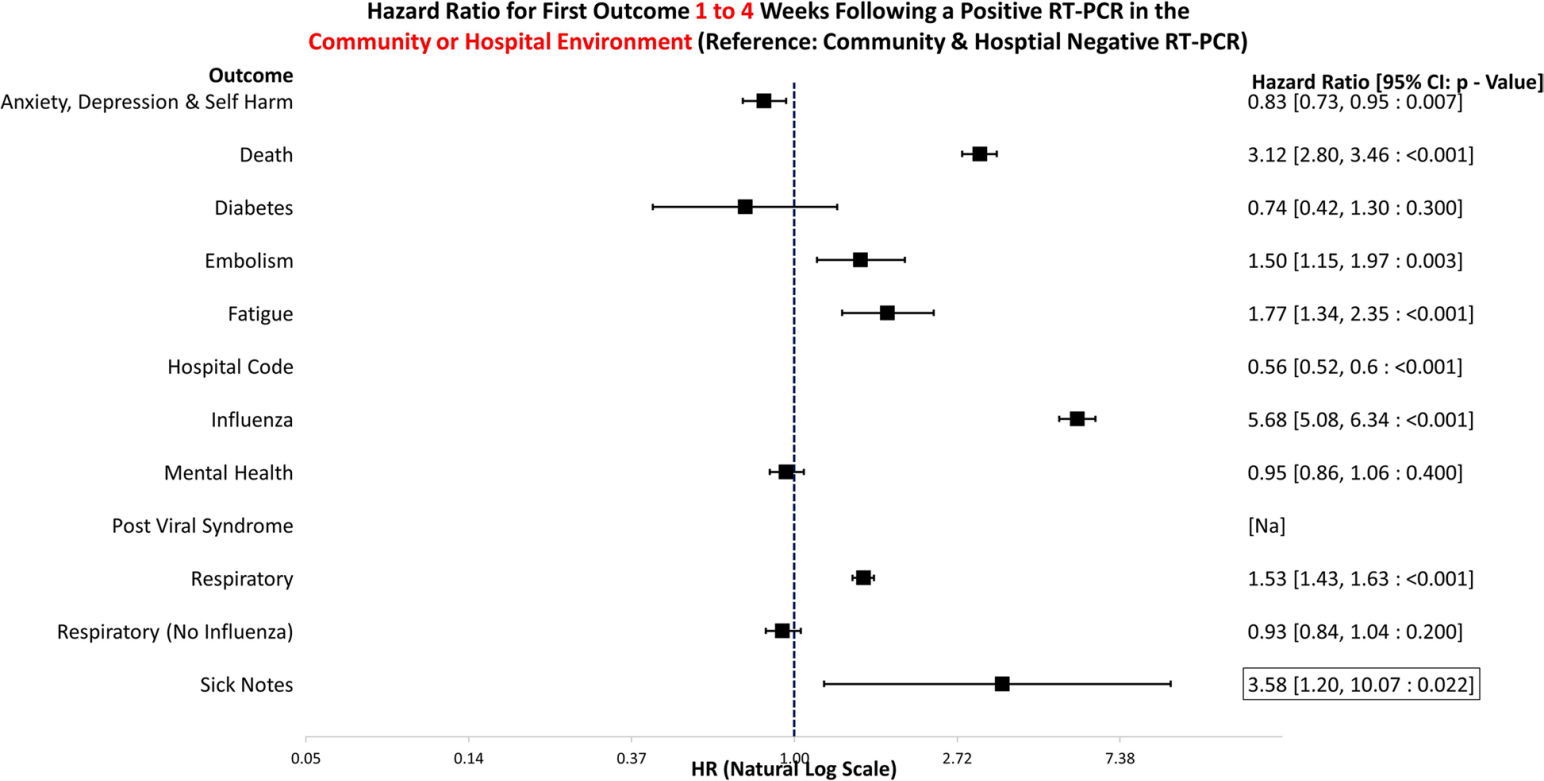
Hazard ratio for risk of study outcomes 1 to 4 weeks following a RT-PCR in both the hospital and community test environment. HR, hazard ratio; CI, confidence interval

**Fig. 4:**
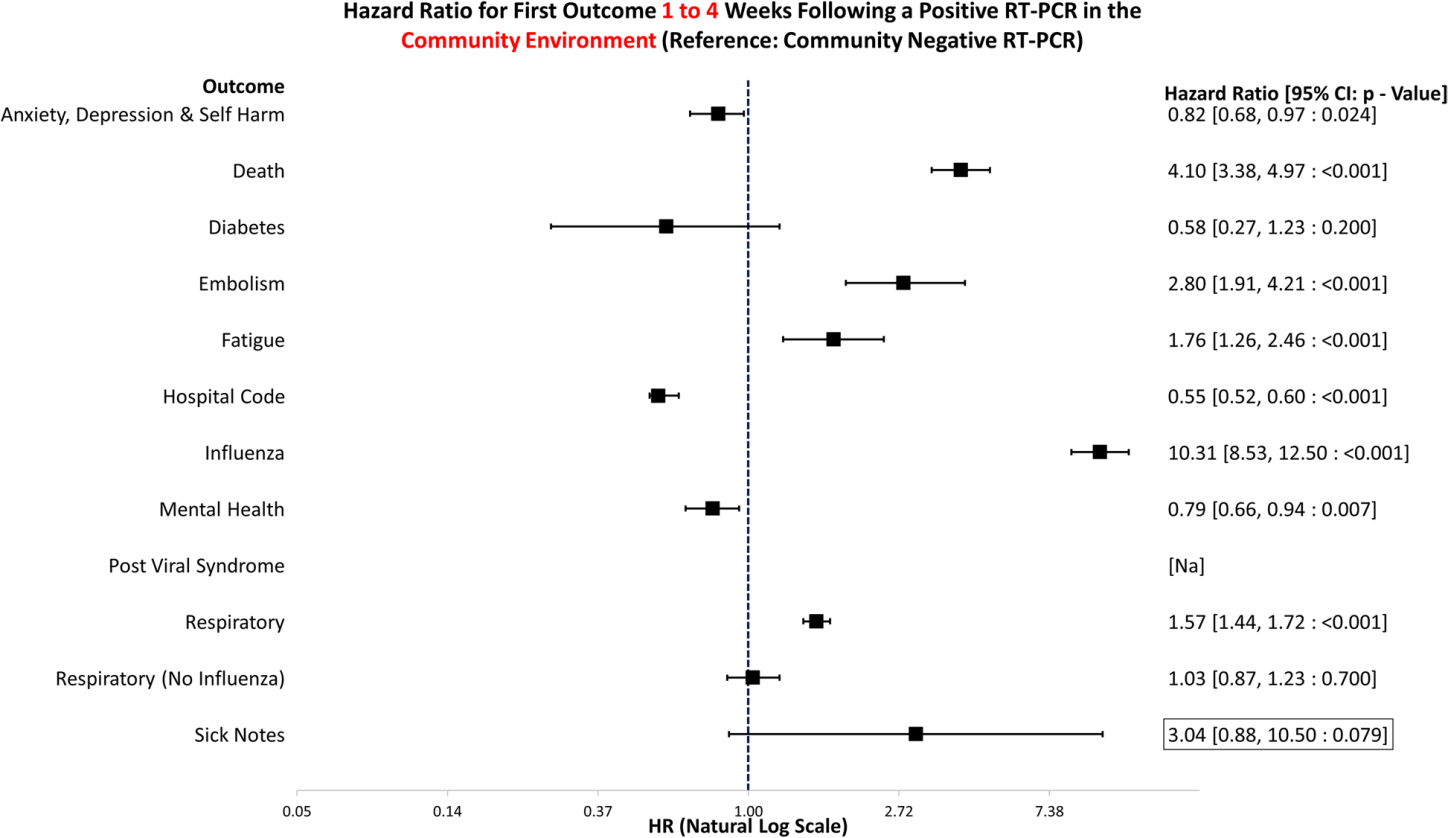
Hazard ratio for risk of study outcomes 1 to 4 weeks following a RT-PCR in the community test environment only. HR, hazard ratio; CI, confidence interval

**Fig. 5:**
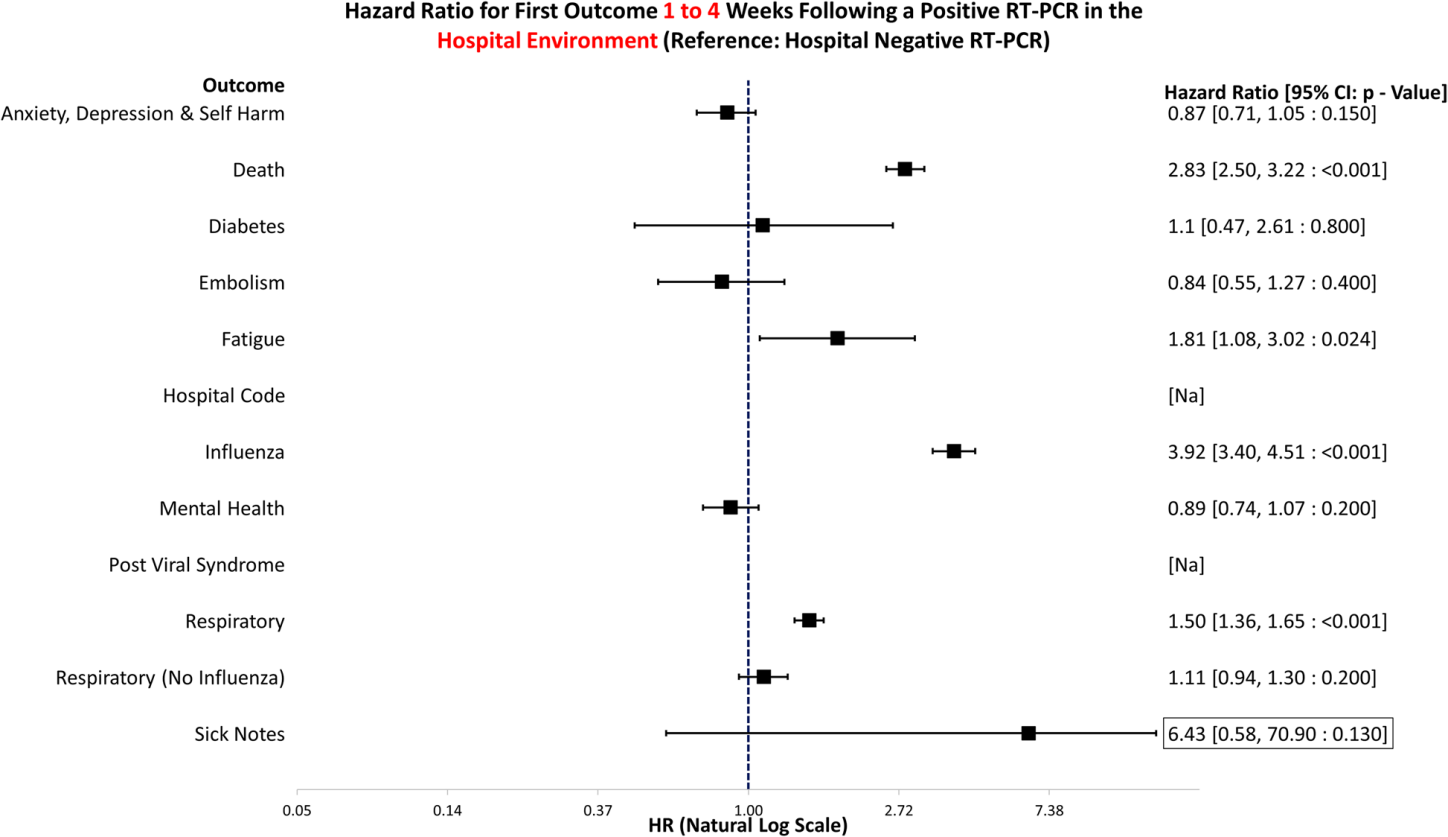
Hazard ratio for risk of study outcomes 1 to 4 weeks following a RT-PCR in the hospital test environment only. HR, hazard ratio; CI, confidence interval

**Fig. 6:**
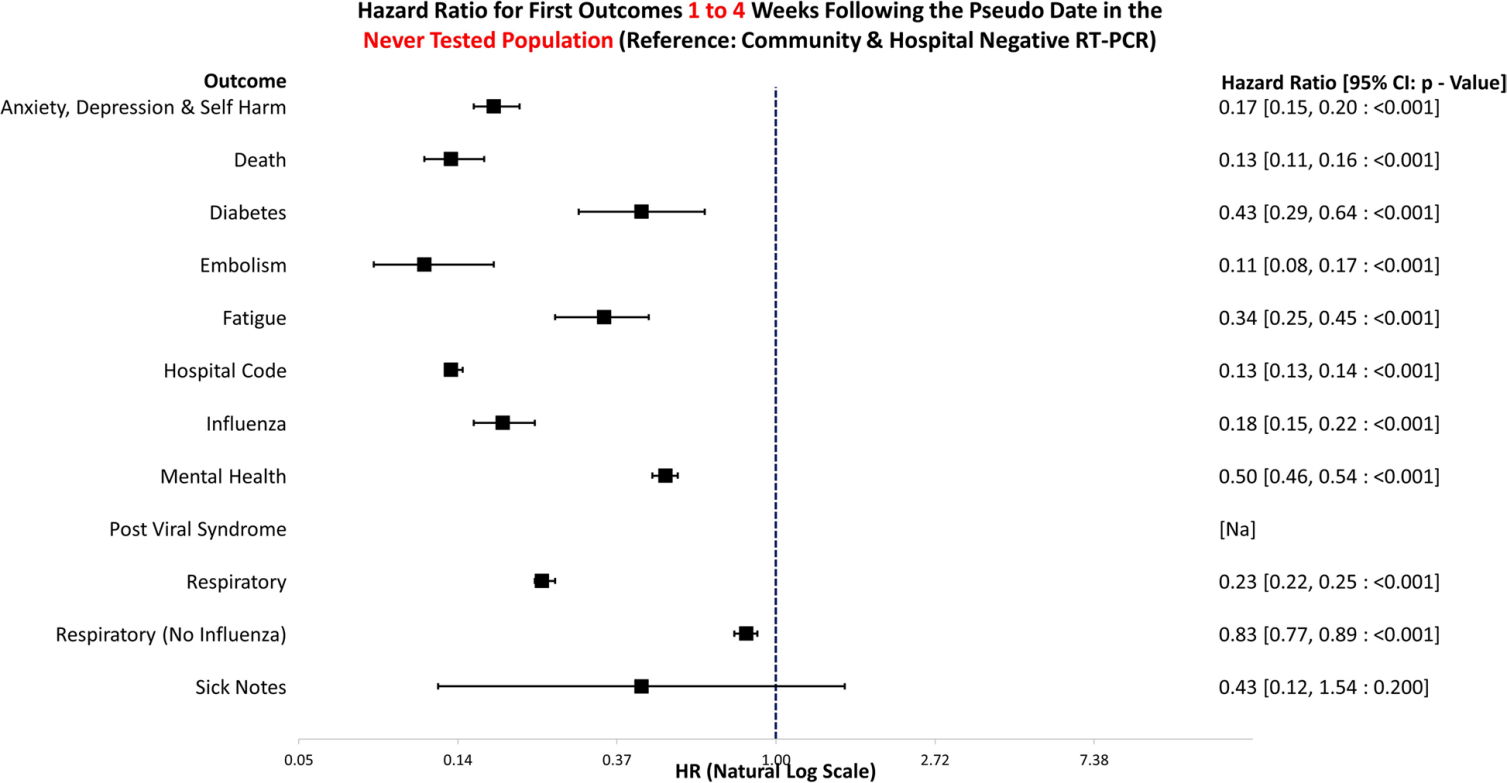
Hazard ratio for risk of study outcomes 1 to 4 weeks following a pseudo date in the never tested population. HR, hazard ratio; CI, confidence interval

**Fig. 7:**
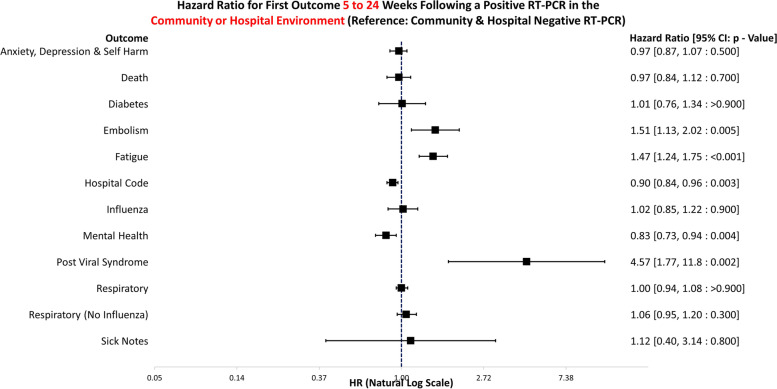
Hazard ratio for risk of study outcomes 5 to 24 weeks following a RT-PCR in both the hospital and community test environment. HR, hazard ratio; CI, confidence interval

**Fig. 8:**
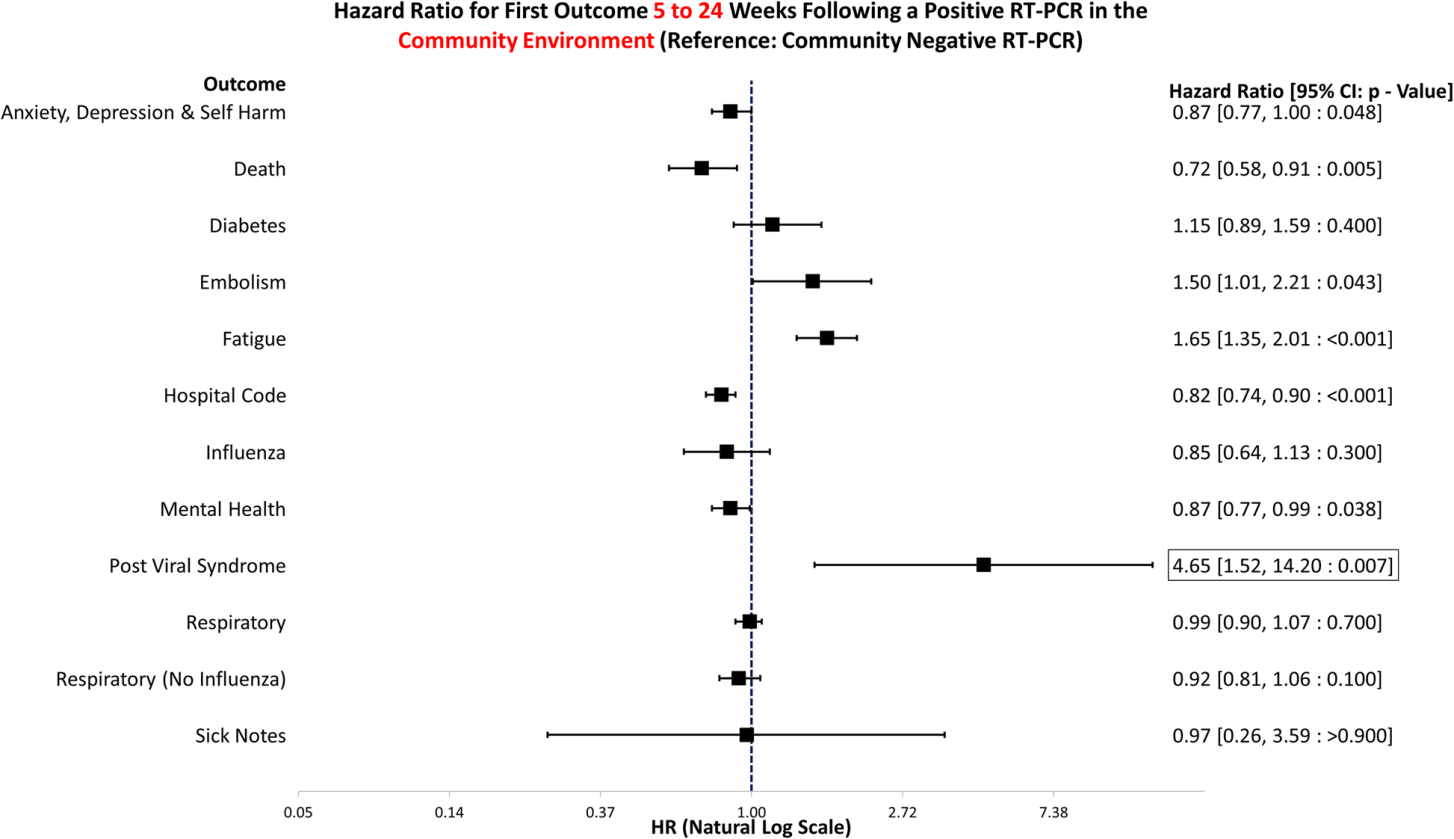
Hazard ratio for risk of study outcomes 5 to 24 weeks following a RT-PCR in the community test environment only. HR, hazard ratio; CI, confidence interval

**Fig. 9:**
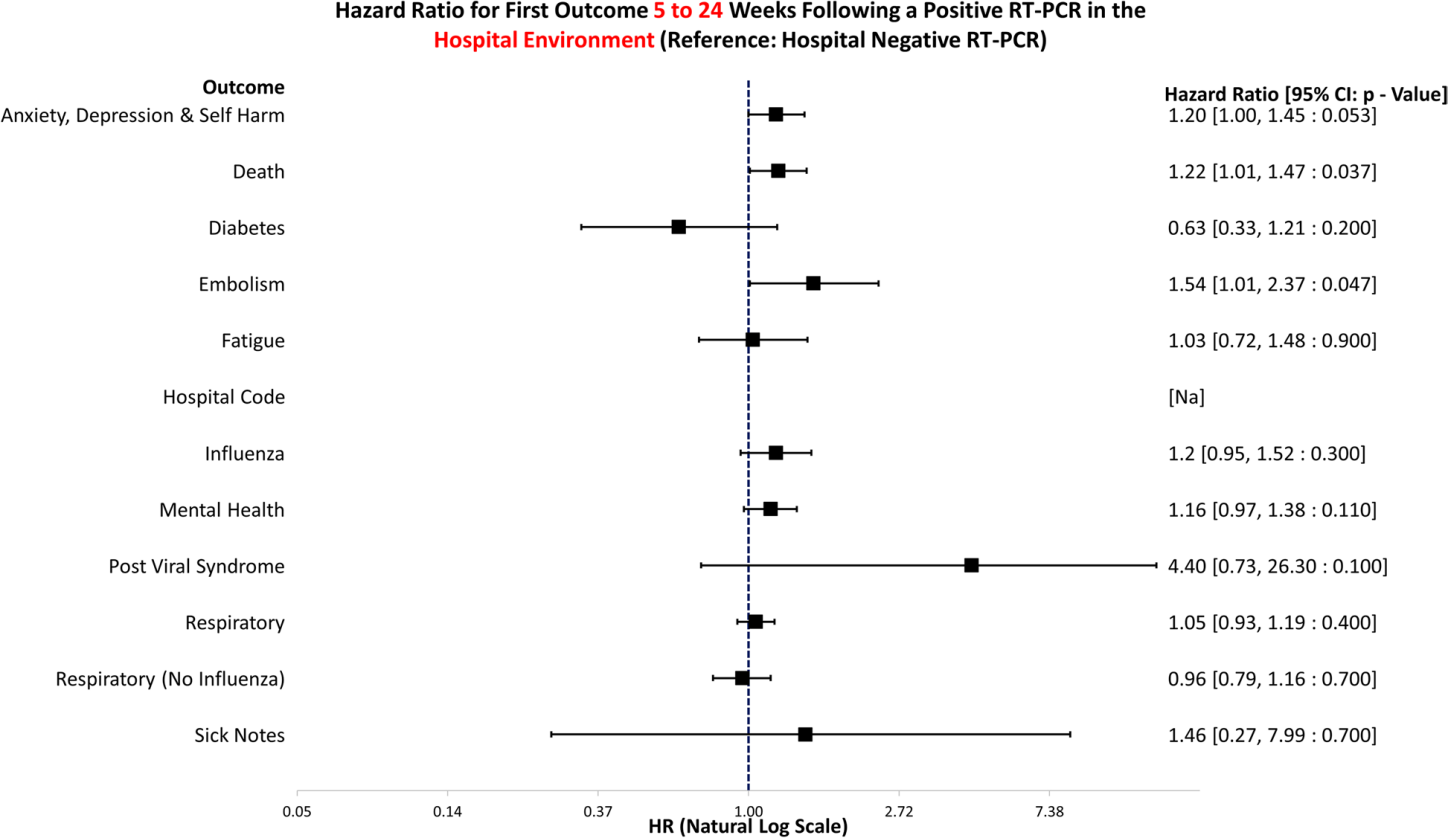
Hazard ratio for risk of study outcomes 5 to 24 weeks following a RT-PCR in the hospital test environment only. HR, hazard ratio; CI, confidence interval

**Fig. 10:**
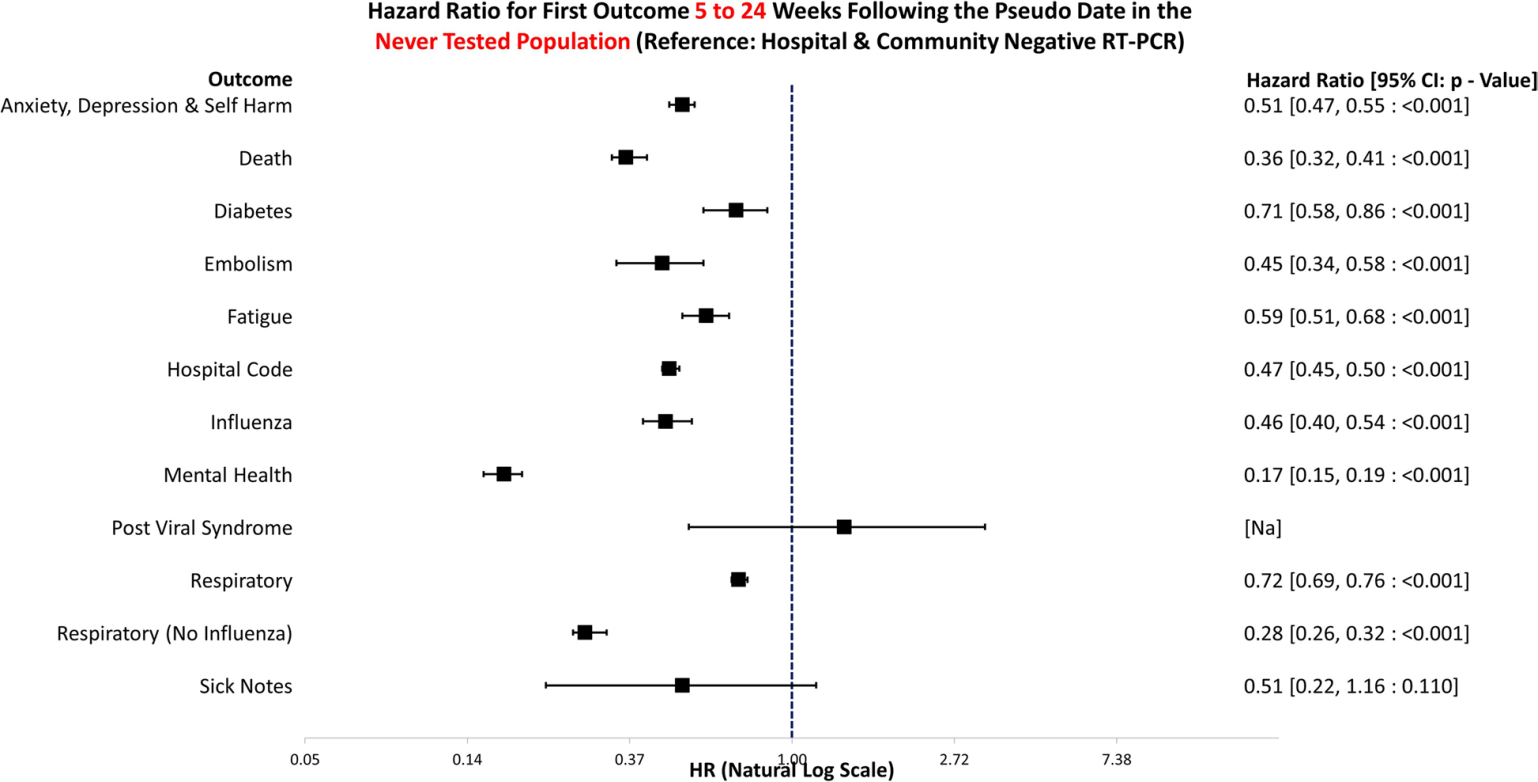
Hazard ratio for risk of study outcomes 5 to 24 weeks following a pseudo date in the never tested population. HR, hazard ratio; CI, confidence interval

The data handling and preparation for survival analysis, descriptive statistics, and life table analysis were performed in an SQL database (SAIL) using Eclipse [20] and tabulated in Microsoft Excel for database extraction. Final data preparations specific to survival analysis were performed in RStudio 2021.09.0 such as setting reference groups for the Cox proportional hazard models [21]. Survival analysis was performed in R studio utilising the packages ‘Survminer’ [22] and ‘Survival’ [19]. ‘Love Plots’ (Additional file 4: Fig. S1) were created in R using the package ‘cobalt’ [23]. Risk ratio and confidence intervals (CI) calculations were performed in Microsoft Excel (Version 2201), and hazard ratio plots (Figs. 3, 4, 5, 6, 7, 8, 9, and 10) were also manually constructed in Microsoft Excel.

### Ethical approval

Data held in the SAIL databank are anonymised, and therefore no NHS ethical approval is required. All data contained in SAIL has the permission from the relevant Caldicott Guardian or Data Protection Officer, and SAIL-related projects are required to obtain Information Governance Review Panel (IGRP) approval. The IGRP approval number for this study is 1259.

## Results

### Demographic of case controls

Demographic information can be observed in Table 2. There were 249,390 individuals who had a positive SARS-CoV-2 test between 28 February 2020 and 26 August 2021. Following the application of inclusion criteria, propensity matching with controls reduced this to 98,600 individuals, thus removing 60% of the data. The dataset was then further restricted by removing all matches for whom their test location was matched as missing. When stratified by COVID-19 testing, these numbers were 5431 tested in hospital, 17,584 tested in community, and 75,585 with no known location.

**Table 2:** Demographic profile of propensity-matched infected and non-infected individuals analysed

	Case (+ ve test)		Control (- ve test)		Control
	**Community**	**Hospital**	**Community**	**Hospital**	**Never tested**
Total, *n* (%)	17,584 (24.97)	5431 (24.50)	52,834 (75.03)	16,732 (75.50)	109,182 (100.00)
Males, *n* (%)	8,222 (46.76)	2770 (51.00)	24,856 (47.05)	8361 (49.97)	54,435 (49.86)
WIMD score 1 or 2, *n* (%)	3945 (22.44)	1233 (22.70)	11,862 (22.45)	3441 (20.56)	21,694 (19.87)
Mean age (years (Std.D))	42.56 (23.47)	51.90 (25.83)	42.74 (23.56)	50.66 (22.92)	43.17 (23.95)
Mean Charlson Index (Std.D)	0.60 (1.00)	1.13 (1.53)	0.55 (1.01)	1.03 (1.47)	0.53 (0.89)

*WIMD* Welsh Index of Multiple Deprivation, *Std.D* Standard deviation, + *ve test* Positive test, *-ve test* Negative test

Three matched cohorts are used in this study; COVID-19 test positive (case), COVID-19 test negative (control), and never tested (control). 23,015 (hospital and community tested) and 69,566 (hospital and community tested) individuals were identified to have had a positive and negative test respectively. Additional file 4: Fig. S1 shows ‘Love Plots’ for the standardised mean distribution before and after the propensity matching had arisen. Censorship patterns were checked and were similar across the cohorts.

### Outcomes 1 to 4 weeks following a positive RT-PCR

Underlying data for Figs. 3, 4, 5, 6, 7, 8, 9, and 10 can be found in Additional file 5: Table S4. Additional file 6: Fig. S2 and Additional file 7: Fig. S3 show survival curves for the full 6-month follow-up for the Embolism and Fatigue outcomes. Further curves have not been shown due to the infrequency of the outcomes seen in this study.

### All locations

Figure 3 illustrates the hazard ratios for altered risk of outcomes 1 to 4 weeks following a positive RT-PCR in either the community or hospital environment. The reference group is negative test in the both the hospital and community environments. In the first 4 weeks, COVID-19-positive individuals are at a significantly greater risk of death (HR: 3.12, 95% CI: 2.80–3.46, *p* =  < 0.001), embolism (HR: 1.50, 95% CI: 1.15–1.97, *p* = 0.003), fatigue (HR: 1.77, 95% CI: 1.34–2.35, *p* =  < 0.001), influenza codes (HR: 5.68, 95% CI: 5.08–6.34, *p* =  < 0.001), respiratory conditions (HR: 1.53, 95% CI: 1.43–1.63, *p* =  < 0.001), and issuing of sick notes (HR: 3.58, 95% CI: 1.20–10.07, *p* = 0.022). Conversely, they were at significantly lower risk from anxiety, depression, and self-harm (HR: 0.83, 95% CI: 0.73–0.95, *p* = 0.007).

### Community

Figure 4 displays the hazard ratios for altered risk of outcomes 1 to 4 weeks following a positive RT-PCR in the community environment. The reference group is negative tests in the community environment only. Those who tested positive were at a significantly increased risk of death (HR: 4.10, 95% CI: 3.38–4.97, *p* =  < 0.001), embolism (HR: 2.80, 95% CI: 1.91–4.21, *p* =  < 0.001), fatigue (HR: 1.76, 95% CI: 1.26–2.46, *p* =  < 0.001), influenza (HR: 10.31, 95% CI: 8.53–12.50, *p* =  < 0.001), and respiratory conditions (HR: 1.57, 95% CI: 1.44–1.72, *p* =  < 0.001). There was an indication of a positive association between volume of sick notes and testing positive (HR: 3.04, 95% CI: 0.88–10.50, *p* = 0.079). However, due to low frequency, this result does not meet the significance threshold (*p* < 0.05), and the confidence interval encompasses the null; thus, evidence of association cannot be provided. They were at a decreased risk of all mental health conditions (HR: 0.79, 95% CI: 0.66–0.94, *p* = 0.007), hospitalisation (HR: 0.55, 95% CI: 0.52–0.60, *p* =  < 0.001), and anxiety, depression, and self-harm (HR: 0.82, 95% CI: 0.68–0.97, *p* = 0.024).

### Hospital

Figure 5 demonstrates the hazard ratios for altered risk of outcomes 1 to 4 weeks following a positive RT-PCR in the hospital environment. The reference group is negative tests in hospital only. Those who tested positive were at a significantly greater risk of death (HR: 2.83, 95% CI: 2.50–3.22, *p* =  < 0.001), fatigue (HR: 1.81, 95% CI: 1.08–3.02, *p* = 0.024), influenza (HR: 3.92, 95% CI: 3.40–4.51, *p* =  < 0.001), and respiratory conditions (HR: 1.50, 95% CI: 1.36–1.65, *p* =  < 0.001) 1 to 4 weeks after the test result. No outcomes that were less likely achieved statistical significance.

### Never tested population

Figure 6 shows the hazard ratios for altered risk of outcomes 1 to 4 weeks following a matched RT-PCR date for individuals who were never tested. The reference group was individuals with negative tests in both the hospital and the community combined. Never tested individuals were significantly less likely to die (HR: 0.13, 95% CI: 0.11–0.16, *p* =  < 0.001) or attend healthcare for anxiety, depression, and self-harm (HR: 0.17, 95% CI: 0.15–0.20, *p* =  < 0.001), diabetes (HR: 0.43, 95% CI: 0.29–0.64, *p* =  < 0.001), embolism (HR: 0.11, 95% CI: 0.08–0.17, *p* =  < 0.001), fatigue (HR: 0.34, 95% CI: 0.25–0.45, *p* =  < 0.001), influenza (HR: 0.18, 95% CI: 0.15–0.22, *p* =  < 0.001), any mental health visit (HR: 0.50, 95% CI: 0.46–0.54, *p* =  < 0.001), or respiratory conditions (HR: 0.23, 95% CI: 0.22–0.25, *p* =  < 0.001). Sick notes were issued to this group less frequently; however, this was non-significant, and the confidence interval encompassed the null (HR: 0.43, 95% CI: 0.12–1.54, *p* = 0.200).

### Outcomes 5 to 24 weeks following a positive RT-PCR

#### All locations

Figure 7 illustrates the hazard ratios for altered risk of outcomes 5 to 24 weeks following a positive RT-PCR test in either the community or hospital environment. The reference group is negative tests in the both the hospital and community environments combined. Those who survived COVID-19 were at a significantly increased risk of embolism (HR: 1.51, 95% CI: 1.13–2.02, *p* = 0.005), fatigue (HR: 1.47, 95% CI: 1.24–1.75, *p* =  < 0.001), and post viral syndrome (HR: 4.57, 95% CI: 1.77–11.8, *p* = 0.002). Conversely, they had a decreased risk of hospitalisation for any reason (HR: 0.90, 95% CI: 0.84–0.96, *p* = 0.003) and mental health healthcare attendances (HR: 0.83, 95% CI: 0.73–0.94, *p* = 0.004).

#### Community

Figure 8 demonstrates the hazard ratios for altered risk of outcomes 5 to 24 weeks following a positive RT-PCR test in the community environment. The reference group is negative tests in the community only. If tested in the community, positive individuals were at increased risk of embolism (HR: 1.50, 95% CI: 1.01–2.21, *p* = 0.043), fatigue (HR: 1.65, 95% CI: 1.35–2.01, *p* =  < 0.001), and post viral syndrome (HR: 4.65, 95% CI: 1.52–14.20, *p* = 0.007). They had a decreased risk of death (HR: 0.72, 95% CI: 0.58–0.91, *p* = 0.005), anxiety, depression, and self-harm (HR: 0.87, 95% CI: 0.77–1.00, *p* = 0.048), any mental health attendance (HR: 0.87, 95% CI: 0.77–0.99, *p* = 0.038), or hospitalisation (HR: 0.82, 95% CI: 0.74–0.90, *p* =  < 0.001) 5 to 24 weeks after the test.

#### Hospital

Figure 9 displays the hazard ratios for altered risk of outcomes 5 to 24 weeks following a positive RT-PCR test in the hospital environment only. The reference group is negative tests in hospital. Following a positive test in hospital, individuals were more likely to attend healthcare for anxiety, depression, and self-harm (HR: 1.20, 95% CI: 1.00–1.45, *p* = 0.053) and embolism (HR: 1.54, 95% CI: 1.01–2.37, *p* = 0.047). They also were more likely to -die during this time than negative controls (HR: 1.22, 95% CI: 1.01–1.47, *p* = 0.037).

#### Never tested population

Figure 10 shows the hazard ratios for altered risk of outcomes 5 to 24 weeks following a matched RT-PCR date, for individuals who never had a COVID-19 test. The reference group is negative tests in the both the hospital and community environments. Compared to negative controls, those who did not receive a RT-PCR were significantly less likely to attend healthcare for anxiety, depression, and self-harm (HR: 0.51, 95% CI: 0.47–0.55, *p* =  < 0.001), diabetes (HR: 0.71, 95% CI: 0.58–0.86, *p* =  < 0.001), embolism (HR: 0.45, 95% CI: 0.34–0.58, *p* =  < 0.001), fatigue (HR: 0.59, 95% CI: 0.51–0.68, *p* =  < 0.001), influenza (HR: 0.46, 95% CI: 0.40–0.54, *p* =  < 0.001), any mental health problems (HR: 0.17, 95% CI: 0.15–0.19, *p* =  < 0.001), and respiratory conditions (HR: 0.72, 95% CI: 0.69–0.76, *p* =  < 0.001). They were also significantly less likely to -attend hospital (HR: 0.47, 95% CI: 0.45–0.50, *p* =  < 0.001) or die (HR: 0.36, 95% CI: 0.32–0.41, *p* =  < 0.001).

#### Life table analysis

Table 3 shows individuals who tested positive in the community have an increased risk or increased trend of a first embolism and first fatigue code occurring through almost the entire follow-up period. Community positive individuals are more at risk of embolism for the first 2 months following a test compared to community negative individuals. There is indication that this trend continues for up to 5 months; however, the 95% confidence intervals encompass the null; thus, definitive evidence cannot be provided. Community positive individuals are more likely to experience fatigue than their negative counterparts for the first four months following a test. The risk ratios continue to exceed 1.0 for the final 2 months of follow-up; however, the confidence intervals encompass the null.

**Table 3:**
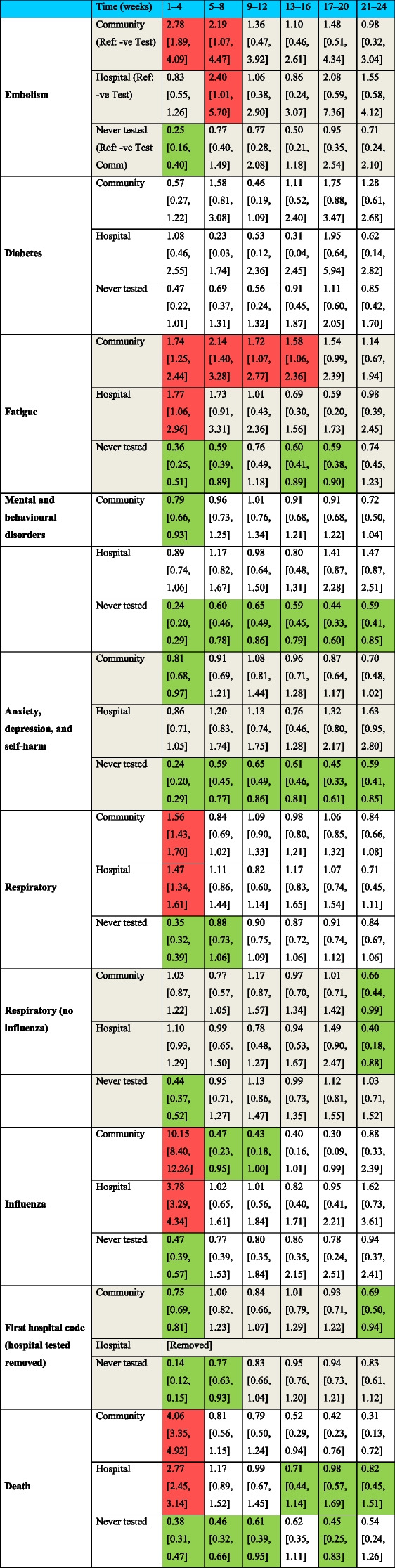
The risk ratios [95% confidence intervals] from life table analysis are presented and show the relative risk of a first event each month following a positive COVID test or from the index date (Never tested). Reference groups are stated in the first row. Sick notes and post viral syndrome removed due to insufficient data [Key: Green: =  < 0.99, Red >  = 1.01 and 95% CI does not cross 1.00 threshold]

Additional file 8: Table S5shows the underlying data for the life tables for the death outcome only due to the infrequency of other outcomes. Death was the most frequently occurring outcome. In the first 4 weeks, 8.34% of individuals died following a positive test in hospital, and 3.00% of hospital negative individuals died in the first 4 weeks. For those in the community, death occurred even less frequently.

## Discussion

### Principle findings

This study examines the healthcare use 1 to 4 and 5 to 24 weeks following COVID-19 using propensity-matched controls. Propensity matching was selected for simplicity of interpretation as it provides one score for matching as opposed to controlling for multiple confounders in a regression analysis. The sample size was of sufficient size to enable high match rates. Figures 3, 4, 5, and 6 and 7, 8, 9, and 10 show hazard ratios for outcomes 1 to 4 and 5 to 24 weeks respectively. It compares individuals who test positive for SARS-CoV-2 with controls who are propensity matched to account for deprivation, comorbidities, numbers in the households, number of previous SARS-CoV-2 tests (i.e. propensity to test positive), gender, age, and local authority area. These findings relate to testing prior to the identification of the Omicron variant and therefore include all variants except Omicron. The cohorts were stratified by individuals testing in the community or hospital and their matches also needed to have been tested in the same stratification. Experiencing COVID-19, even if not accompanied by hospital admission, was associated with an increased risk of fatigue, post-viral illness, and a higher risk of embolism in the community cohort (e.g. code for Venous thromboembolism). The risk of death was greater for COVID-19 positive individuals in the first 4 weeks, but no excess mortality risk was observed after that. Overall, positive individuals were less likely to receive codes for anxiety, depression, or self-harm. However, after 4 weeks, there is an indication that positive individuals tested in hospital may have an increased risk of anxiety, depression, and self-harm. Unfortunately, this finding does not quite meet the threshold for statistical significance (*p* < 0.05), and the confidence interval encompasses the null so evidence cannot confidently be provided for the association and more work would need to be conducted.

### Strengths and limitations

This is a total population cohort of Wales and so is representative of the Welsh population and the Welsh National Health System reporting. The findings are also generalisable to the rest of the UK and trends seen in Wales would be representative of other countries using the NHS but might not be representative of other healthcare systems. The utilisation of propensity matching has the advantage of adjusting for numerous variables, such as accounting for the predisposition to contract COVID-19 and the covariates associated with infection risk. Subsequently, the observations of associated outcomes with surviving COVID-19 are robust. In addition, matching the controls for differences between those tested in the community compared to when they attend a hospital allows adjustment for an individual’s health status, as those tested in hospital are likely more unwell than their community tested counterparts. However, the matching did reduce the sample size of COVID-19 patients from 249,390 to 98,600 which resulted in a loss of 60% of COVID-19 cases who did not have a match, subsequently decreasing the precision for detecting rare events. The test negative design (i.e. comparing people who tested positive to those testing negative) was utilised to better account for potential under-ascertainment and variable testing. The comparison with no test status control group provided greater statistical power for analysis; however, this was potentially at greater risk of bias due to differential testing. For example, those with any respiratory symptoms would have a COVID-19 test so the non-tested group were predominantly non-symptomatic people.

The first study limitation is that the study only investigates the first occurrence and does not reflect total burden or duration of an existing problem. For example, this study showed higher levels of fatigue in those with COVID-19; however, it did not show how long this fatigue lasts for as the analysis gives a time to first mention of a fatigue diagnosis. This study examines engagement with healthcare and so can reflect use and burden to the system due to COVID-19 specifically. However, it cannot capture the unmet needs of people who have a morbidity associated with COVID-19 but do not seek assistance for their illness or cannot access healthcare (e.g. reports of waiting list up by 50% higher in 2021 compared to pre-COVID) [24]. However, both the cases and control are arguably equally as likely to avoid healthcare as the cohorts have been matched and deemed equivalent. Therefore, relative risks should maintain the established relationships. The probably of testing for COVID-19 is dependent on testing capacity in the local area and ability for people to reach testing sites [25]. In addition, this study could not identify diagnoses absent from clinical coding, such as memory loss or brain fog, which have been found to be associated with COVID-19 [26]. Similarly, due to the follow-up period beginning immediately from the date of a positive test, conditions that have been attributed to “influenza” or “respiratory conditions” could be artificially inflated in the first 4 weeks. It is possible that it was the SARS-CoV-2 infection that was being coded and not another distinct respiratory illness that resulted from COVID-19. Finally, although propensity matching was utilised to control for propensity to be tested for COVID-19, the test negative control group will not be equivalent to a general population control; those having a COVID-19 test are more likely to have respiratory symptoms or have symptoms resulting in healthcare encounters. Those who do not have a test for COVID-19 have very low healthcare use in general and thus cannot be propensity matched for previous number of COVID tests. This cohort are also not a general population control equivalent as they have very low healthcare encounters and may have contracted COVID-19 but have not been tested. Consequently, there is no true general population control; instead, this study can compare people who use the healthcare system and who have/do not have a positive COVID-19 test result.

### Comparison with other studies

The finding that those who survive COVID-19 experience higher rates of cardiovascular disease concurs with other published observations such as findings that several cardiovascular disorders are higher in veterans’ data in the USA [27] and higher rates of venous thromboembolism [15] using CPRD data in the UK. However, the finding that there is no overall increase in diagnosis of mental health problems conflicts with literature from the US veterans’ study [26] and a study using the US TriNetX [12] dataset; these both observed higher rates of psychiatric morbidity and mental health diagnosis after COVID-19 [28]. The variation in findings may be due to differences in the variables utilised for the propensity scores to match with test negative patients or disparities in risk of mental health conditions associated with the healthcare system (the USA compared to UK) and with population included, e.g. US veterans cohort vs Wales population cohort. Alternatively, mental health symptoms may have been attributed to the COVID-19 and either not reported to healthcare professionals or were reported as post-COVID symptoms such as fatigue. Those who did not experience COVID-19 may have been more likely to report their mental health symptoms or they may have been attributed to depression; therefore, reporting of mental health symptoms was lower in those with COVID-19.

### Implications and future research

The absolute numbers of contacting their healthcare professional with long-term effects of COVID-19 are low, and there was no increased need for sick notes compared to a matched comparison group after 4 weeks. Therefore, the findings are reassuring that post-COVID adverse consequences do arise but the overall number of people seeking healthcare for this are low. It must be noted though that some adverse events such as embolism are serious and so clinicians should be aware of higher rates for a prolonged period in those who have had COVID-19. It is also important that healthcare professionals consider mental health post-COVID as this may be masked or diagnosed as long-COVID and patients may not receive the appropriate care. In addition, more research is needed to examine the burden to patients who are not seeking healthcare.

## Conclusions

This used a national cohort of people with COVID-19. Cox regression showed that COVID-19 positive individuals were at a significantly increased risk of death, embolism, fatigue, influenza, respiratory conditions, and sick notes in the first 4 weeks after a test. Between 5 to 24 weeks, the risk of embolism and fatigue persisted; they were also at an increased risk from post viral syndrome if tested in the community but not in hospital. However, these individuals were at reduced risk from attending healthcare for mental health conditions. If individuals tested positive in hospital, they were at increased risk from death after 5 to 24 weeks but were at a reduced risk if they tested positive in the community. Life table analysis demonstrates that the absolute risk of these outcomes is very low but some of the burden may be undiagnosed due to sufferers not presenting to a healthcare setting.

## Supplementary Information

**Additional file 1:** **Table S1.** Individual SARS-CoV-2 testing sites included under each testing location. AE – Accident & Emergency, CTU – Clinical Trials Unit, HC – Hospice Care, ICU – Intensive Care Unit.

**Additional file 2:** **Table S2.** Location origins of the codes used to define the outcomes in the study. Additional notes also provided. ADHD – Attention-deficit/hyperactivity disorder, OCD - obsessive compulsive disorder.

**Additional file 3:** **Table S3.** Table of Codes under investigation.

**Additional file 4: Fig S1.** Shows ‘Love Plots’ for the main covariates before and after the propensity matching had taken place for community and hospital tested individuals. WIMD – Welsh Index of Multiple Deprivation.

**Additional file 5:** **Table S4.** Underlying plot data for figures 2 – 9. HR – Hazard Ratio, CI – Confidence Interval.

**Additional file 6:** **Fig S2.** Survival for the full 6-month follow-up for the fatigue outcome. “General population” in the figure refers to the never tested population.

**Additional file 7:** **Fig S3.** Survival for the full 6-month follow-up for the embolism outcome. “General population” in the figure refers to the never tested population.

**Additional file 8:** **Table S5.** Underlying life table data for death outcome only.

## Data Availability

The data that support the findings of this study are available from the SAIL databank, but restrictions apply to the availability of these data, which were used under license for the current study, and so are not publicly available. Data are however available from the authors upon reasonable request and with permission of the SAIL databank. The code behind the analysis has been described in the manuscript. A full version is available from the corresponding author on reasonable request.

## Abbreviations

AR: Absolute Risk
ALF: Anonymised Linking Field
BHF: British Heart Foundation
CCI: Charlson Comorbidity Index
CI: Confidence interval
HR: Hazard Ratio
IGRP: Information Governance Review Panel
ICD-10: International Classification for Diseases 10th Revision
ONS: Office for National Statistics
RR: Risk Ratio
RT-PCR: Reverse Transcription Polymerase Chain Reaction
SAIL: Secure Anonymised Information Linkage
SARS-CoV-2: Severe Acute Respiratory Syndrome Coronavirus 2
WIMD: Welsh Index of Multiple Deprivation
