# Supplementary material for: Healthcare use attributable to COVID-19: a propensity-matched national electronic health records cohort study of 249,390 people in Wales, UK
Source: BMC Med. 2023 Jul 19;21:259. doi: 10.1186/s12916-023-02897-5 (PMC10354936; doi:10.1186/s12916-023-02897-5)
Supplement: Supplementary file 1 — Additional file 1: Table S1. Individual SARS-CoV-2 testing sites included under each testing location. AE – Accident & Emergency, CTU – Clinical Trials Unit, HC – Hospice Care, ICU – Intensive Care Unit. [file 12916_2023_2897_MOESM1_ESM.docx]

|  | **Test Location** | |
| --- | --- | --- |
|  | **Community** | **Hospital** |
| **Test Site** | Care Home | AE |
|  | Community Unit | Hospital |
|  | Covid Unit | ICU |
|  | CTU | Outpatient |
|  | GP | Hospice |
|  | HC Staff |  |
|  | Police |  |
|  | Prison |  |
|  | Self-Home Test |  |
