# Supplementary material for: Healthcare use attributable to COVID-19: a propensity-matched national electronic health records cohort study of 249,390 people in Wales, UK
Source: BMC Med. 2023 Jul 19;21:259. doi: 10.1186/s12916-023-02897-5 (PMC10354936; doi:10.1186/s12916-023-02897-5)
Supplement: Supplementary file 2 — Additional file 2: Table S2. Location origins of the codes used to define the outcomes in the study. Additional notes also provided. ADHD – Attention-deficit/hyperactivity disorder, OCD - obsessive compulsive disorder. [file 12916_2023_2897_MOESM2_ESM.docx]

| Outcome | Codes | | Notes |
| --- | --- | --- | --- |
|  | Primary Care | Secondary Care |  |
| Diabetes | ✓ | 🗶 | Only Primary Care |
| Embolism | ✓ | ✓ |  |
| Fatigue | ✓ | 🗶 | Only Primary Care |
| Influenza (Only) | 🗶 | ✓ | Only Secondary Care |
| Mental and Behavioural Disorders | ✓ | ✓ | Anxiety, Depression, OCD, Schizophrenia,  ADHD, Bipolar, Autism, Self-Harm |
| Anxiety, Depression and Self Harm Only | ✓ | ✓ |  |
| Post Viral Syndrome | ✓ | 🗶 | Only Primary Care |
| Respiratory (All) | ✓ | ✓ | General Respiratory, Mastoid,  Upper and Lower Respiratory Infection, Asthma |
| Respiratory (Influenza Removed) | ✓ | ✓ |  |
| Sick Notes | ✓ | 🗶 | Only Primary Care |
