# Supplementary material for: Healthcare use attributable to COVID-19: a propensity-matched national electronic health records cohort study of 249,390 people in Wales, UK
Source: BMC Med. 2023 Jul 19;21:259. doi: 10.1186/s12916-023-02897-5 (PMC10354936; doi:10.1186/s12916-023-02897-5)
Supplement: Supplementary file 3 — Additional file 3: Table S3. Table of Codes under investigation. [file 12916_2023_2897_MOESM3_ESM.docx]

| **Code** | **Description** | **Class** | **Code type** |
| --- | --- | --- | --- |
| C1061 | Diabetes mellitus, adult onset, + neurological manifestation | Diabetes | Read |
| C1096 | Type II diabetes mellitus with retinopathy | Diabetes | Read |
| C10K0 | Type A insulin resistance without complication | Diabetes | Read |
| C109. | Type II diabetes mellitus | Diabetes | Read |
| C109. | Type 2 diabetes mellitus | Diabetes | Read |
| C104. | Diabetes mellitus with renal manifestation | Diabetes | Read |
| C10FQ | Type 2 diabetes mellitus with exudative maculopathy | Diabetes | Read |
| C109. | NIDDM - Non-insulin dependent diabetes mellitus | Diabetes | Read |
| C10D. | Maturity onset diabetes in youth type 2 | Diabetes | Read |
| C10F1 | Type II diabetes mellitus with ophthalmic complications | Diabetes | Read |
| C109F | Non-insulin-dependent d m with peripheral angiopath | Diabetes | Read |
| C10F9 | Type II diabetes mellitus without complication | Diabetes | Read |
| C10A1 | Malnutrition-related diabetes mellitus with ketoacidosis | Diabetes | Read |
| C10FA | Type II diabetes mellitus with mononeuropathy | Diabetes | Read |
| C1001 | Maturity onset diabetes | Diabetes | Read |
| C105y | Other specified diabetes mellitus with ophthalmic complicatn | Diabetes | Read |
| C1001 | Non-insulin dependent diabetes mellitus | Diabetes | Read |
| C1072 | Diabetes mellitus, adult with gangrene | Diabetes | Read |
| C1095 | Non-insulin dependent diabetes mellitus with gangrene | Diabetes | Read |
| C10FJ | Insulin treated Type II diabetes mellitus | Diabetes | Read |
| C104. | Diabetic nephropathy | Diabetes | Read |
| C10FR | Type II diabetes mellitus with gastroparesis | Diabetes | Read |
| C10z. | Diabetes mellitus with unspecified complication | Diabetes | Read |
| C109F | Type 2 diabetes mellitus with peripheral angiopathy | Diabetes | Read |
| C10D. | Diabetes mellitus autosomal dominant type 2 | Diabetes | Read |
| C10F1 | Type 2 diabetes mellitus with ophthalmic complications | Diabetes | Read |
| C109F | Type II diabetes mellitus with peripheral angiopathy | Diabetes | Read |
| C10F9 | Type 2 diabetes mellitus without complication | Diabetes | Read |
| C10FA | Type 2 diabetes mellitus with mononeuropathy | Diabetes | Read |
| C106z | Diabetes mellitus NOS with neurological manifestation | Diabetes | Read |
| C1096 | Type 2 diabetes mellitus with retinopathy | Diabetes | Read |
| C1095 | Type II diabetes mellitus with gangrene | Diabetes | Read |
| C10FH | Type 2 diabetes mellitus with neuropathic arthropathy | Diabetes | Read |
| C1095 | Type 2 diabetes mellitus with gangrene | Diabetes | Read |
| C10FP | Type 2 diabetes mellitus with ketoacidotic coma | Diabetes | Read |
| C10F0 | Type II diabetes mellitus with renal complications | Diabetes | Read |
| C101z | Diabetes mellitus NOS with ketoacidosis | Diabetes | Read |
| C109E | Non-insulin depend diabetes mellitus with diabetic cataract | Diabetes | Read |
| C10A0 | Malnutrition-related diabetes mellitus with coma | Diabetes | Read |
| C10F8 | Metabolic syndrome X | Diabetes | Read |
| C1011 | Diabetes mellitus, adult onset, with ketoacidosis | Diabetes | Read |
| C1071 | Diabetes mellitus, adult, + peripheral circulatory disorder | Diabetes | Read |
| C1094 | Non-insulin dependent diabetes mellitus with ulcer | Diabetes | Read |
| C10J. | Insulin autoimmune syndrome | Diabetes | Read |
| C105. | Diabetes mellitus with ophthalmic manifestation | Diabetes | Read |
| C10FQ | Type II diabetes mellitus with exudative maculopathy | Diabetes | Read |
| C10F0 | Type 2 diabetes mellitus with renal complications | Diabetes | Read |
| C109E | Type II diabetes mellitus with diabetic cataract | Diabetes | Read |
| C10F8 | Reaven's syndrome | Diabetes | Read |
| C109E | Type 2 diabetes mellitus with diabetic cataract | Diabetes | Read |
| C106y | Other specified diabetes mellitus with neurological comps | Diabetes | Read |
| C10FK | Hyperosmolar non-ketotic state in type 2 diabetes mellitus | Diabetes | Read |
| C1090 | Type 2 diabetes mellitus with renal complications | Diabetes | Read |
| C1090 | Type II diabetes mellitus with renal complications | Diabetes | Read |
| C10FS | Maternally inherited diabetes mellitus | Diabetes | Read |
| C10F3 | Type II diabetes mellitus with multiple complications | Diabetes | Read |
| C10y. | Diabetes mellitus with other specified manifestation | Diabetes | Read |
| C109H | Non-insulin dependent d m with neuropathic arthropathy | Diabetes | Read |
| C10C. | Diabetes mellitus autosomal dominant | Diabetes | Read |
| C10A3 | Malnutrit-related diabetes mellitus wth ophthalmic complicat | Diabetes | Read |
| C10FC | Type II diabetes mellitus with nephropathy | Diabetes | Read |
| C10zy | Other specified diabetes mellitus with unspecified comps | Diabetes | Read |
| C1098 | Reaven's syndrome | Diabetes | Read |
| C1051 | Diabetes mellitus, adult onset, + ophthalmic manifestation | Diabetes | Read |
| C1074 | NIDDM with peripheral circulatory disorder | Diabetes | Read |
| C10FD | Type II diabetes mellitus with hypoglycaemic coma | Diabetes | Read |
| C103. | Diabetes mellitus with ketoacidotic coma | Diabetes | Read |
| C109H | Type 2 diabetes mellitus with neuropathic arthropathy | Diabetes | Read |
| C10ER | Latent autoimmune diabetes mellitus in adult | Diabetes | Read |
| C10F3 | Type 2 diabetes mellitus with multiple complications | Diabetes | Read |
| C10FL | Type II diabetes mellitus with persistent proteinuria | Diabetes | Read |
| C10H. | Diabetes mellitus induced by non-steroid drugs | Diabetes | Read |
| C109H | Type II diabetes mellitus with neuropathic arthropathy | Diabetes | Read |
| C104y | Other specified diabetes mellitus with renal complications | Diabetes | Read |
| C10FC | Type 2 diabetes mellitus with nephropathy | Diabetes | Read |
| C10FJ | Insulin treated Type 2 diabetes mellitus | Diabetes | Read |
| C10FR | Type 2 diabetes mellitus with gastroparesis | Diabetes | Read |
| C10P1 | Type 2 diabetes mellitus in remission | Diabetes | Read |
| C10F2 | Type II diabetes mellitus with neurological complications | Diabetes | Read |
| C109G | Non-insulin dependent diabetes mellitus with arthropathy | Diabetes | Read |
| C10A2 | Malnutrition-related diabetes mellitus with renal complicatn | Diabetes | Read |
| C105z | Diabetes mellitus NOS with ophthalmic manifestation | Diabetes | Read |
| C10FB | Type II diabetes mellitus with polyneuropathy | Diabetes | Read |
| C1097 | Non-insulin dependent diabetes mellitus - poor control | Diabetes | Read |
| C1096 | Non-insulin-dependent diabetes mellitus with retinopathy | Diabetes | Read |
| C10FK | Hyperosmolar non-ketotic state in type II diabetes mellitus | Diabetes | Read |
| C10N. | Secondary diabetes mellitus | Diabetes | Read |
| C109. | Non-insulin dependent diabetes mellitus | Diabetes | Read |
| C10P1 | Type II diabetes mellitus in remission | Diabetes | Read |
| C10F2 | Type 2 diabetes mellitus with neurological complications | Diabetes | Read |
| C10J0 |  | Diabetes | Read |
| C109G |  | Diabetes | Read |
| C109G | Type 2 diabetes mellitus with arthropathy | Diabetes | Read |
| C10C. | Maturity onset diabetes in youth | Diabetes | Read |
| C100z | Diabetes mellitus NOS with no mention of complication | Diabetes | Read |
| C10.. | Diabetes mellitus | Diabetes | Read |
| C1001 | Diabetes mellitus, adult onset, no mention of complication | Diabetes | Read |
| C10FB | Type 2 diabetes mellitus with polyneuropathy | Diabetes | Read |
| C1097 | Type II diabetes mellitus - poor control | Diabetes | Read |
| C1097 | Type 2 diabetes mellitus - poor control | Diabetes | Read |
| C1092 | Type 2 diabetes mellitus with neurological complications | Diabetes | Read |
| C1092 | Type II diabetes mellitus with neurological complications | Diabetes | Read |
| C10FE | Type 2 diabetes mellitus with diabetic cataract | Diabetes | Read |
| C109J | Insulin treated Type 2 diabetes mellitus | Diabetes | Read |
| C10FM | Type 2 diabetes mellitus with persistent microalbuminuria | Diabetes | Read |
| C10G. | Secondary pancreatic diabetes mellitus | Diabetes | Read |
| C10F5 | Type II diabetes mellitus with gangrene | Diabetes | Read |
| C103z | Diabetes mellitus NOS with ketoacidotic coma | Diabetes | Read |
| C109B | Non-insulin dependent diabetes mellitus with polyneuropathy | Diabetes | Read |
| C10A5 | Malnutritn-relat diabetes melitus wth periph circul complctn | Diabetes | Read |
| C1031 | Diabetes mellitus, adult onset, with ketoacidotic coma | Diabetes | Read |
| C10L. |  | Diabetes | Read |
| C10N1 | Cystic fibrosis related diabetes mellitus | Diabetes | Read |
| C107. | Diabetes mellitus with peripheral circulatory disorder | Diabetes | Read |
| C1091 | Non-insulin-dependent diabetes mellitus with ophthalm comps | Diabetes | Read |
| C10FF | Type II diabetes mellitus with peripheral angiopathy | Diabetes | Read |
| C109J | Insulin treated Type II diabetes mellitus | Diabetes | Read |
| C109J | Insulin treated non-insulin dependent diabetes mellitus | Diabetes | Read |
| C10A. |  | Diabetes | Read |
| C10FN | Type II diabetes mellitus with ketoacidosis | Diabetes | Read |
| C10H0 | DM induced by non-steroid drugs without complication | Diabetes | Read |
| C109B | Type 2 diabetes mellitus with polyneuropathy | Diabetes | Read |
| C10F5 | Type 2 diabetes mellitus with gangrene | Diabetes | Read |
| C109B | Type II diabetes mellitus with polyneuropathy | Diabetes | Read |
| C108y | Other specified diabetes mellitus with multiple comps | Diabetes | Read |
| C10yz | Diabetes mellitus NOS with other specified manifestation | Diabetes | Read |
| C102. | Diabetes mellitus with hyperosmolar coma | Diabetes | Read |
| C1041 | Diabetes mellitus, adult onset, with renal manifestation | Diabetes | Read |
| C107. | Diabetes mellitus with gangrene | Diabetes | Read |
| C1091 | Type II diabetes mellitus with ophthalmic complications | Diabetes | Read |
| C10FD | Type 2 diabetes mellitus with hypoglycaemic coma | Diabetes | Read |
| C10M. | Lipoatrophic diabetes mellitus | Diabetes | Read |
| C1091 | Type 2 diabetes mellitus with ophthalmic complications | Diabetes | Read |
| C107. | Diabetes with gangrene | Diabetes | Read |
| C10FL | Type 2 diabetes mellitus with persistent proteinuria | Diabetes | Read |
| C10F4 | Type II diabetes mellitus with ulcer | Diabetes | Read |
| C103y | Other specified diabetes mellitus with coma | Diabetes | Read |
| C109A | Non-insulin dependent diabetes mellitus with mononeuropathy | Diabetes | Read |
| C10A4 |  | Diabetes | Read |
| C1099 | Non-insulin-dependent diabetes mellitus without complication | Diabetes | Read |
| C10zz | Diabetes mellitus NOS with unspecified complication | Diabetes | Read |
| C10z1 | Diabetes mellitus, adult onset, + unspecified complication | Diabetes | Read |
| C10N0 | Secondary diabetes mellitus without complication | Diabetes | Read |
| C10FE | Type II diabetes mellitus with diabetic cataract | Diabetes | Read |
| C1090 | Non-insulin-dependent diabetes mellitus with renal comps | Diabetes | Read |
| C10B. | Diabetes mellitus induced by steroids | Diabetes | Read |
| C10FM | Type II diabetes mellitus with persistent microalbuminuria | Diabetes | Read |
| C10F4 | Type 2 diabetes mellitus with ulcer | Diabetes | Read |
| C104z | Diabetes mellitus with nephropathy NOS | Diabetes | Read |
| C109A |  | Diabetes | Read |
| C109A |  | Diabetes | Read |
| C1099 |  | Diabetes | Read |
| C1099 | Type 2 diabetes mellitus without complication | Diabetes | Read |
| C10yy | Other specified diabetes mellitus with other spec comps | Diabetes | Read |
| C1094 | Type 2 diabetes mellitus with ulcer | Diabetes | Read |
| C1094 | Type II diabetes mellitus with ulcer | Diabetes | Read |
| C10K. | Type A insulin resistance | Diabetes | Read |
| C10FG | Type 2 diabetes mellitus with arthropathy | Diabetes | Read |
| C10G0 | Secondary pancreatic diabetes mellitus without complication | Diabetes | Read |
| C101y | Other specified diabetes mellitus with ketoacidosis | Diabetes | Read |
| C109D | Non-insulin dependent diabetes mellitus with hypoglyca coma | Diabetes | Read |
| C10F7 | Type II diabetes mellitus - poor control | Diabetes | Read |
| C10A7 |  | Diabetes | Read |
| C107z | Diabetes mellitus NOS with peripheral circulatory disorder | Diabetes | Read |
| C10FH | Type II diabetes mellitus with neuropathic arthropathy | Diabetes | Read |
| C10L0 |  | Diabetes | Read |
| C1093 | Non-insulin-dependent diabetes mellitus with multiple comps | Diabetes | Read |
| C10FP | Type II diabetes mellitus with ketoacidotic coma | Diabetes | Read |
| C10P. | Diabetes mellitus in remission | Diabetes | Read |
| C109D | Type 2 diabetes mellitus with hypoglycaemic coma | Diabetes | Read |
| C102z | Diabetes mellitus NOS with hyperosmolar coma | Diabetes | Read |
| C109D | Type II diabetes mellitus with hypoglycaemic coma | Diabetes | Read |
| C10F7 | Type 2 diabetes mellitus - poor control | Diabetes | Read |
| C100. | Diabetes mellitus with no mention of complication | Diabetes | Read |
| C1021 | Diabetes mellitus, adult onset, with hyperosmolar coma | Diabetes | Read |
| C10AX |  | Diabetes | Read |
| C10y1 | Diabetes mellitus, adult, + other specified manifestation | Diabetes | Read |
| C1093 | Type II diabetes mellitus with multiple complications | Diabetes | Read |
| C10M0 |  | Diabetes | Read |
| C1093 | Type 2 diabetes mellitus with multiple complications | Diabetes | Read |
| C106. | Diabetes mellitus with neurological manifestation | Diabetes | Read |
| C10FF | Type 2 diabetes mellitus with peripheral angiopathy | Diabetes | Read |
| C10F. | Type II diabetes mellitus | Diabetes | Read |
| C109K | Hyperosmolar non-ketotic state in type 2 diabetes mellitus | Diabetes | Read |
| C10A. | Malnutrition-related diabetes mellitus | Diabetes | Read |
| C10FN | Type 2 diabetes mellitus with ketoacidosis | Diabetes | Read |
| C109C | Non-insulin dependent diabetes mellitus with nephropathy | Diabetes | Read |
| C10A6 |  | Diabetes | Read |
| C10F6 | Type II diabetes mellitus with retinopathy | Diabetes | Read |
| C107y | Other specified diabetes mellitus with periph circ comps | Diabetes | Read |
| C106. | Diabetes mellitus with polyneuropathy | Diabetes | Read |
| C101. | Diabetes mellitus with ketoacidosis | Diabetes | Read |
| C1092 | Non-insulin-dependent diabetes mellitus with neuro comps | Diabetes | Read |
| C106. | Diabetic amyotrophy | Diabetes | Read |
| C106. | Diabetes mellitus with neuropathy | Diabetes | Read |
| C10FG | Type II diabetes mellitus with arthropathy | Diabetes | Read |
| C10F. | Type 2 diabetes mellitus | Diabetes | Read |
| C109C | Type II diabetes mellitus with nephropathy | Diabetes | Read |
| C10B0 | Steroid induced diabetes mellitus without complication | Diabetes | Read |
| C109C | Type 2 diabetes mellitus with nephropathy | Diabetes | Read |
| C10F6 | Type 2 diabetes mellitus with retinopathy | Diabetes | Read |
| C108z | Unspecified diabetes mellitus with multiple complications | Diabetes | Read |
| C10AW |  | Diabetes | Read |
| C10ED | Insulin dependent diabetes mellitus with nephropathy | Diabetes | Read |
| C10y0 | Diabetes mellitus, juvenile, + other specified manifestation | Diabetes | Read |
| C10ED | Type I diabetes mellitus with nephropathy | Diabetes | Read |
| C1084 | Unstable insulin dependent diabetes mellitus | Diabetes | Read |
| C10EH |  | Diabetes | Read |
| C10EH |  | Diabetes | Read |
| C1080 | Insulin-dependent diabetes mellitus with renal complications | Diabetes | Read |
| C10P0 | Type 1 diabetes mellitus in remission | Diabetes | Read |
| C10EL |  | Diabetes | Read |
| C108E | Type 1 diabetes mellitus with hypoglycaemic coma | Diabetes | Read |
| C10EP | Type I diabetes mellitus with exudative maculopathy | Diabetes | Read |
| C108A |  | Diabetes | Read |
| C108E | Type I diabetes mellitus with hypoglycaemic coma | Diabetes | Read |
| C10E3 | Type 1 diabetes mellitus with multiple complications | Diabetes | Read |
| C108A | Type I diabetes mellitus without complication | Diabetes | Read |
| C10E7 | Type 1 diabetes mellitus with retinopathy | Diabetes | Read |
| C1089 | Type 1 diabetes mellitus maturity onset | Diabetes | Read |
| C1085 | Type 1 diabetes mellitus with ulcer | Diabetes | Read |
| C1089 | Type I diabetes mellitus maturity onset | Diabetes | Read |
| C1084 | Unstable type 1 diabetes mellitus | Diabetes | Read |
| C10ED | Type 1 diabetes mellitus with nephropathy | Diabetes | Read |
| C1084 | Unstable type I diabetes mellitus | Diabetes | Read |
| C1030 | Diabetes mellitus, juvenile type, with ketoacidotic coma | Diabetes | Read |
| C10EH | Type 1 diabetes mellitus with arthropathy | Diabetes | Read |
| C1080 | Type 1 diabetes mellitus with renal complications | Diabetes | Read |
| C1080 | Type I diabetes mellitus with renal complications | Diabetes | Read |
| C10EL | Type 1 diabetes mellitus with persistent microalbuminuria | Diabetes | Read |
| C10P0 | Type I diabetes mellitus in remission | Diabetes | Read |
| C10E0 | Insulin-dependent diabetes mellitus with renal complications | Diabetes | Read |
| C10EP | Type 1 diabetes mellitus with exudative maculopathy | Diabetes | Read |
| C10E0 | Type I diabetes mellitus with renal complications | Diabetes | Read |
| C108H | Insulin dependent diabetes mellitus with arthropathy | Diabetes | Read |
| C10E4 | Unstable insulin dependent diabetes mellitus | Diabetes | Read |
| C10E4 | Unstable type I diabetes mellitus | Diabetes | Read |
| C108D | Insulin dependent diabetes mellitus with nephropathy | Diabetes | Read |
| C10E8 | Insulin dependent diabetes mellitus - poor control | Diabetes | Read |
| C10E8 | Type I diabetes mellitus - poor control | Diabetes | Read |
| C1000 | Diabetes mellitus, juvenile type, no mention of complication | Diabetes | Read |
| C1088 | Insulin dependent diabetes mellitus - poor control | Diabetes | Read |
| C1060 | Diabetes mellitus, juvenile, + neurological manifestation | Diabetes | Read |
| C1083 | Insulin dependent diabetes mellitus with multiple complicatn | Diabetes | Read |
| C10EC | Insulin dependent diabetes mellitus with polyneuropathy | Diabetes | Read |
| C10EC |  | Diabetes | Read |
| C10EG |  | Diabetes | Read |
| C10EG |  | Diabetes | Read |
| C10E. | Type 1 diabetes mellitus | Diabetes | Read |
| C10EK | Type I diabetes mellitus with persistent proteinuria | Diabetes | Read |
| C10E2 | Type 1 diabetes mellitus with neurological complications | Diabetes | Read |
| C108H |  | Diabetes | Read |
| C108H | Type I diabetes mellitus with arthropathy | Diabetes | Read |
| C10E6 | Type 1 diabetes mellitus with gangrene | Diabetes | Read |
| C108D | Type 1 diabetes mellitus with nephropathy | Diabetes | Read |
| C108D | Type I diabetes mellitus with nephropathy | Diabetes | Read |
| C1000 | Insulin dependent diabetes mellitus | Diabetes | Read |
| C10EB | Type 1 diabetes mellitus with mononeuropathy | Diabetes | Read |
| C1088 | Type 1 diabetes mellitus - poor control | Diabetes | Read |
| C1088 | Type I diabetes mellitus - poor control | Diabetes | Read |
| C1083 |  | Diabetes | Read |
| C1083 | Type 1 diabetes mellitus with multiple complications | Diabetes | Read |
| C10EC | Type 1 diabetes mellitus with polyneuropathy | Diabetes | Read |
| C10EG | Type 1 diabetes mellitus with peripheral angiopathy | Diabetes | Read |
| C10EK | Type 1 diabetes mellitus with persistent proteinuria | Diabetes | Read |
| C108G | Insulin dependent diab mell with peripheral angiopathy | Diabetes | Read |
| C108C | Insulin dependent diabetes mellitus with polyneuropathy | Diabetes | Read |
| C10E3 | Insulin dependent diabetes mellitus with multiple complicat | Diabetes | Read |
| C10E3 | Type I diabetes mellitus with multiple complications | Diabetes | Read |
| C10E7 | Insulin dependent diabetes mellitus with retinopathy | Diabetes | Read |
| C10E7 | Type I diabetes mellitus with retinopathy | Diabetes | Read |
| C1087 | Insulin dependent diabetes mellitus with retinopathy | Diabetes | Read |
| C10EF | Type I diabetes mellitus with diabetic cataract | Diabetes | Read |
| C1082 | Insulin-dependent diabetes mellitus with neurological comps | Diabetes | Read |
| C10EB |  | Diabetes | Read |
| C10EJ |  | Diabetes | Read |
| C108. | Insulin dependent diabetes mellitus | Diabetes | Read |
| C1040 | Diabetes mellitus, juvenile type, with renal manifestation | Diabetes | Read |
| C10EF | Insulin dependent diabetes mellitus with diabetic cataract | Diabetes | Read |
| C10EN |  | Diabetes | Read |
| C10EJ | Insulin dependent diab mell with neuropathic arthropathy | Diabetes | Read |
| C108G |  | Diabetes | Read |
| C10E1 | Type 1 diabetes mellitus with ophthalmic complications | Diabetes | Read |
| C108G |  | Diabetes | Read |
| C108C |  | Diabetes | Read |
| C10E5 | Type 1 diabetes mellitus with ulcer | Diabetes | Read |
| C108C |  | Diabetes | Read |
| C10E9 | Type 1 diabetes mellitus maturity onset | Diabetes | Read |
| C1010 | Diabetes mellitus, juvenile type, with ketoacidosis | Diabetes | Read |
| C1087 | Type I diabetes mellitus with retinopathy | Diabetes | Read |
| C10EA | Type 1 diabetes mellitus without complication | Diabetes | Read |
| C1087 | Type 1 diabetes mellitus with retinopathy | Diabetes | Read |
| C1082 | Type 1 diabetes mellitus with neurological complications | Diabetes | Read |
| C10EF | Type 1 diabetes mellitus with diabetic cataract | Diabetes | Read |
| C1082 |  | Diabetes | Read |
| C10z0 | Diabetes mellitus, juvenile type, + unspecified complication | Diabetes | Read |
| C108. | Type 1 diabetes mellitus | Diabetes | Read |
| C10EJ | Type 1 diabetes mellitus with neuropathic arthropathy | Diabetes | Read |
| C108. | IDDM-Insulin dependent diabetes mellitus | Diabetes | Read |
| C1070 | Diabetes mellitus, juvenile +peripheral circulatory disorder | Diabetes | Read |
| C108. | Type I diabetes mellitus | Diabetes | Read |
| C10EN | Type 1 diabetes mellitus with ketoacidotic coma | Diabetes | Read |
| C10E. | Type I diabetes mellitus | Diabetes | Read |
| C108J | Insulin dependent diab mell with neuropathic arthropathy | Diabetes | Read |
| C10E2 |  | Diabetes | Read |
| C108F | Insulin dependent diabetes mellitus with diabetic cataract | Diabetes | Read |
| C10E. | Insulin dependent diabetes mellitus | Diabetes | Read |
| C10E6 | Type I diabetes mellitus with gangrene | Diabetes | Read |
| C108B | Insulin dependent diabetes mellitus with mononeuropathy | Diabetes | Read |
| C10E2 | Insulin-dependent diabetes mellitus with neurological comps | Diabetes | Read |
| C10E6 | Insulin dependent diabetes mellitus with gangrene | Diabetes | Read |
| C10EB |  | Diabetes | Read |
| C1086 | Insulin dependent diabetes mellitus with gangrene | Diabetes | Read |
| C10EE | Insulin dependent diabetes mellitus with hypoglycaemic coma | Diabetes | Read |
| C10EE |  | Diabetes | Read |
| C1085 | Insulin dependent diabetes mellitus with ulcer | Diabetes | Read |
| C1081 | Insulin-dependent diabetes mellitus with ophthalmic comps | Diabetes | Read |
| C108J | Type 1 diabetes mellitus with neuropathic arthropathy | Diabetes | Read |
| C10EM | Type I diabetes mellitus with ketoacidosis | Diabetes | Read |
| C108J | Type I diabetes mellitus with neuropathic arthropathy | Diabetes | Read |
| C108F |  | Diabetes | Read |
| C108F | Type I diabetes mellitus with diabetic cataract | Diabetes | Read |
| C10EQ | Type I diabetes mellitus with gastroparesis | Diabetes | Read |
| C10E0 | Type 1 diabetes mellitus with renal complications | Diabetes | Read |
| C108B |  | Diabetes | Read |
| C108B | Type I diabetes mellitus with mononeuropathy | Diabetes | Read |
| C10E4 | Unstable type 1 diabetes mellitus | Diabetes | Read |
| C10E8 | Type 1 diabetes mellitus - poor control | Diabetes | Read |
| C1086 |  | Diabetes | Read |
| C1086 |  | Diabetes | Read |
| C10EE | Type 1 diabetes mellitus with hypoglycaemic coma | Diabetes | Read |
| C1050 | Diabetes mellitus, juvenile type, + ophthalmic manifestation | Diabetes | Read |
| C1081 | Type 1 diabetes mellitus with ophthalmic complications | Diabetes | Read |
| C1085 | Type I diabetes mellitus with ulcer | Diabetes | Read |
| C1073 | IDDM with peripheral circulatory disorder | Diabetes | Read |
| C1081 |  | Diabetes | Read |
| C10EM | Type 1 diabetes mellitus with ketoacidosis | Diabetes | Read |
| C10E1 | Insulin-dependent diabetes mellitus with ophthalmic comps | Diabetes | Read |
| C10C. | Maturity onset diabetes in youth type 1 | Diabetes | Read |
| C10E1 | Type I diabetes mellitus with ophthalmic complications | Diabetes | Read |
| C10EQ | Type 1 diabetes mellitus with gastroparesis | Diabetes | Read |
| C10E5 | Insulin dependent diabetes mellitus with ulcer | Diabetes | Read |
| C10E5 | Type I diabetes mellitus with ulcer | Diabetes | Read |
| C108E | Insulin dependent diabetes mellitus with hypoglycaemic coma | Diabetes | Read |
| C10E9 | Insulin dependent diabetes maturity onset | Diabetes | Read |
| C10E9 | Type I diabetes mellitus maturity onset | Diabetes | Read |
| C108A | Insulin-dependent diabetes without complication | Diabetes | Read |
| C1020 | Diabetes mellitus, juvenile type, with hyperosmolar coma | Diabetes | Read |
| C10EA | Insulin-dependent diabetes without complication | Diabetes | Read |
| C10EA | Type I diabetes mellitus without complication | Diabetes | Read |
| C1089 | Insulin dependent diabetes maturity onset | Diabetes | Read |
| C1061 | Diabetes mellitus, adult onset, + neurological manifestation | Diabetes | Read |
| C1096 | Type II diabetes mellitus with retinopathy | Diabetes | Read |
| C1092 | Type 2 diabetes mellitus with neurological complications | Diabetes | Read |
| C109. | Type II diabetes mellitus | Diabetes | Read |
| C109. | Type 2 diabetes mellitus | Diabetes | Read |
| C1092 | Type II diabetes mellitus with neurological complications | Diabetes | Read |
| C10FE | Type 2 diabetes mellitus with diabetic cataract | Diabetes | Read |
| C10FQ | Type 2 diabetes mellitus with exudative maculopathy | Diabetes | Read |
| C109. | NIDDM - Non-insulin dependent diabetes mellitus | Diabetes | Read |
| C109J | Insulin treated Type 2 diabetes mellitus | Diabetes | Read |
| C10FM | Type 2 diabetes mellitus with persistent microalbuminuria | Diabetes | Read |
| C10D. | Maturity onset diabetes in youth type 2 | Diabetes | Read |
| C10F1 | Type II diabetes mellitus with ophthalmic complications | Diabetes | Read |
| C109F | Non-insulin-dependent d m with peripheral angiopath | Diabetes | Read |
| C10F9 | Type II diabetes mellitus without complication | Diabetes | Read |
| C10F5 | Type II diabetes mellitus with gangrene | Diabetes | Read |
| C109B | Non-insulin dependent diabetes mellitus with polyneuropathy | Diabetes | Read |
| C10FA | Type II diabetes mellitus with mononeuropathy | Diabetes | Read |
| C1031 | Diabetes mellitus, adult onset, with ketoacidotic coma | Diabetes | Read |
| C1001 | Non-insulin dependent diabetes mellitus | Diabetes | Read |
| C1095 | Non-insulin dependent diabetes mellitus with gangrene | Diabetes | Read |
| C10FJ | Insulin treated Type II diabetes mellitus | Diabetes | Read |
| C1091 | Non-insulin-dependent diabetes mellitus with ophthalm comps | Diabetes | Read |
| C10FF | Type II diabetes mellitus with peripheral angiopathy | Diabetes | Read |
| C109J | Insulin treated Type II diabetes mellitus | Diabetes | Read |
| C10FR | Type II diabetes mellitus with gastroparesis | Diabetes | Read |
| C109J | Insulin treated non-insulin dependent diabetes mellitus | Diabetes | Read |
| C109F | Type 2 diabetes mellitus with peripheral angiopathy | Diabetes | Read |
| C10D. | Diabetes mellitus autosomal dominant type 2 | Diabetes | Read |
| C10F1 | Type 2 diabetes mellitus with ophthalmic complications | Diabetes | Read |
| C10FN | Type II diabetes mellitus with ketoacidosis | Diabetes | Read |
| C109F | Type II diabetes mellitus with peripheral angiopathy | Diabetes | Read |
| C10F9 | Type 2 diabetes mellitus without complication | Diabetes | Read |
| C109B | Type 2 diabetes mellitus with polyneuropathy | Diabetes | Read |
| C10F5 | Type 2 diabetes mellitus with gangrene | Diabetes | Read |
| C109B | Type II diabetes mellitus with polyneuropathy | Diabetes | Read |
| C10FA | Type 2 diabetes mellitus with mononeuropathy | Diabetes | Read |
| C1096 | Type 2 diabetes mellitus with retinopathy | Diabetes | Read |
| C1095 | Type II diabetes mellitus with gangrene | Diabetes | Read |
| C10FH | Type 2 diabetes mellitus with neuropathic arthropathy | Diabetes | Read |
| C1095 | Type 2 diabetes mellitus with gangrene | Diabetes | Read |
| C1041 | Diabetes mellitus, adult onset, with renal manifestation | Diabetes | Read |
| C1091 | Type II diabetes mellitus with ophthalmic complications | Diabetes | Read |
| C10FD | Type 2 diabetes mellitus with hypoglycaemic coma | Diabetes | Read |
| C1091 | Type 2 diabetes mellitus with ophthalmic complications | Diabetes | Read |
| C10FP | Type 2 diabetes mellitus with ketoacidotic coma | Diabetes | Read |
| C10FL | Type 2 diabetes mellitus with persistent proteinuria | Diabetes | Read |
| C10F0 | Type II diabetes mellitus with renal complications | Diabetes | Read |
| C109E | Non-insulin depend diabetes mellitus with diabetic cataract | Diabetes | Read |
| C10F8 | Metabolic syndrome X | Diabetes | Read |
| C10F4 | Type II diabetes mellitus with ulcer | Diabetes | Read |
| C109A | Non-insulin dependent diabetes mellitus with mononeuropathy | Diabetes | Read |
| C1011 | Diabetes mellitus, adult onset, with ketoacidosis | Diabetes | Read |
| C1099 | Non-insulin-dependent diabetes mellitus without complication | Diabetes | Read |
| C1071 | Diabetes mellitus, adult, + peripheral circulatory disorder | Diabetes | Read |
| C10z1 | Diabetes mellitus, adult onset, + unspecified complication | Diabetes | Read |
| C1094 | Non-insulin dependent diabetes mellitus with ulcer | Diabetes | Read |
| C10FE | Type II diabetes mellitus with diabetic cataract | Diabetes | Read |
| C1090 | Non-insulin-dependent diabetes mellitus with renal comps | Diabetes | Read |
| C10FQ | Type II diabetes mellitus with exudative maculopathy | Diabetes | Read |
| C10FM | Type II diabetes mellitus with persistent microalbuminuria | Diabetes | Read |
| C10F0 | Type 2 diabetes mellitus with renal complications | Diabetes | Read |
| C109E | Type II diabetes mellitus with diabetic cataract | Diabetes | Read |
| C109E | Type 2 diabetes mellitus with diabetic cataract | Diabetes | Read |
| C10F4 | Type 2 diabetes mellitus with ulcer | Diabetes | Read |
| C109A |  | Diabetes | Read |
| C109A |  | Diabetes | Read |
| C1099 |  | Diabetes | Read |
| C1099 | Type 2 diabetes mellitus without complication | Diabetes | Read |
| C1094 | Type 2 diabetes mellitus with ulcer | Diabetes | Read |
| C1094 | Type II diabetes mellitus with ulcer | Diabetes | Read |
| C10FK | Hyperosmolar non-ketotic state in type 2 diabetes mellitus | Diabetes | Read |
| C1090 | Type 2 diabetes mellitus with renal complications | Diabetes | Read |
| C1090 | Type II diabetes mellitus with renal complications | Diabetes | Read |
| C10FG | Type 2 diabetes mellitus with arthropathy | Diabetes | Read |
| C10F3 | Type II diabetes mellitus with multiple complications | Diabetes | Read |
| C109H | Non-insulin dependent d m with neuropathic arthropathy | Diabetes | Read |
| C109D | Non-insulin dependent diabetes mellitus with hypoglyca coma | Diabetes | Read |
| C10F7 | Type II diabetes mellitus - poor control | Diabetes | Read |
| C10FC | Type II diabetes mellitus with nephropathy | Diabetes | Read |
| C10FH | Type II diabetes mellitus with neuropathic arthropathy | Diabetes | Read |
| C1051 | Diabetes mellitus, adult onset, + ophthalmic manifestation | Diabetes | Read |
| C1074 | NIDDM with peripheral circulatory disorder | Diabetes | Read |
| C1093 | Non-insulin-dependent diabetes mellitus with multiple comps | Diabetes | Read |
| C10FD | Type II diabetes mellitus with hypoglycaemic coma | Diabetes | Read |
| C10FP | Type II diabetes mellitus with ketoacidotic coma | Diabetes | Read |
| C109H | Type 2 diabetes mellitus with neuropathic arthropathy | Diabetes | Read |
| C10F3 | Type 2 diabetes mellitus with multiple complications | Diabetes | Read |
| C10FL | Type II diabetes mellitus with persistent proteinuria | Diabetes | Read |
| C109H | Type II diabetes mellitus with neuropathic arthropathy | Diabetes | Read |
| C109D | Type 2 diabetes mellitus with hypoglycaemic coma | Diabetes | Read |
| C109D | Type II diabetes mellitus with hypoglycaemic coma | Diabetes | Read |
| C10F7 | Type 2 diabetes mellitus - poor control | Diabetes | Read |
| C1021 | Diabetes mellitus, adult onset, with hyperosmolar coma | Diabetes | Read |
| C10FC | Type 2 diabetes mellitus with nephropathy | Diabetes | Read |
| C10y1 | Diabetes mellitus, adult, + other specified manifestation | Diabetes | Read |
| C1093 | Type II diabetes mellitus with multiple complications | Diabetes | Read |
| C10FJ | Insulin treated Type 2 diabetes mellitus | Diabetes | Read |
| C1093 | Type 2 diabetes mellitus with multiple complications | Diabetes | Read |
| C10FF | Type 2 diabetes mellitus with peripheral angiopathy | Diabetes | Read |
| C10F. | Type II diabetes mellitus | Diabetes | Read |
| C109K | Hyperosmolar non-ketotic state in type 2 diabetes mellitus | Diabetes | Read |
| C10FR | Type 2 diabetes mellitus with gastroparesis | Diabetes | Read |
| C10P1 | Type 2 diabetes mellitus in remission | Diabetes | Read |
| C10F2 | Type II diabetes mellitus with neurological complications | Diabetes | Read |
| C109G | Non-insulin dependent diabetes mellitus with arthropathy | Diabetes | Read |
| C10FN | Type 2 diabetes mellitus with ketoacidosis | Diabetes | Read |
| C109C | Non-insulin dependent diabetes mellitus with nephropathy | Diabetes | Read |
| C10F6 | Type II diabetes mellitus with retinopathy | Diabetes | Read |
| C10FB | Type II diabetes mellitus with polyneuropathy | Diabetes | Read |
| C1097 | Non-insulin dependent diabetes mellitus - poor control | Diabetes | Read |
| C1096 | Non-insulin-dependent diabetes mellitus with retinopathy | Diabetes | Read |
| C10FK | Hyperosmolar non-ketotic state in type II diabetes mellitus | Diabetes | Read |
| C1092 | Non-insulin-dependent diabetes mellitus with neuro comps | Diabetes | Read |
| C10FG | Type II diabetes mellitus with arthropathy | Diabetes | Read |
| C10F. | Type 2 diabetes mellitus | Diabetes | Read |
| C109. | Non-insulin dependent diabetes mellitus | Diabetes | Read |
| C10P1 | Type II diabetes mellitus in remission | Diabetes | Read |
| C10F2 | Type 2 diabetes mellitus with neurological complications | Diabetes | Read |
| C109G |  | Diabetes | Read |
| C109G | Type 2 diabetes mellitus with arthropathy | Diabetes | Read |
| C109C | Type II diabetes mellitus with nephropathy | Diabetes | Read |
| C109C | Type 2 diabetes mellitus with nephropathy | Diabetes | Read |
| C10F6 | Type 2 diabetes mellitus with retinopathy | Diabetes | Read |
| C1001 | Diabetes mellitus, adult onset, no mention of complication | Diabetes | Read |
| C10FB | Type 2 diabetes mellitus with polyneuropathy | Diabetes | Read |
| C1097 | Type II diabetes mellitus - poor control | Diabetes | Read |
| C1097 | Type 2 diabetes mellitus - poor control | Diabetes | Read |
| 14AC. | H/O: pulmonary embolus | Embolism | Read |
| 7A093 | Open embolectomy of pulmonary artery | Embolism | Read |
| 7A093 | Trendelenburg pulmonary embolectomy | Embolism | Read |
| 7A0A1 | Percutaneous transluminal embolectomy of pulmonary artery | Embolism | Read |
| 7A0B0 | Pulmonary thromboendarterectomy | Embolism | Read |
| G4010 | Post operative pulmonary embolus | Embolism | Read |
| G4011 | Recurrent pulmonary embolism | Embolism | Read |
| G401. | Pulmonary embolism | Embolism | Read |
| G401. | Pulmonary embolus | Embolism | Read |
| ZV129 | [V] Personal history of pulmonary embolism | Embolism | Read |
| 14A81 | H/O: Deep Vein Thrombosis | Embolism | Read |
| 8CMWA | On deep vein thrombosis care pathway | Embolism | Read |
| 9kg0. | DVT stage 1 service level - enhanced services administration | Embolism | Read |
| 9kg.. | Deep vein thrombosis - enhanced services administration | Embolism | Read |
| 9kg0. | DVT enhanced services administration stage 1 service level | Embolism | Read |
| 9kg1. | DVT stage 2 service level - enhanced services administration | Embolism | Read |
| 9kg2. | DVT stage 3 service level - enhanced services administration | Embolism | Read |
| F0500 | Embolism cavernous sinus | Embolism | Read |
| F050. | Embolism of central nervous system venous sinus | Embolism | Read |
| F05.. | Phlebitis and thrombophlebitis of intracranial sinuses | Embolism | Read |
| F0501 | Embolism superior longitudinal sinus | Embolism | Read |
| F0503 | Embolism transverse sinus | Embolism | Read |
| F0510 | Thrombosis cavernous sinus | Embolism | Read |
| F051. | Thrombosis of central nervous system venous sinuses | Embolism | Read |
| F0511 | Thrombosis of superior longitudinal sinus | Embolism | Read |
| F0512 | Thrombosis lateral sinus | Embolism | Read |
| F0513 | Thrombosis transverse sinus | Embolism | Read |
| F051z | Thrombosis of central nervous system venous sinus NOS | Embolism | Read |
| F0530 | Thrombophlebitis of cavernous sinus | Embolism | Read |
| F053. | Thrombophlebitis of central nervous system venous sinuses | Embolism | Read |
| F0531 | Thrombophlebitis of superior longitudinal venous sinus | Embolism | Read |
| F05z. | Phlebitis or thrombophlebitis of CNS venous sinus NOS | Embolism | Read |
| G6760 | Cereb infarct due cerebral venous thrombosis, nonpyogenic | Embolism | Read |
| G676. | Nonpyogenic venous sinus thrombosis | Embolism | Read |
| G67A. | Cerebral vein thrombosis | Embolism | Read |
| G801. | Deep vein phlebitis and thrombophlebitis of the leg | Embolism | Read |
| G801. | Deep vein thrombosis | Embolism | Read |
| G801. | Deep vein thrombosis, leg | Embolism | Read |
| G801. | DVT - Deep vein thrombosis | Embolism | Read |
| G8016 | Thrombophlebitis of the femoral vein | Embolism | Read |
| G8017 | Thrombophlebitis of the popliteal vein | Embolism | Read |
| G8018 | Thrombophlebitis of the anterior tibial vein | Embolism | Read |
| G8019 | Thrombophlebitis of the dorsalis pedis vein | Embolism | Read |
| G801A | Thrombophlebitis of the posterior tibial vein | Embolism | Read |
| G801B | Deep vein thrombophlebitis of the leg unspecified | Embolism | Read |
| G801C | Deep vein thrombosis of leg related to air travel | Embolism | Read |
| G801D | Deep vein thrombosis of lower limb | Embolism | Read |
| G801E | Deep vein thrombosis of leg related to intravenous drug use | Embolism | Read |
| G801F | Deep vein thrombosis of peroneal vein | Embolism | Read |
| G801G | Recurrent deep vein thrombosis | Embolism | Read |
| G801z | Deep vein phlebitis and thrombophlebitis of the leg NOS | Embolism | Read |
| G80y. | Phlebitis and/or thrombophlebitis of iliac vein | Embolism | Read |
| G80y4 | Thrombophlebitis of the common iliac vein | Embolism | Read |
| G80y5 | Thrombophlebitis of the internal iliac vein | Embolism | Read |
| G80y6 | Thrombophlebitis of the external iliac vein | Embolism | Read |
| G80y7 | Thrombophlebitis of the iliac vein unspecified | Embolism | Read |
| G80y8 | Phlebitis and thrombophlebitis of the iliac vein NOS | Embolism | Read |
| G81.. | Portal vein thrombosis | Embolism | Read |
| G820. | Budd - Chiari syndrome (hepatic vein thrombosis) | Embolism | Read |
| G820. | Hepatic vein thrombosis | Embolism | Read |
| G8220 | Thrombosis of inferior vena cava | Embolism | Read |
| G822. | Embolism and thrombosis of the vena cava | Embolism | Read |
| G823. | Embolism and thrombosis of the renal vein | Embolism | Read |
| G824. | Axillary vein thrombosis | Embolism | Read |
| G825. | Thrombosis of subclavian vein | Embolism | Read |
| G826. | Thrombosis of internal jugular vein | Embolism | Read |
| G827. | Thrombosis of external jugular vein | Embolism | Read |
| Gyu80 | [X]Phlebitis+thrombophlebitis/oth deep vessls/low extremites | Embolism | Read |
| J4202 | Thrombus of the superior mesenteric veins | Embolism | Read |
| SP122 | Post operative deep vein thrombosis | Embolism | Read |
| ZV128 | [V] Personal history deep vein thrombosis | Embolism | Read |
| ZV128 | [V] Personal history DVT- deep vein thrombosis | Embolism | Read |
| I26. | Diagnosis of Pulmonary embolism | Embolism | ICD-10 |
| 8HlL. | Referral for chronic fatigue syndrome activity management | Fatigue | Read |
| 8Q1.. | Activity management for chronic fatigue syndrome | Fatigue | Read |
| E205. | Neurasthenia - nervous debility | Fatigue | Read |
| Eu460 | [X]Neurasthenia | Fatigue | Read |
| F03y. | Myalgic encephalomyelitis | Fatigue | Read |
| F286. | Chronic fatigue syndrome | Fatigue | Read |
| F2860 | Mild chronic fatigue syndrome | Fatigue | Read |
| F2861 | Moderate chronic fatigue syndrome | Fatigue | Read |
| F2862 | Severe chronic fatigue syndrome | Fatigue | Read |
| N239. | Fibromyalgia | Fatigue | Read |
| N2401 | Fibrositis unspecified | Fatigue | Read |
| N2405 | Fibrositis of neck | Fatigue | Read |
| N2406 | Fibrositis arm | Fatigue | Read |
| N248. | Fibromyalgia | Fatigue | Read |
| R0074 | [D]Postviral (asthenic) syndrome | Fatigue | Read |
| R0000 | Drowsiness | Fatigue | Read |
| R0001 | Somnolence | Fatigue | Read |
| R0070 | Malaise | Fatigue | Read |
| R0071 | Fatigue | Fatigue | Read |
| R0072 | Asthenia | Fatigue | Read |
| R0073 | Lethargy | Fatigue | Read |
| R0074 | Postviral (asthenic) syndrome | Fatigue | Read |
| R0075 | Tiredness | Fatigue | Read |
| R007z | Malaise & Fatigue NOS | Fatigue | Read |
| R009. | Confusion | Fatigue | Read |
| R00z7 | Demoralisation and apathy | Fatigue | Read |
| R00zX | Disorientation, unspecified | Fatigue | Read |
| F286. | Chronic Fatigue Syndrome/Postviral fatigue syndrome | Fatigue | Read |
| F2860 | Mild chronic fatigue syndrome | Fatigue | Read |
| F2861 | Moderate chronic fatigue syndrome | Fatigue | Read |
| F2862 | Severe chronic fatigue syndrome | Fatigue | Read |
| Eu460 | Fatigue syndrome | Fatigue | Read |
| 168.. | Fatigue - symptom | Fatigue | Read |
| 1682. | Fatigue | Fatigue | Read |
| 1683. | Tired all the time | Fatigue | Read |
| 1684. | Malaise/lethargy | Fatigue | Read |
| 1686. | Heavy legs | Fatigue | Read |
| 1687. | Heavy feeling | Fatigue | Read |
| 1688. | Exhaustion | Fatigue | Read |
| 168Z. | Tiredness symptom NOS | Fatigue | Read |
| E030. | Acute confusional state | Fatigue | Read |
| E0301 | Acute confusional state, of infective origin | Fatigue | Read |
| E030z | Acute confusional state NOS | Fatigue | Read |
| E031. | Subacute confusional state | Fatigue | Read |
| E0311 | Subacute confusional state, of infective origin | Fatigue | Read |
| E031z | Subacute confusional state NOS | Fatigue | Read |
| 1BW.. | Poor concentration | Fatigue | Read |
| 1BR.. | Reduced concentration | Fatigue | Read |
| 1BR0. | Reduced concentration span | Fatigue | Read |
| E030. | Acute confusional state | Fatigue | Read |
| E0301 | Acute confusional state, of infective origin | Fatigue | Read |
| E030z | Acute confusional state NOS | Fatigue | Read |
| E031. | Subacute confusional state | Fatigue | Read |
| E0311 | Subacute confusional state, of infective origin | Fatigue | Read |
| E031z | Subacute confusional state NOS | Fatigue | Read |
| 1BW.. | Poor concentration | Fatigue | Read |
| 1BR.. | Reduced concentration | Fatigue | Read |
| 1BR0. | Reduced concentration span | Fatigue | Read |
| F286. | CFS - Chronic fatigue syndrome | Fatigue | Read |
| F286. | Chronic fatigue syndrome | Fatigue | Read |
| F2860 | Mild chronic fatigue syndrome | Fatigue | Read |
| F2861 | Moderate chronic fatigue synd | Fatigue | Read |
| F286. | Myalgic encephalomyelitis | Fatigue | Read |
| F2862 | Severe chronic fatigue synd | Fatigue | Read |
| F03y. | Myalgic encephalomyelitis | Fatigue | Read |
| F286. | ME - Myalgic encephalomyelitis | Fatigue | Read |
| 6A61. | Attention deficit hyperactivity disorder annual review | Mental and behavioural disorder | Read |
| 8BPT. | Drug therapy ADHD (attention deficit hyperactivity disorder) | Mental and behavioural disorder | Read |
| 9Ngp. | On drug ther ADHD (attention deficit hyperactivity disorder) | Mental and behavioural disorder | Read |
| E2E01 | Attention deficit with hyperactivity | Mental and behavioural disorder | Read |
| E2E1. | Hyperkinesis with developmental delay | Mental and behavioural disorder | Read |
| E2E2. | Hyperkinetic conduct disorder | Mental and behavioural disorder | Read |
| E2E.. | Childhood hyperkinetic syndrome | Mental and behavioural disorder | Read |
| E2E.. | Overactive child syndrome | Mental and behavioural disorder | Read |
| E2Ey. | Other hyperkinetic manifestation | Mental and behavioural disorder | Read |
| E2Ez. | Hyperkinetic syndrome NOS | Mental and behavioural disorder | Read |
| Eu844 | [X]Overactive disorder assoc mental retard/stereotype movts | Mental and behavioural disorder | Read |
| Eu900 | [X]Disturbance of activity and attention | Mental and behavioural disorder | Read |
| Eu900 | [X]Attention deficit hyperactivity disorder | Mental and behavioural disorder | Read |
| Eu901 | [X]Hyperkinetic conduct disorder | Mental and behavioural disorder | Read |
| Eu901 | [X]Hyperkinetic disorder associated with conduct disorder | Mental and behavioural disorder | Read |
| Eu90. | [X]Hyperkinetic disorders | Mental and behavioural disorder | Read |
| Eu90y | [X]Other hyperkinetic disorders | Mental and behavioural disorder | Read |
| Eu90z | [X]Hyperkinetic disorder, unspecified | Mental and behavioural disorder | Read |
| Eu90z | [X]Hyperkinetic reaction of childhood or adolescence NOS | Mental and behavioural disorder | Read |
| Eu90z | [X]Hyperkinetic syndrome NOS | Mental and behavioural disorder | Read |
| 1466. | H/O: anxiety state | Mental and behavioural disorder | Read |
| 146G. | H/O: agoraphobia | Mental and behavioural disorder | Read |
| 1B1H. | Fear | Mental and behavioural disorder | Read |
| 1B1V. | C/O - panic attack | Mental and behavioural disorder | Read |
| 1Bb.. | Specific fear | Mental and behavioural disorder | Read |
| 225J. | O/E - panic attack | Mental and behavioural disorder | Read |
| 225K. | O/E - fearful mood | Mental and behavioural disorder | Read |
| 285.. | Neurotic condition, insight present | Mental and behavioural disorder | Read |
| 286.. | Poor insight into neurotic condition | Mental and behavioural disorder | Read |
| 8G52. | Antiphobic therapy | Mental and behavioural disorder | Read |
| 8G94. | Anxiety management training | Mental and behavioural disorder | Read |
| 8HHp. | Referral for guided self-help for anxiety | Mental and behavioural disorder | Read |
| 9N54. | Encounter for fear | Mental and behavioural disorder | Read |
| E2000 | Anxiety state unspecified | Mental and behavioural disorder | Read |
| E2001 | Panic disorder | Mental and behavioural disorder | Read |
| E2001 | Panic attack | Mental and behavioural disorder | Read |
| E2002 | Generalised anxiety disorder | Mental and behavioural disorder | Read |
| E2003 | Anxiety with depression | Mental and behavioural disorder | Read |
| E2004 | Chronic anxiety | Mental and behavioural disorder | Read |
| E2005 | Recurrent anxiety | Mental and behavioural disorder | Read |
| E200. | Anxiety states | Mental and behavioural disorder | Read |
| E200z | Anxiety state NOS | Mental and behavioural disorder | Read |
| E201B | Compensation neurosis | Mental and behavioural disorder | Read |
| E2020 | Phobia unspecified | Mental and behavioural disorder | Read |
| E2021 | Agoraphobia with panic attacks | Mental and behavioural disorder | Read |
| E2022 | Agoraphobia without mention of panic attacks | Mental and behavioural disorder | Read |
| E2023 | Social phobia, fear of eating in public | Mental and behavioural disorder | Read |
| E2024 | Social phobia, fear of public speaking | Mental and behavioural disorder | Read |
| E2025 | Social phobia, fear of public washing | Mental and behavioural disorder | Read |
| E2026 | Acrophobia | Mental and behavioural disorder | Read |
| E2027 | Animal phobia | Mental and behavioural disorder | Read |
| E2028 | Claustrophobia | Mental and behavioural disorder | Read |
| E2029 | Fear of crowds | Mental and behavioural disorder | Read |
| E202A | Fear of flying | Mental and behavioural disorder | Read |
| E202B | Cancer phobia | Mental and behavioural disorder | Read |
| E202C | Dental phobia | Mental and behavioural disorder | Read |
| E202D | Fear of death | Mental and behavioural disorder | Read |
| E202. | Phobic disorders | Mental and behavioural disorder | Read |
| E202. | Social phobic disorders | Mental and behavioural disorder | Read |
| E202. | Phobic anxiety | Mental and behavioural disorder | Read |
| E202E | Fear of pregnancy | Mental and behavioural disorder | Read |
| E202z | Phobic disorder NOS | Mental and behavioural disorder | Read |
| E20.. | Neurotic disorders | Mental and behavioural disorder | Read |
| E20y1 | Writer's cramp neurosis | Mental and behavioural disorder | Read |
| E20y2 | Other occupational neurosis | Mental and behavioural disorder | Read |
| E20y3 | Psychasthenic neurosis | Mental and behavioural disorder | Read |
| E20y. | Other neurotic disorders | Mental and behavioural disorder | Read |
| E20yz | Other neurotic disorder NOS | Mental and behavioural disorder | Read |
| E20z. | Neurotic disorder NOS | Mental and behavioural disorder | Read |
| Eu341 | [X]Depressive neurosis | Mental and behavioural disorder | Read |
| Eu341 | [X]Neurotic depression | Mental and behavioural disorder | Read |
| Eu341 | [X]Persistant anxiety depression | Mental and behavioural disorder | Read |
| Eu400 | [X]Agoraphobia | Mental and behavioural disorder | Read |
| Eu400 | [X]Agoraphobia without history of panic disorder | Mental and behavioural disorder | Read |
| Eu400 | [X]Panic disorder with agoraphobia | Mental and behavioural disorder | Read |
| Eu401 | [X]Social phobias | Mental and behavioural disorder | Read |
| Eu401 | [X]Social neurosis | Mental and behavioural disorder | Read |
| Eu402 | [X]Specific (isolated) phobias | Mental and behavioural disorder | Read |
| Eu402 | [X]Acrophobia | Mental and behavioural disorder | Read |
| Eu402 | [X]Animal phobias | Mental and behavioural disorder | Read |
| Eu402 | [X]Claustrophobia | Mental and behavioural disorder | Read |
| Eu402 | [X]Simple phobia | Mental and behavioural disorder | Read |
| Eu403 | [X]Needle phobia | Mental and behavioural disorder | Read |
| Eu40. | [X]Phobic anxiety disorders | Mental and behavioural disorder | Read |
| Eu40y | [X]Other phobic anxiety disorders | Mental and behavioural disorder | Read |
| Eu40z | [X]Phobic anxiety disorder, unspecified | Mental and behavioural disorder | Read |
| Eu40z | [X]Phobia NOS | Mental and behavioural disorder | Read |
| Eu40z | [X]Phobic state NOS | Mental and behavioural disorder | Read |
| Eu410 | [X]Panic disorder [episodic paroxysmal anxiety] | Mental and behavioural disorder | Read |
| Eu410 | [X]Panic attack | Mental and behavioural disorder | Read |
| Eu410 | [X]Panic state | Mental and behavioural disorder | Read |
| Eu411 | [X]Generalized anxiety disorder | Mental and behavioural disorder | Read |
| Eu411 | [X]Anxiety neurosis | Mental and behavioural disorder | Read |
| Eu411 | [X]Anxiety reaction | Mental and behavioural disorder | Read |
| Eu411 | [X]Anxiety state | Mental and behavioural disorder | Read |
| Eu412 | [X]Mixed anxiety and depressive disorder | Mental and behavioural disorder | Read |
| Eu412 | [X]Mild anxiety depression | Mental and behavioural disorder | Read |
| Eu413 | [X]Other mixed anxiety disorders | Mental and behavioural disorder | Read |
| Eu41. | [X]Other anxiety disorders | Mental and behavioural disorder | Read |
| Eu41y | [X]Other specified anxiety disorders | Mental and behavioural disorder | Read |
| Eu41y | [X]Anxiety hysteria | Mental and behavioural disorder | Read |
| Eu41z | [X]Anxiety disorder, unspecified | Mental and behavioural disorder | Read |
| Eu41z | [X]Anxiety NOS | Mental and behavioural disorder | Read |
| Z481. | Phobia counselling | Mental and behavioural disorder | Read |
| Z4L1. | Anxiety counselling | Mental and behavioural disorder | Read |
| ZV112 | [V]Personal history of neurosis | Mental and behavioural disorder | Read |
| E1400 | Active infantile autism | Mental and behavioural disorder | Read |
| E1401 | Residual infantile autism | Mental and behavioural disorder | Read |
| E140. | Infantile autism | Mental and behavioural disorder | Read |
| E140. | Kanner's syndrome | Mental and behavioural disorder | Read |
| E140. | Autism | Mental and behavioural disorder | Read |
| E140. | Childhood autism | Mental and behavioural disorder | Read |
| E140z | Infantile autism NOS | Mental and behavioural disorder | Read |
| Eu840 | [X]Childhood autism | Mental and behavioural disorder | Read |
| Eu840 | [X]Autistic disorder | Mental and behavioural disorder | Read |
| Eu840 | [X]Infantile autism | Mental and behavioural disorder | Read |
| Eu840 | [X]Kanner's syndrome | Mental and behavioural disorder | Read |
| Eu841 | [X]Atypical autism | Mental and behavioural disorder | Read |
| Eu841 | [X]Mental retardation with autistic features | Mental and behavioural disorder | Read |
| Eu845 | [X]Asperger's syndrome | Mental and behavioural disorder | Read |
| Eu845 | [X]Autistic psychopathy | Mental and behavioural disorder | Read |
| Eu84z | [X]Autistic spectrum disorder | Mental and behavioural disorder | Read |
| 1465. | H/O: depression | Mental and behavioural disorder | Read |
| 212S. | Depression resolved | Mental and behavioural disorder | Read |
| 8BK0. | Depression management programme | Mental and behavioural disorder | Read |
| 8CAa. | Patient given advice about management of depression | Mental and behavioural disorder | Read |
| 8HHq. | Referral for guided self-help for depression | Mental and behavioural disorder | Read |
| 9H90. | Depression annual review | Mental and behavioural disorder | Read |
| 9H91. | Depression medication review | Mental and behavioural disorder | Read |
| 9H92. | Depression interim review | Mental and behavioural disorder | Read |
| 9HA0. | On depression register | Mental and behavioural disorder | Read |
| 9k40. | Depression - enhanced service completed | Mental and behavioural disorder | Read |
| 9k4.. | Depression - enhanced services administration | Mental and behavioural disorder | Read |
| 9kQ.. | On full dose long term treatment depression - enh serv admin | Mental and behavioural disorder | Read |
| 9Ov0. | Depression monitoring first letter | Mental and behavioural disorder | Read |
| 9Ov1. | Depression monitoring second letter | Mental and behavioural disorder | Read |
| 9Ov2. | Depression monitoring third letter | Mental and behavioural disorder | Read |
| 9Ov3. | Depression monitoring verbal invite | Mental and behavioural disorder | Read |
| 9Ov4. | Depression monitoring telephone invite | Mental and behavioural disorder | Read |
| 9Ov.. | Depression monitoring administration | Mental and behavioural disorder | Read |
| E0013 | Presenile dementia with depression | Mental and behavioural disorder | Read |
| E0021 | Senile dementia with depression | Mental and behavioural disorder | Read |
| E0043 | Arteriosclerotic dementia with depression | Mental and behavioural disorder | Read |
| E1120 | Single major depressive episode, unspecified | Mental and behavioural disorder | Read |
| E1121 | Single major depressive episode, mild | Mental and behavioural disorder | Read |
| E1122 | Single major depressive episode, moderate | Mental and behavioural disorder | Read |
| E1123 | Single major depressive episode, severe, without psychosis | Mental and behavioural disorder | Read |
| E1124 | Single major depressive episode, severe, with psychosis | Mental and behavioural disorder | Read |
| E1125 | Single major depressive episode, partial or unspec remission | Mental and behavioural disorder | Read |
| E1126 | Single major depressive episode, in full remission | Mental and behavioural disorder | Read |
| E112. | Single major depressive episode | Mental and behavioural disorder | Read |
| E112. | Agitated depression | Mental and behavioural disorder | Read |
| E112. | Endogenous depression first episode | Mental and behavioural disorder | Read |
| E112. | Endogenous depression first episode | Mental and behavioural disorder | Read |
| E112. | Endogenous depression | Mental and behavioural disorder | Read |
| E112z | Single major depressive episode NOS | Mental and behavioural disorder | Read |
| E1130 | Recurrent major depressive episodes, unspecified | Mental and behavioural disorder | Read |
| E1131 | Recurrent major depressive episodes, mild | Mental and behavioural disorder | Read |
| E1132 | Recurrent major depressive episodes, moderate | Mental and behavioural disorder | Read |
| E1133 | Recurrent major depressive episodes, severe, no psychosis | Mental and behavioural disorder | Read |
| E1134 | Recurrent major depressive episodes, severe, with psychosis | Mental and behavioural disorder | Read |
| E1135 | Recurrent major depressive episodes,partial/unspec remission | Mental and behavioural disorder | Read |
| E1136 | Recurrent major depressive episodes, in full remission | Mental and behavioural disorder | Read |
| E1137 | Recurrent depression | Mental and behavioural disorder | Read |
| E113. | Recurrent major depressive episode | Mental and behavioural disorder | Read |
| E113. | Endogenous depression - recurrent | Mental and behavioural disorder | Read |
| E113z | Recurrent major depressive episode NOS | Mental and behavioural disorder | Read |
| E118. | Seasonal affective disorder | Mental and behavioural disorder | Read |
| E11.. | Depressive psychoses | Mental and behavioural disorder | Read |
| E11y2 | Atypical depressive disorder | Mental and behavioural disorder | Read |
| E11z2 | Masked depression | Mental and behavioural disorder | Read |
| E130. | Reactive depressive psychosis | Mental and behavioural disorder | Read |
| E130. | Psychotic reactive depression | Mental and behavioural disorder | Read |
| E135. | Agitated depression | Mental and behavioural disorder | Read |
| E2003 | Anxiety with depression | Mental and behavioural disorder | Read |
| E291. | Prolonged depressive reaction | Mental and behavioural disorder | Read |
| E2B1. | Chronic depression | Mental and behavioural disorder | Read |
| E2B.. | Depressive disorder NEC | Mental and behavioural disorder | Read |
| Eu204 | [X]Post-schizophrenic depression | Mental and behavioural disorder | Read |
| Eu251 | [X]Schizoaffective disorder, depressive type | Mental and behavioural disorder | Read |
| Eu251 | [X]Schizoaffective psychosis, depressive type | Mental and behavioural disorder | Read |
| Eu251 | [X]Schizophreniform psychosis, depressive type | Mental and behavioural disorder | Read |
| Eu320 | [X]Mild depressive episode | Mental and behavioural disorder | Read |
| Eu321 | [X]Moderate depressive episode | Mental and behavioural disorder | Read |
| Eu322 | [X]Severe depressive episode without psychotic symptoms | Mental and behavioural disorder | Read |
| Eu322 | [X]Single episode agitated depressn w'out psychotic symptoms | Mental and behavioural disorder | Read |
| Eu322 | [X]Single episode major depression w'out psychotic symptoms | Mental and behavioural disorder | Read |
| Eu322 | [X]Single episode vital depression w'out psychotic symptoms | Mental and behavioural disorder | Read |
| Eu323 | [X]Severe depressive episode with psychotic symptoms | Mental and behavioural disorder | Read |
| Eu323 | [X]Single episode of major depression and psychotic symptoms | Mental and behavioural disorder | Read |
| Eu323 | [X]Single episode of psychogenic depressive psychosis | Mental and behavioural disorder | Read |
| Eu323 | [X]Single episode of psychotic depression | Mental and behavioural disorder | Read |
| Eu323 | [X]Single episode of reactive depressive psychosis | Mental and behavioural disorder | Read |
| Eu324 | [X]Mild depression | Mental and behavioural disorder | Read |
| Eu325 | [X]Major depression, mild | Mental and behavioural disorder | Read |
| Eu326 | [X]Major depression, moderately severe | Mental and behavioural disorder | Read |
| Eu327 | [X]Major depression, severe without psychotic symptoms | Mental and behavioural disorder | Read |
| Eu328 | [X]Major depression, severe with psychotic symptoms | Mental and behavioural disorder | Read |
| Eu329 | [X]Single major depr ep, severe with psych, psych in remiss | Mental and behavioural disorder | Read |
| Eu32A | [X]Recurr major depr ep, severe with psych, psych in remiss | Mental and behavioural disorder | Read |
| Eu32. | [X]Depressive episode | Mental and behavioural disorder | Read |
| Eu32. | [X]Single episode of depressive reaction | Mental and behavioural disorder | Read |
| Eu32. | [X]Single episode of psychogenic depression | Mental and behavioural disorder | Read |
| Eu32. | [X]Single episode of reactive depression | Mental and behavioural disorder | Read |
| Eu32y | [X]Other depressive episodes | Mental and behavioural disorder | Read |
| Eu32y | [X]Atypical depression | Mental and behavioural disorder | Read |
| Eu32y | [X]Single episode of masked depression NOS | Mental and behavioural disorder | Read |
| Eu32z | [X]Depressive episode, unspecified | Mental and behavioural disorder | Read |
| Eu32z | [X]Depression NOS | Mental and behavioural disorder | Read |
| Eu32z | [X]Depressive disorder NOS | Mental and behavioural disorder | Read |
| Eu32z | [X]Prolonged single episode of reactive depression | Mental and behavioural disorder | Read |
| Eu32z | [X] Reactive depression NOS | Mental and behavioural disorder | Read |
| Eu330 | [X]Recurrent depressive disorder, current episode mild | Mental and behavioural disorder | Read |
| Eu331 | [X]Recurrent depressive disorder, current episode moderate | Mental and behavioural disorder | Read |
| Eu332 | [X]Recurr depress disorder cur epi severe without psyc sympt | Mental and behavioural disorder | Read |
| Eu332 | [X]Endogenous depression without psychotic symptoms | Mental and behavioural disorder | Read |
| Eu332 | [X]Major depression, recurrent without psychotic symptoms | Mental and behavioural disorder | Read |
| Eu332 | [X]Manic-depress psychosis,depressd,no psychotic symptoms | Mental and behavioural disorder | Read |
| Eu332 | [X]Vital depression, recurrent without psychotic symptoms | Mental and behavioural disorder | Read |
| Eu333 | [X]Recurrent depress disorder cur epi severe with psyc symp | Mental and behavioural disorder | Read |
| Eu333 | [X]Endogenous depression with psychotic symptoms | Mental and behavioural disorder | Read |
| Eu333 | [X]Manic-depress psychosis,depressed type+psychotic symptoms | Mental and behavioural disorder | Read |
| Eu333 | [X]Recurr severe episodes/major depression+psychotic symptom | Mental and behavioural disorder | Read |
| Eu333 | [X]Recurr severe episodes/psychogenic depressive psychosis | Mental and behavioural disorder | Read |
| Eu333 | [X]Recurrent severe episodes of psychotic depression | Mental and behavioural disorder | Read |
| Eu333 | [X]Recurrent severe episodes/reactive depressive psychosis | Mental and behavioural disorder | Read |
| Eu334 | [X]Recurrent depressive disorder, currently in remission | Mental and behavioural disorder | Read |
| Eu33. | [X]Recurrent depressive disorder | Mental and behavioural disorder | Read |
| Eu33. | [X]Recurrent episodes of depressive reaction | Mental and behavioural disorder | Read |
| Eu33. | [X]Recurrent episodes of psychogenic depression | Mental and behavioural disorder | Read |
| Eu33. | [X]Recurrent episodes of reactive depression | Mental and behavioural disorder | Read |
| Eu33. | [X]Seasonal depressive disorder | Mental and behavioural disorder | Read |
| Eu33. | [X]SAD - Seasonal affective disorder | Mental and behavioural disorder | Read |
| Eu33y | [X]Other recurrent depressive disorders | Mental and behavioural disorder | Read |
| Eu33z | [X]Recurrent depressive disorder, unspecified | Mental and behavioural disorder | Read |
| Eu33z | [X]Monopolar depression NOS | Mental and behavioural disorder | Read |
| Eu341 | [X]Dysthymia | Mental and behavioural disorder | Read |
| Eu341 | [X]Depressive neurosis | Mental and behavioural disorder | Read |
| Eu341 | [X]Depressive personality disorder | Mental and behavioural disorder | Read |
| Eu341 | [X]Neurotic depression | Mental and behavioural disorder | Read |
| Eu341 | [X]Persistant anxiety depression | Mental and behavioural disorder | Read |
| Eu412 | [X]Mixed anxiety and depressive disorder | Mental and behavioural disorder | Read |
| Eu412 | [X]Mild anxiety depression | Mental and behavioural disorder | Read |
| E2030 | Compulsive neurosis | Mental and behavioural disorder | Read |
| E2031 | Obsessional neurosis | Mental and behavioural disorder | Read |
| E203. | Obsessive-compulsive disorders | Mental and behavioural disorder | Read |
| E203. | Anancastic neurosis | Mental and behavioural disorder | Read |
| E203z | Obsessive-compulsive disorder NOS | Mental and behavioural disorder | Read |
| Eu420 | [X]Predominantly obsessional thoughts or ruminations | Mental and behavioural disorder | Read |
| Eu421 | [X]Predominantly compulsive acts [obsessional rituals] | Mental and behavioural disorder | Read |
| Eu422 | [X]Mixed obsessional thoughts and acts | Mental and behavioural disorder | Read |
| Eu42. | [X]Obsessive - compulsive disorder | Mental and behavioural disorder | Read |
| Eu42. | [X]Anankastic neurosis | Mental and behavioural disorder | Read |
| Eu42. | [X]Obsessive-compulsive neurosis | Mental and behavioural disorder | Read |
| Eu42y | [X]Other obsessive-compulsive disorders | Mental and behavioural disorder | Read |
| Eu42z | [X]Obsessive-compulsive disorder, unspecified | Mental and behavioural disorder | Read |
| 1464. | H/O: schizophrenia | Mental and behavioural disorder | Read |
| 146H. | H/O: psychosis | Mental and behavioural disorder | Read |
| 212W. | Schizophrenia resolved | Mental and behavioural disorder | Read |
| 212X. | Psychosis resolved | Mental and behavioural disorder | Read |
| 285.. | Psychotic condition, insight present | Mental and behavioural disorder | Read |
| 286.. | Poor insight into psychotic condition | Mental and behavioural disorder | Read |
| E1000 | Unspecified schizophrenia | Mental and behavioural disorder | Read |
| E1001 | Subchronic schizophrenia | Mental and behavioural disorder | Read |
| E1002 | Chronic schizophrenic | Mental and behavioural disorder | Read |
| E1003 | Acute exacerbation of subchronic schizophrenia | Mental and behavioural disorder | Read |
| E1004 | Acute exacerbation of chronic schizophrenia | Mental and behavioural disorder | Read |
| E1005 | Schizophrenia in remission | Mental and behavioural disorder | Read |
| E100. | Simple schizophrenia | Mental and behavioural disorder | Read |
| E100. | Schizophrenia simplex | Mental and behavioural disorder | Read |
| E100z | Simple schizophrenia NOS | Mental and behavioural disorder | Read |
| E1010 | Unspecified hebephrenic schizophrenia | Mental and behavioural disorder | Read |
| E1014 | Acute exacerbation of chronic hebephrenic schizophrenia | Mental and behavioural disorder | Read |
| E1015 | Hebephrenic schizophrenia in remission | Mental and behavioural disorder | Read |
| E101. | Hebephrenic schizophrenia | Mental and behavioural disorder | Read |
| E101z | Hebephrenic schizophrenia NOS | Mental and behavioural disorder | Read |
| E1020 | Unspecified catatonic schizophrenia | Mental and behavioural disorder | Read |
| E1021 | Subchronic catatonic schizophrenia | Mental and behavioural disorder | Read |
| E1024 | Acute exacerbation of chronic catatonic schizophrenia | Mental and behavioural disorder | Read |
| E1025 | Catatonic schizophrenia in remission | Mental and behavioural disorder | Read |
| E102. | Catatonic schizophrenia | Mental and behavioural disorder | Read |
| E102z | Catatonic schizophrenia NOS | Mental and behavioural disorder | Read |
| E1030 | Unspecified paranoid schizophrenia | Mental and behavioural disorder | Read |
| E1031 | Subchronic paranoid schizophrenia | Mental and behavioural disorder | Read |
| E1032 | Chronic paranoid schizophrenia | Mental and behavioural disorder | Read |
| E1033 | Acute exacerbation of subchronic paranoid schizophrenia | Mental and behavioural disorder | Read |
| E1034 | Acute exacerbation of chronic paranoid schizophrenia | Mental and behavioural disorder | Read |
| E1035 | Paranoid schizophrenia in remission | Mental and behavioural disorder | Read |
| E103. | Paranoid schizophrenia | Mental and behavioural disorder | Read |
| E103z | Paranoid schizophrenia NOS | Mental and behavioural disorder | Read |
| E1050 | Unspecified latent schizophrenia | Mental and behavioural disorder | Read |
| E1052 | Chronic latent schizophrenia | Mental and behavioural disorder | Read |
| E1055 | Latent schizophrenia in remission | Mental and behavioural disorder | Read |
| E105. | Latent schizophrenia | Mental and behavioural disorder | Read |
| E105z | Latent schizophrenia NOS | Mental and behavioural disorder | Read |
| E106. | Residual schizophrenia | Mental and behavioural disorder | Read |
| E1070 | Unspecified schizo-affective schizophrenia | Mental and behavioural disorder | Read |
| E1071 | Subchronic schizo-affective schizophrenia | Mental and behavioural disorder | Read |
| E1072 | Chronic schizo-affective schizophrenia | Mental and behavioural disorder | Read |
| E1073 | Acute exacerbation subchronic schizo-affective schizophrenia | Mental and behavioural disorder | Read |
| E1074 | Acute exacerbation of chronic schizo-affective schizophrenia | Mental and behavioural disorder | Read |
| E1075 | Schizo-affective schizophrenia in remission | Mental and behavioural disorder | Read |
| E107. | Schizo-affective schizophrenia | Mental and behavioural disorder | Read |
| E107. | Cyclic schizophrenia | Mental and behavioural disorder | Read |
| E107z | Schizo-affective schizophrenia NOS | Mental and behavioural disorder | Read |
| E10.. | Schizophrenic disorders | Mental and behavioural disorder | Read |
| E10y0 | Atypical schizophrenia | Mental and behavioural disorder | Read |
| E10y1 | Coenesthopathic schizophrenia | Mental and behavioural disorder | Read |
| E10y. | Other schizophrenia | Mental and behavioural disorder | Read |
| E10y. | Cenesthopathic schizophrenia | Mental and behavioural disorder | Read |
| E10yz | Other schizophrenia NOS | Mental and behavioural disorder | Read |
| E10z. | Schizophrenia NOS | Mental and behavioural disorder | Read |
| E120. | Simple paranoid state | Mental and behavioural disorder | Read |
| E121. | Chronic paranoid psychosis | Mental and behavioural disorder | Read |
| E121. | Sander's disease | Mental and behavioural disorder | Read |
| E122. | Paraphrenia | Mental and behavioural disorder | Read |
| E12.. | Paranoid states | Mental and behavioural disorder | Read |
| E12y0 | Paranoia querulans | Mental and behavioural disorder | Read |
| E12y. | Other paranoid states | Mental and behavioural disorder | Read |
| E12yz | Other paranoid states NOS | Mental and behavioural disorder | Read |
| E12z. | Paranoid psychosis NOS | Mental and behavioural disorder | Read |
| E1... | Non-organic psychoses | Mental and behavioural disorder | Read |
| E1y.. | Other specified non-organic psychoses | Mental and behavioural disorder | Read |
| E1z.. | Non-organic psychosis NOS | Mental and behavioural disorder | Read |
| Eu200 | [X]Paranoid schizophrenia | Mental and behavioural disorder | Read |
| Eu200 | [X]Paraphrenic schizophrenia | Mental and behavioural disorder | Read |
| Eu201 | [X]Hebephrenic schizophrenia | Mental and behavioural disorder | Read |
| Eu201 | [X]Disorganised schizophrenia | Mental and behavioural disorder | Read |
| Eu202 | [X]Catatonic schizophrenia | Mental and behavioural disorder | Read |
| Eu202 | [X]Catatonic stupor | Mental and behavioural disorder | Read |
| Eu202 | [X]Schizophrenic catalepsy | Mental and behavioural disorder | Read |
| Eu202 | [X]Schizophrenic catatonia | Mental and behavioural disorder | Read |
| Eu202 | [X]Schizophrenic flexibilatis cerea | Mental and behavioural disorder | Read |
| Eu203 | [X]Undifferentiated schizophrenia | Mental and behavioural disorder | Read |
| Eu203 | [X]Atypical schizophrenia | Mental and behavioural disorder | Read |
| Eu204 | [X]Post-schizophrenic depression | Mental and behavioural disorder | Read |
| Eu205 | [X]Residual schizophrenia | Mental and behavioural disorder | Read |
| Eu205 | [X]Chronic undifferentiated schizophrenia | Mental and behavioural disorder | Read |
| Eu206 | [X]Simple schizophrenia | Mental and behavioural disorder | Read |
| Eu20. | [X]Schizophrenia | Mental and behavioural disorder | Read |
| Eu20y | [X]Other schizophrenia | Mental and behavioural disorder | Read |
| Eu20y | [X]Schizophreniform disord NOS | Mental and behavioural disorder | Read |
| Eu20y | [X]Schizophrenifrm psychos NOS | Mental and behavioural disorder | Read |
| Eu20z | [X]Schizophrenia, unspecified | Mental and behavioural disorder | Read |
| Eu21. | [X]Schizotypal disorder | Mental and behavioural disorder | Read |
| Eu21. | [X]Latent schizophrenic reaction | Mental and behavioural disorder | Read |
| Eu21. | [X]Borderline schizophrenia | Mental and behavioural disorder | Read |
| Eu21. | [X]Latent schizophrenia | Mental and behavioural disorder | Read |
| Eu21. | [X]Prepsychotic schizophrenia | Mental and behavioural disorder | Read |
| Eu21. | [X]Prodromal schizophrenia | Mental and behavioural disorder | Read |
| Eu21. | [X]Pseudoneurotic schizophrenia | Mental and behavioural disorder | Read |
| Eu21. | [X]Pseudopsychopathic schizophrenia | Mental and behavioural disorder | Read |
| Eu220 | [X]Delusional disorder | Mental and behavioural disorder | Read |
| Eu220 | [X]Paranoid psychosis | Mental and behavioural disorder | Read |
| Eu220 | [X]Paranoid state | Mental and behavioural disorder | Read |
| Eu220 | [X]Paraphrenia - late | Mental and behavioural disorder | Read |
| Eu220 | [X]Sensitiver Beziehungswahn | Mental and behavioural disorder | Read |
| Eu220 | [X]Paranoia | Mental and behavioural disorder | Read |
| Eu221 | [X]Delusional misidentification syndrome | Mental and behavioural disorder | Read |
| Eu221 | [X]Capgras syndrome | Mental and behavioural disorder | Read |
| Eu222 | [X]Cotard syndrome | Mental and behavioural disorder | Read |
| Eu223 | [X]Paranoid state in remission | Mental and behavioural disorder | Read |
| Eu22. | [X]Persistent delusional disorders | Mental and behavioural disorder | Read |
| Eu22y | [X]Other persistent delusional disorders | Mental and behavioural disorder | Read |
| Eu22y | [X]Delusional dysmorphophobia | Mental and behavioural disorder | Read |
| Eu22y | [X]Involutional paranoid state | Mental and behavioural disorder | Read |
| Eu22y | [X]Paranoia querulans | Mental and behavioural disorder | Read |
| Eu22z | [X]Persistent delusional disorder, unspecified | Mental and behavioural disorder | Read |
| Eu250 | [X]Schizoaffective disorder, manic type | Mental and behavioural disorder | Read |
| Eu250 | [X]Schizoaffective psychosis, manic type | Mental and behavioural disorder | Read |
| Eu250 | [X]Schizophreniform psychosis, manic type | Mental and behavioural disorder | Read |
| Eu251 | [X]Schizoaffective disorder, depressive type | Mental and behavioural disorder | Read |
| Eu251 | [X]Schizoaffective psychosis, depressive type | Mental and behavioural disorder | Read |
| Eu251 | [X]Schizophreniform psychosis, depressive type | Mental and behavioural disorder | Read |
| Eu252 | [X]Schizoaffective disorder, mixed type | Mental and behavioural disorder | Read |
| Eu252 | [X]Cyclic schizophrenia | Mental and behavioural disorder | Read |
| Eu252 | [X]Mixed schizophrenic and affective psychosis | Mental and behavioural disorder | Read |
| Eu25. | [X]Schizoaffective disorders | Mental and behavioural disorder | Read |
| Eu25y | [X]Other schizoaffective disorders | Mental and behavioural disorder | Read |
| Eu25z | [X]Schizoaffective disorder, unspecified | Mental and behavioural disorder | Read |
| Eu25z | [X]Schizoaffective psychosis NOS | Mental and behavioural disorder | Read |
| Eu26. | [X]Nonorganic psychosis in remission | Mental and behavioural disorder | Read |
| Eu2.. | [X]Schizophrenia, schizotypal and delusional disorders | Mental and behavioural disorder | Read |
| Eu2y. | [X]Other nonorganic psychotic disorders | Mental and behavioural disorder | Read |
| Eu2y. | [X]Chronic hallucinatory psychosis | Mental and behavioural disorder | Read |
| Eu2z. | [X]Unspecified nonorganic psychosis | Mental and behavioural disorder | Read |
| Eu2z. | [X]Psychosis NOS | Mental and behavioural disorder | Read |
| ZV110 | [V]Personal history of schizophrenia | Mental and behavioural disorder | Read |
| F90. | Diagnosis of Hyperkinetic disorders | Mental and behavioural disorder | ICD-10 |
| F40. | Diagnosis of Anxiety disorders | Mental and behavioural disorder | ICD-10 |
| F41. | Diagnosis of Anxiety disorders | Mental and behavioural disorder | ICD-10 |
| F84. | Diagnosis of Autism and Asperger's syndrome | Mental and behavioural disorder | ICD-10 |
| F84. | Diagnosis of Autism and Asperger's syndrome | Mental and behavioural disorder | ICD-10 |
| F84. | Diagnosis of Autism and Asperger's syndrome | Mental and behavioural disorder | ICD-10 |
| F30. | Diagnosis of Bipolar affective disorder and mania | Mental and behavioural disorder | ICD-10 |
| F31. | Diagnosis of Bipolar affective disorder and mania | Mental and behavioural disorder | ICD-10 |
| F32. | Diagnosis of Depression | Mental and behavioural disorder | ICD-10 |
| F33. | Diagnosis of Depression | Mental and behavioural disorder | ICD-10 |
| F42. | Diagnosis of Obsessive-compulsive disorder | Mental and behavioural disorder | ICD-10 |
| F20. | Diagnosis of Schizophrenia, schizotypal and delusional disorders | Mental and behavioural disorder | ICD-10 |
| F21. | Diagnosis of Schizophrenia, schizotypal and delusional disorders | Mental and behavioural disorder | ICD-10 |
| F22. | Diagnosis of Schizophrenia, schizotypal and delusional disorders | Mental and behavioural disorder | ICD-10 |
| F25. | Diagnosis of Schizophrenia, schizotypal and delusional disorders | Mental and behavioural disorder | ICD-10 |
| F28. | Diagnosis of Schizophrenia, schizotypal and delusional disorders | Mental and behavioural disorder | ICD-10 |
| F29. | Diagnosis of Schizophrenia, schizotypal and delusional disorders | Mental and behavioural disorder | ICD-10 |
| U2zy. | [X]Intent self harm by unspecif means occ oth specif place | Mental and behavioural disorder | Read |
| U20By | [X]Int self poison other gas/vapour other spec place | Mental and behavioural disorder | Read |
| U241. |  | Mental and behavioural disorder | Read |
| ZX1Q. | Throwing self in front of train | Mental and behavioural disorder | Read |
| U20B0 | [X]Int self poison/exposure to other gas/vapour at home | Mental and behavioural disorder | Read |
| U206. | [X]Intent self poison/exposure to hallucinogen | Mental and behavioural disorder | Read |
| U200. | [X]Overdose - ibuprofen | Mental and behavioural disorder | Read |
| U200. | [X]Overdose - aspirin | Mental and behavioural disorder | Read |
| TKxz. | Suicide and selfinflicted injury by other means NOS | Mental and behavioural disorder | Read |
| U200. | [X]Overdose - paracetamol | Mental and behavioural disorder | Read |
| U22.. | [X]Intentional self harm by drowning and submersion | Mental and behavioural disorder | Read |
| U20yz | [X]Intent self poison unspecif chemical unspecif place | Mental and behavioural disorder | Read |
| TKx.. | Suicide and selfinflicted injury by other means | Mental and behavioural disorder | Read |
| U208z | [X]Intent self poison oth/unsp drug/medic unspecif place | Mental and behavioural disorder | Read |
| U202y | [X]Int self poison sedative hypnotic other spec place | Mental and behavioural disorder | Read |
| TK3z. | Suicide + selfinflicted inj by hang/strangle/suffocate NOS | Mental and behavioural disorder | Read |
| U212. | [X]Inten slf harm hang strang/suffc sch oth ins/pub adm area | Mental and behavioural disorder | Read |
| U2B0. | [X]Intent self harm by jumping from high place occ at home | Mental and behavioural disorder | Read |
| U20A. | [X]Intentional self poison organ solvent,halogen hydrocarb | Mental and behavioural disorder | Read |
| ZX1K. | Setting fire to self | Mental and behavioural disorder | Read |
| TK11. | Suicide + selfinflicted poisoning by liquified petrol gas | Mental and behavioural disorder | Read |
| ZX1K. | Setting self alight | Mental and behavioural disorder | Read |
| U200. | [X]Intent self poison/exposure to nonopioid analgesic | Mental and behavioural disorder | Read |
| U2D.. | [X]Intentional self harm by crashing of motor vehicle | Mental and behavioural disorder | Read |
| ZX1H1 | Self-strangulation | Mental and behavioural disorder | Read |
| TK05. | Suicide + selfinflicted poisoning by drug or medicine NOS | Mental and behavioural disorder | Read |
| TK3.. | Suicide + selfinflicted injury by hang/strangulate/suffocate | Mental and behavioural disorder | Read |
| U270. | [X]Intention self harm by smoke fire/flames occurrn at home | Mental and behavioural disorder | Read |
| TK010 | Suicide and self inflicted injury by Amylobarbitone | Mental and behavioural disorder | Read |
| U20A. | [X]Self poisoning from glue solvent | Mental and behavioural disorder | Read |
| ZX1K. | Self-incineration | Mental and behavioural disorder | Read |
| U21y. | [X]Intent self harm by hangng strangul/suffoct oth spec plce | Mental and behavioural disorder | Read |
| U2080 | [X]Int self poison/exposure to oth/unsp drug/medicam home | Mental and behavioural disorder | Read |
| U2A.. | [X]Intentional self harm by blunt object | Mental and behavioural disorder | Read |
| TK6.. | Suicide and selfinflicted injury by cutting and stabbing | Mental and behavioural disorder | Read |
| U20y0 | [X]Int self poison/exposure to unspecif chemical at home | Mental and behavioural disorder | Read |
| U26.. | [X]Intentional self harm by explosive material | Mental and behavioural disorder | Read |
| U2010 | [X]Int self poison/exposure to antiepileptic at home | Mental and behavioural disorder | Read |
| U2z.. | [X]Intentional self harm by unspecified means | Mental and behavioural disorder | Read |
| ZX1B1 | Jumping from building | Mental and behavioural disorder | Read |
| U29z. | [X]Intentional self harm by sharp object occ unspecif place | Mental and behavioural disorder | Read |
| TK04. | Suicide + selfinflicted poisoning by other drugs/medicines | Mental and behavioural disorder | Read |
| U2Cy. | [X]Int self harm jump/lying bef mov obje occ oth specif plce | Mental and behavioural disorder | Read |
| TK7z. | Suicide+selfinflicted injury-jump from high place NOS | Mental and behavioural disorder | Read |
| TK52. | Suicide and selfinflicted injury by hunting rifle | Mental and behavioural disorder | Read |
| TK10. | Suicide + selfinflicted poisoning by gas via pipeline | Mental and behavioural disorder | Read |
| U208y | [X]Int self poison oth/unsp drug/medic other spec place | Mental and behavioural disorder | Read |
| TKx00 | Suicide + selfinflicted injury-jumping before moving object | Mental and behavioural disorder | Read |
| 14K1. | Intentional overdose of prescription only medication | Mental and behavioural disorder | Read |
| U30.. | [X]Deliberate drug poisoning | Mental and behavioural disorder | Read |
| TKx4. | Suicide and selfinflicted injury by electrocution | Mental and behavioural disorder | Read |
| U20Cy | [X]Int self poison pesticide other spec place | Mental and behavioural disorder | Read |
| U20C0 |  | Mental and behavioural disorder | Read |
| U207. | [X]Intent self poison/exposure to oth autonomic drug | Mental and behavioural disorder | Read |
| ZX15. | Drowning self | Mental and behavioural disorder | Read |
| TK0z. | Suicide + selfinflicted poisoning by solid/liquid subst NOS | Mental and behavioural disorder | Read |
| U209z | [X]Intent self poison alcohol unspecif place | Mental and behavioural disorder | Read |
| ZX1H2 | Self-suffocation | Mental and behavioural disorder | Read |
| U22z. | [X]Intent self harm by drown/submersn occ unspecified place | Mental and behavioural disorder | Read |
| TK601 | Self inflicted lacerations to wrist | Mental and behavioural disorder | Read |
| U20B. | [X]Intent self poison/exposure to other gas/vapour | Mental and behavioural disorder | Read |
| U45.. | [X]Other+unspecified firearm discharge undetermined intent | Mental and behavioural disorder | Read |
| TK011 | Suicide and self inflicted injury by Barbitone | Mental and behavioural disorder | Read |
| U2D4. | [X]Intent self harm by crash motor vehicl occ street/highway | Mental and behavioural disorder | Read |
| TKx5. | Suicide and selfinflicted injury by crashing motor vehicle | Mental and behavioural disorder | Read |
| U216. | [X]Intent self harm by hang strangl/suffc indust/constr area | Mental and behavioural disorder | Read |
| U20B2 | [X]Int self poison other gas/vapour school/pub admin area | Mental and behavioural disorder | Read |
| TK2.. | Suicide + selfinflicted poisoning by other gases and vapours | Mental and behavioural disorder | Read |
| U2A0. | [X]Intentional self harm by blunt object occurrence at home | Mental and behavioural disorder | Read |
| U2E.. | [X]Self mutilation | Mental and behavioural disorder | Read |
| ZX1B2 | Jumping from bridge | Mental and behavioural disorder | Read |
| ZX1E. | Pinching self | Mental and behavioural disorder | Read |
| U72.. | [X]Sequel intentn self-harm assault+event of undeterm intent | Mental and behavioural disorder | Read |
| TK06. | Suicide + selfinflicted poisoning by agricultural chemical | Mental and behavioural disorder | Read |
| U2y0. | [X]Intentionl self harm by oth specif means occurrn at home | Mental and behavioural disorder | Read |
| U20A0 | [X]Intent self pois organ solvent,halogen hydrocarb, home | Mental and behavioural disorder | Read |
| ZX1J. | Self-electrocution | Mental and behavioural disorder | Read |
| U2004 |  | Mental and behavioural disorder | Read |
| ZX1L1 | Self-mutilation of hands | Mental and behavioural disorder | Read |
| SL... | Overdose of drug | Mental and behavioural disorder | Read |
| SL... | Overdose of biological substance | Mental and behavioural disorder | Read |
| U205. | [X]Intent self poison/exposure to narcotic drug | Mental and behavioural disorder | Read |
| ZRLfC | HoNOS item 2 - non-accidental self injury | Mental and behavioural disorder | Read |
| TK01. | Suicide + selfinflicted poisoning by barbiturates | Mental and behavioural disorder | Read |
| U207z | [X]Intent self poison oth autonomic drug unspecif place | Mental and behavioural disorder | Read |
| U280. | [X]Intent self harm by steam hot vapour/hot obj occ at home | Mental and behavioural disorder | Read |
| U274. | [X]Intent self harm by smoke fire/flame occ street/highway | Mental and behavioural disorder | Read |
| TK3y. | Suicide + selfinflicted inj oth mean hang/strangle/suffocate | Mental and behavioural disorder | Read |
| U211. | [X]Intent self harm by hangng strangult/suffoct resid instit | Mental and behavioural disorder | Read |
| TK7.. | Suicide and selfinflicted injury by jumping from high place | Mental and behavioural disorder | Read |
| U20Bz | [X]Intent self poison other gas/vapour unspecif place | Mental and behavioural disorder | Read |
| U291. | [X]Intent self harm by sharp object occ resident instit'n | Mental and behavioural disorder | Read |
| U2C1. |  | Mental and behavioural disorder | Read |
| ZX1B3 | Jumping from cliff | Mental and behavioural disorder | Read |
| U2z2. | [X]Intent self harm by unspec mean occ sch/ins/pub adm area | Mental and behavioural disorder | Read |
| U20y2 | [X]Int self poison unspecif chemical school/pub admin area | Mental and behavioural disorder | Read |
| U201. | [X]Intent self poison/exposure to antiepileptic | Mental and behavioural disorder | Read |
| U2... | [X]Self inflicted injury | Mental and behavioural disorder | Read |
| U2... | [X]Suicide | Mental and behavioural disorder | Read |
| TK... | Cause of overdose - deliberate | Mental and behavioural disorder | Read |
| U2... | [X]Injury - self-inflicted | Mental and behavioural disorder | Read |
| U2... | [X]Para-suicide | Mental and behavioural disorder | Read |
| U2... | [X]Attempted suicide | Mental and behavioural disorder | Read |
| TK00. | Suicide + selfinflicted poisoning by analgesic/antipyretic | Mental and behavioural disorder | Read |
| U22y. | [X]Intent self harm by drown/submersn occ oth specif place | Mental and behavioural disorder | Read |
| TK... | Para-suicide | Mental and behavioural disorder | Read |
| U202z | [X]Intent self poison sedative hypnotic unspecif place | Mental and behavioural disorder | Read |
| TK... | Injury - self-inflicted | Mental and behavioural disorder | Read |
| TK... | Poisoning - self-inflicted | Mental and behavioural disorder | Read |
| TK... | Suicide and self harm | Mental and behavioural disorder | Read |
| U28z. | [X]Intent self harm by steam hot vapour/obj occ unspec place | Mental and behavioural disorder | Read |
| TK... | Attempted suicide | Mental and behavioural disorder | Read |
| ZX19. | Hitting self | Mental and behavioural disorder | Read |
| U2B6. | [X]Int self harm by jump from high place indust/constr area | Mental and behavioural disorder | Read |
| TKx0. | Suicide + selfinflicted injury-jump/lie before moving object | Mental and behavioural disorder | Read |
| U2005 | [X]Intent self pois nonopioid analgesic trade/service area | Mental and behavioural disorder | Read |
| ZX1L2 | Self-mutilation of genitalia | Mental and behavioural disorder | Read |
| U2020 | [X]Int self poison/exposure to sedative hypnotic at home | Mental and behavioural disorder | Read |
| U2070 | [X]Int self poison/exposure to oth autonomic drug at home | Mental and behavioural disorder | Read |
| U2... | [X]Intentional self-harm | Mental and behavioural disorder | Read |
| SL90. | Antidepressant poisoning | Mental and behavioural disorder | Read |
| TK... | Suicide and selfinflicted injury | Mental and behavioural disorder | Read |
| TK51. | Suicide and selfinflicted injury by shotgun | Mental and behavioural disorder | Read |
| U44.. | [X]Rifle shotgun+larger firearm discharge undetermin intent | Mental and behavioural disorder | Read |
| ZX1.. | Deliberate self-harm | Mental and behavioural disorder | Read |
| ZX1.. | SIB - Self-injurious behaviour | Mental and behavioural disorder | Read |
| U27.. | [X]Intentional self harm by smoke, fire and flames | Mental and behavioural disorder | Read |
| U2y1. | [X]Intent self harm by oth specif means occ resid instit'n | Mental and behavioural disorder | Read |
| U2D6. | [X]Intent self harm crash motor vehic occ indust/constr area | Mental and behavioural disorder | Read |
| TKx1. | Suicide and selfinflicted injury by burns or fire | Mental and behavioural disorder | Read |
| U4B.. | [X]Falling jumping/pushed from high place undeterm intent | Mental and behavioural disorder | Read |
| SLHz. | Drug and medicament poisoning NOS | Mental and behavioural disorder | Read |
| U2z0. | [X]Intentional self harm by unspecif means occurrn at home | Mental and behavioural disorder | Read |
| TK60. | Suicide and selfinflicted injury by cutting | Mental and behavioural disorder | Read |
| ZX13. | Cuts self | Mental and behavioural disorder | Read |
| U4Bz. | [X]Fall jump/push frm high plce undt intnt occ unspecif plce | Mental and behavioural disorder | Read |
| ZX1.. | Self-injurious behaviour | Mental and behavioural disorder | Read |
| TK54. | Suicide and selfinflicted injury by other firearm | Mental and behavioural disorder | Read |
| U250. |  | Mental and behavioural disorder | Read |
| TK1.. | Suicide + selfinflicted poisoning by gases in domestic use | Mental and behavioural disorder | Read |
| TK31. | Suicide + selfinflicted injury by suffocation by plastic bag | Mental and behavioural disorder | Read |
| TK02. | Suicide + selfinflicted poisoning by oth sedatives/hypnotics | Mental and behavioural disorder | Read |
| ZX1I. | Self-scalding | Mental and behavioural disorder | Read |
| U200z | [X]Intent self poison nonopioid analgesic unspecif place | Mental and behavioural disorder | Read |
| U24.. | [X]Intent self harm by rifle shotgun/larger firearm disch | Mental and behavioural disorder | Read |
| TKx6. | Suicide and selfinflicted injury by crashing of aircraft | Mental and behavioural disorder | Read |
| ZX1N. | Stabbing self | Mental and behavioural disorder | Read |
| U290. | [X]Intentional self harm by sharp object occurrence at home | Mental and behavioural disorder | Read |
| U221. | [X]Intent self harm by drowning/submersn occ resid instit'n | Mental and behavioural disorder | Read |
| ZX... | Self-damage | Mental and behavioural disorder | Read |
| U209. | [X]Intent self poison/exposure to alcohol | Mental and behavioural disorder | Read |
| ZX13. | Cutting self | Mental and behavioural disorder | Read |
| ZX1L3 | Self-mutilation of penis | Mental and behavioural disorder | Read |
| U2050 | [X]Int self poison/exposure to narcotic drug at home | Mental and behavioural disorder | Read |
| U2A1. | [X]Intent self harm by blunt object occ resident instit'n | Mental and behavioural disorder | Read |
| U205y | [X]Int self poison narcotic drug other spec place | Mental and behavioural disorder | Read |
| U2yz. | [X]Intent self harm by oth specif means occ unspecif place | Mental and behavioural disorder | Read |
| TK5z. | Suicide and selfinflicted injury by firearms/explosives NOS | Mental and behavioural disorder | Read |
| TK014 | Suicide and self inflicted injury by Phenobarbitone | Mental and behavioural disorder | Read |
| TKx7. | Suicide and selfinflicted injury caustic subst, excl poison | Mental and behavioural disorder | Read |
| U2Bz. | [X]Int self harm by jump from high place occ unspecif place | Mental and behavioural disorder | Read |
| U202. | [X]Overdose - amobarbital | Mental and behavioural disorder | Read |
| U202. | [X]Overdose - barbiturate | Mental and behavioural disorder | Read |
| U202. | [X]Overdose - benzodiazepine | Mental and behavioural disorder | Read |
| U202. | [X]Overdose - nitrazepam | Mental and behavioural disorder | Read |
| U204. | [X]Intent self poison/exposure to psychotropic drug | Mental and behavioural disorder | Read |
| U2084 |  | Mental and behavioural disorder | Read |
| U27z. | [X]Intent self harm by smoke fire/flames occ unspecif place | Mental and behavioural disorder | Read |
| U720. | [X]Sequelae of intentional self-harm | Mental and behavioural disorder | Read |
| U202. | [X]Overdose - temazepam | Mental and behavioural disorder | Read |
| ZX18. | Hanging self | Mental and behavioural disorder | Read |
| U202. | [X]Overdose - diazepam | Mental and behavioural disorder | Read |
| U202. | [X]Overdose - sleeping tabs | Mental and behavioural disorder | Read |
| ZX1C. | Nipping self | Mental and behavioural disorder | Read |
| SL90z | Anti-depressant poisoning NOS | Mental and behavioural disorder | Read |
| ZX1LD | [X]Self mutilation | Mental and behavioural disorder | Read |
| ZX1S. | Throwing self onto floor | Mental and behavioural disorder | Read |
| U200y | [X]Int self poison nonopioid analgesic other spec place | Mental and behavioural disorder | Read |
| TK1y. | Suicide and selfinflicted poisoning by other utility gas | Mental and behavioural disorder | Read |
| U210. | [X]Intent self harm by hanging strangulat/suffocat occ home | Mental and behavioural disorder | Read |
| TK61. | Suicide and selfinflicted injury by stabbing | Mental and behavioural disorder | Read |
| U20Az | [X]Int self pois org solv,halogen hydrocarb, unspec place | Mental and behavioural disorder | Read |
| U204. | [X]Overdose - antidepressant | Mental and behavioural disorder | Read |
| U204. | [X]Overdose - amitriptyline | Mental and behavioural disorder | Read |
| U204. | [X]Overdose - SSRI | Mental and behavioural disorder | Read |
| TK2z. | Suicide + selfinflicted poisoning by gases and vapours NOS | Mental and behavioural disorder | Read |
| TKz.. | Suicide and selfinflicted injury NOS | Mental and behavioural disorder | Read |
| U202. | [X]Intent self poison/exposure to sedative hypnotic | Mental and behavioural disorder | Read |
| TK07. | Suicide + selfinflicted poisoning by corrosive/caustic subst | Mental and behavioural disorder | Read |
| U28.. | [X]Intentional self harm by steam hot vapours / hot objects | Mental and behavioural disorder | Read |
| TK30. | Suicide and selfinflicted injury by hanging | Mental and behavioural disorder | Read |
| ZX1H. | Self-asphyxiation | Mental and behavioural disorder | Read |
| TK72. | Suicide+selfinflicted injury-jump from natural sites | Mental and behavioural disorder | Read |
| U201z | [X]Intent self poison antiepileptic unspecif place | Mental and behavioural disorder | Read |
| ZX1M. | Shooting self | Mental and behavioural disorder | Read |
| ZX12. | Burning self | Mental and behavioural disorder | Read |
| U2041 |  | Mental and behavioural disorder | Read |
| U2064 |  | Mental and behavioural disorder | Read |
| U2A3. | [X]Intent self harm by blunt object occ sports/athlet area | Mental and behavioural disorder | Read |
| TK601 | Slashed wrists self inflicted | Mental and behavioural disorder | Read |
| U204z | [X]Intent self poison psychotropic drug unspecif place | Mental and behavioural disorder | Read |
| U20B. | [X]Self carbon monoxide poisoning | Mental and behavioural disorder | Read |
| U21.. | [X]Intent self harm by hanging strangulation / suffocation | Mental and behavioural disorder | Read |
| TK0.. | Suicide + selfinflicted poisoning by solid/liquid substances | Mental and behavioural disorder | Read |
| U20A4 | [X]Int self poison org solvent,halogen hydrocarb,in highway | Mental and behavioural disorder | Read |
| U2B4. | [X]Intent self harm by jump from high place occ street/h'way | Mental and behavioural disorder | Read |
| U2zz. | [X]Intent self harm by unspecif means occ at unspecif place | Mental and behavioural disorder | Read |
| ZX191 | Punching self | Mental and behavioural disorder | Read |
| TK4.. | Suicide and selfinflicted injury by drowning | Mental and behavioural disorder | Read |
| TKx2. | Suicide and selfinflicted injury by scald | Mental and behavioural disorder | Read |
| TK21. | Suicide and selfinflicted poisoning by other carbon monoxide | Mental and behavioural disorder | Read |
| U41.. | [X]Hanging strangulation + suffocation undetermined intent | Mental and behavioural disorder | Read |
| U294. | [X]Intention self harm by sharp object occ street/highway | Mental and behavioural disorder | Read |
| TK6z. | Suicide and selfinflicted injury by cutting and stabbing NOS | Mental and behavioural disorder | Read |
| U242. |  | Mental and behavioural disorder | Read |
| U2000 | [X]Int self poison/exposure to nonopioid analgesic at home | Mental and behavioural disorder | Read |
| TKxy. | Suicide and selfinflicted injury by other specified means | Mental and behavioural disorder | Read |
| U2C.. | [X]Intent self harm by jumping / lying before moving object | Mental and behavioural disorder | Read |
| ZX1B. | Jumping from height | Mental and behavioural disorder | Read |
| U209y | [X]Int self poison alcohol other spec place | Mental and behavioural disorder | Read |
| TK70. | Suicide+selfinflicted injury-jump from residential premises | Mental and behavioural disorder | Read |
| ZX1R. | Throwing self in front of vehicle | Mental and behavioural disorder | Read |
| TKx3. | Suicide and selfinflicted injury by extremes of cold | Mental and behavioural disorder | Read |
| TK20. | Suicide + selfinflicted poisoning by motor veh exhaust gas | Mental and behavioural disorder | Read |
| U20y. | [X]Intent self poison/exposure to unspecif chemical | Mental and behavioural disorder | Read |
| U220. | [X]Intent self harm by drowning/submersion occurrn at home | Mental and behavioural disorder | Read |
| ZX... | Self-harm | Mental and behavioural disorder | Read |
| U208. | [X]Int self poison/exposure to other/unspec drug/medicament | Mental and behavioural disorder | Read |
| U20.. | [X]Deliberate drug overdose / other poisoning | Mental and behavioural disorder | Read |
| U2040 | [X]Int self poison/exposure to psychotropic drug at home | Mental and behavioural disorder | Read |
| ZX11. | Bites self | Mental and behavioural disorder | Read |
| ZX1G. | Scratches self | Mental and behavioural disorder | Read |
| ZRn3. | Suicide intent score subscale - attempt circumstances | Mental and behavioural disorder | Read |
| TK5.. | Suicide and selfinflicted injury by firearms and explosives | Mental and behavioural disorder | Read |
| TK71. | Suicide+selfinflicted injury-jump from oth manmade structure | Mental and behavioural disorder | Read |
| U204y | [X]Int self poison psychotropic drug other spec place | Mental and behavioural disorder | Read |
| U20C. | [X]Intent self poison/exposure to pesticide | Mental and behavioural disorder | Read |
| U25.. | [X]Intent self harm by other/unspecified firearm discharge | Mental and behavioural disorder | Read |
| U2B.. | [X]Intentional self harm by jumping from a high place | Mental and behavioural disorder | Read |
| U2y.. | [X]Intentional self harm by other specified means | Mental and behavioural disorder | Read |
| U29.. | [X]Intentional self harm by sharp object | Mental and behavioural disorder | Read |
| ZX1Q. | Jumping under train | Mental and behavioural disorder | Read |
| ZX192 | Slapping self | Mental and behavioural disorder | Read |
| ZX1L. | Self-mutilation | Mental and behavioural disorder | Read |
| U21z. | [X]Intent self harm by hangng strangul/suffoct unspecif plce | Mental and behavioural disorder | Read |
| TKy.. | Late effects of selfinflicted injury | Mental and behavioural disorder | Read |
| U2By. | [X]Int self harm by jump from high place occ oth specif plce | Mental and behavioural disorder | Read |
| U2001 |  | Mental and behavioural disorder | Read |
| U2024 |  | Mental and behavioural disorder | Read |
| U2C4. | [X]Int self harm jump/lying befr mov obje occ street/highway | Mental and behavioural disorder | Read |
| ZX1L6 | Self-mutilation of ears | Mental and behavioural disorder | Read |
| U20.. | [X]Intentional self poisoning/exposure to noxious substances | Mental and behavioural disorder | Read |
| U29y. | [X]Intention self harm by sharp object occ oth specif place | Mental and behavioural disorder | Read |
| ZX11. | Biting self | Mental and behavioural disorder | Read |
| ZX131 | Cutting own wrists | Mental and behavioural disorder | Read |
| TK03. | Suicide + selfinflicted poisoning tranquilliser/psychotropic | Mental and behavioural disorder | Read |
| U20C. | [X]Self poisoning with paraquat | Mental and behavioural disorder | Read |
| U20C. | [X]Self poisoning with weedkiller | Mental and behavioural disorder | Read |
| U205z | [X]Intent self poison narcotic drug unspecif place | Mental and behavioural disorder | Read |
| U2D0. | [X]Intent self harm by crash of motor vehicl occurrn at home | Mental and behavioural disorder | Read |
| TK1z. | Suicide + selfinflicted poisoning by domestic gases NOS | Mental and behavioural disorder | Read |
| R0074 | [D]Postviral (asthenic) syndrome | POST VIRAL | Read |
| F286. | Chronic Fatigue Syndrome/Postviral fatigue syndrome | POST VIRAL | Read |
| A37. | Whooping cough | Respiratory | ICD-10 |
| A370 | Whooping cough due to Bordetella pertussis | Respiratory | ICD-10 |
| A371 | Whooping cough due to Bordetella parapertussis | Respiratory | ICD-10 |
| A378 | Whooping cough due to other Bordetella species | Respiratory | ICD-10 |
| A379 | Whooping cough, unspecified | Respiratory | ICD-10 |
| H66. | Suppurative and unspecified otitis media | Respiratory | ICD-10 |
| H660 | Acute suppurative otitis media | Respiratory | ICD-10 |
| H661 | Chronic tubotympanic suppurative otitis media | Respiratory | ICD-10 |
| H662 | Chronic atticoantral suppurative otitis media | Respiratory | ICD-10 |
| H663 | Other chronic suppurative otitis media | Respiratory | ICD-10 |
| H664 | Suppurative otitis media, unspecified | Respiratory | ICD-10 |
| H669 | Otitis media, unspecified | Respiratory | ICD-10 |
| H67. | Otitis media in diseases classified elsewhere | Respiratory | ICD-10 |
| H670 | Otitis media in bacterial diseases classified elsewhere | Respiratory | ICD-10 |
| H671 | Otitis media in viral diseases classified elsewhere | Respiratory | ICD-10 |
| H678 | Otitis media in other diseases classified elsewhere | Respiratory | ICD-10 |
| J00X | Acute nasopharyngitis [common cold] | Respiratory | ICD-10 |
| J01. | Acute sinusitis | Respiratory | ICD-10 |
| J010 | Acute maxillary sinusitis | Respiratory | ICD-10 |
| J011 | Acute frontal sinusitis | Respiratory | ICD-10 |
| J012 | Acute ethmoidal sinusitis | Respiratory | ICD-10 |
| J013 | Acute sphenoidal sinusitis | Respiratory | ICD-10 |
| J014 | Acute pansinusitis | Respiratory | ICD-10 |
| J018 | Other acute sinusitis | Respiratory | ICD-10 |
| J019 | Acute sinusitis, unspecified | Respiratory | ICD-10 |
| J02. | Acute pharyngitis | Respiratory | ICD-10 |
| J020 | Streptococcal pharyngitis | Respiratory | ICD-10 |
| J028 | Acute pharyngitis due to other specified organisms | Respiratory | ICD-10 |
| J029 | Acute pharyngitis, unspecified | Respiratory | ICD-10 |
| J03. | Acute tonsillitis | Respiratory | ICD-10 |
| J030 | Streptococcal tonsillitis | Respiratory | ICD-10 |
| J038 | Acute tonsillitis due to other specified organisms | Respiratory | ICD-10 |
| J039 | Acute tonsillitis, unspecified | Respiratory | ICD-10 |
| J04. | Acute laryngitis and tracheitis | Respiratory | ICD-10 |
| J040 | Acute laryngitis | Respiratory | ICD-10 |
| J041 | Acute tracheitis | Respiratory | ICD-10 |
| J042 | Acute laryngotracheitis | Respiratory | ICD-10 |
| J05. | Acute obstructive laryngitis [croup] and epiglottitis | Respiratory | ICD-10 |
| J050 | Acute obstructive laryngitis [croup] | Respiratory | ICD-10 |
| J051 | Acute epiglottitis | Respiratory | ICD-10 |
| J06. | Acute upper respiratory infections of multiple and unspecified sites | Respiratory | ICD-10 |
| J060 | Acute laryngopharyngitis | Respiratory | ICD-10 |
| J068 | Other acute upper respiratory infections of multiple sites | Respiratory | ICD-10 |
| J069 | Acute upper respiratory infection, unspecified | Respiratory | ICD-10 |
| J09X | Influenza due to identified avian influenza virus | Respiratory | ICD-10 |
| J10. | Influenza due to other identified influenza virus | Respiratory | ICD-10 |
| J100 | Influenza with pneumonia, other influenza virus identified | Respiratory | ICD-10 |
| J101 | Influenza with other respiratory manifestations, other influenza virus identified | Respiratory | ICD-10 |
| J108 | Influenza with other manifestations, other influenza virus identified | Respiratory | ICD-10 |
| J11. | Influenza, virus not identified | Respiratory | ICD-10 |
| J110 | Influenza with pneumonia, virus not identified | Respiratory | ICD-10 |
| J111 | Influenza with other respiratory manifestations, virus not identified | Respiratory | ICD-10 |
| J118 | Influenza with other manifestations, virus not identified | Respiratory | ICD-10 |
| J12. | Viral pneumonia, not elsewhere classified | Respiratory | ICD-10 |
| J120 | Adenoviral pneumonia | Respiratory | ICD-10 |
| J121 | Respiratory syncytial virus pneumonia | Respiratory | ICD-10 |
| J122 | Parainfluenza virus pneumonia | Respiratory | ICD-10 |
| J123 | Human metapneumovirus pneumonia | Respiratory | ICD-10 |
| J128 | Other viral pneumonia | Respiratory | ICD-10 |
| J129 | Viral pneumonia, unspecified | Respiratory | ICD-10 |
| J13X | Pneumonia due to Streptococcus pneumoniae | Respiratory | ICD-10 |
| J14X | Pneumonia due to Haemophilus influenzae | Respiratory | ICD-10 |
| J15. | Bacterial pneumonia, not elsewhere classified | Respiratory | ICD-10 |
| J150 | Pneumonia due to Klebsiella pneumoniae | Respiratory | ICD-10 |
| J151 | Pneumonia due to Pseudomonas | Respiratory | ICD-10 |
| J152 | Pneumonia due to staphylococcus | Respiratory | ICD-10 |
| J153 | Pneumonia due to streptococcus, group B | Respiratory | ICD-10 |
| J154 | Pneumonia due to other streptococci | Respiratory | ICD-10 |
| J155 | Pneumonia due to Escherichia coli | Respiratory | ICD-10 |
| J156 | Pneumonia due to other aerobic Gram-negative bacteria | Respiratory | ICD-10 |
| J157 | Pneumonia due to Mycoplasma pneumoniae | Respiratory | ICD-10 |
| J158 | Other bacterial pneumonia | Respiratory | ICD-10 |
| J159 | Bacterial pneumonia, unspecified | Respiratory | ICD-10 |
| J16. | Pneumonia due to other infectious organisms, not elsewhere classified | Respiratory | ICD-10 |
| J160 | Chlamydial pneumonia | Respiratory | ICD-10 |
| J168 | Pneumonia due to other specified infectious organisms | Respiratory | ICD-10 |
| J17. | Pneumonia in diseases classified elsewhere | Respiratory | ICD-10 |
| J170 | Pneumonia in bacterial diseases classified elsewhere | Respiratory | ICD-10 |
| J171 | Pneumonia in viral diseases classified elsewhere | Respiratory | ICD-10 |
| J172 | Pneumonia in mycoses | Respiratory | ICD-10 |
| J173 | Pneumonia in parasitic diseases | Respiratory | ICD-10 |
| J178 | Pneumonia in other diseases classified elsewhere | Respiratory | ICD-10 |
| J18. | Pneumonia, organism unspecified | Respiratory | ICD-10 |
| J180 | Bronchopneumonia, unspecified | Respiratory | ICD-10 |
| J181 | Lobar pneumonia, unspecified | Respiratory | ICD-10 |
| J182 | Hypostatic pneumonia, unspecified | Respiratory | ICD-10 |
| J188 | Other pneumonia, organism unspecified | Respiratory | ICD-10 |
| J189 | Pneumonia, unspecified | Respiratory | ICD-10 |
| J20. | Acute bronchitis | Respiratory | ICD-10 |
| J200 | Acute bronchitis due to Mycoplasma pneumoniae | Respiratory | ICD-10 |
| J201 | Acute bronchitis due to Haemophilus influenzae | Respiratory | ICD-10 |
| J202 | Acute bronchitis due to streptococcus | Respiratory | ICD-10 |
| J203 | Acute bronchitis due to coxsackievirus | Respiratory | ICD-10 |
| J204 | Acute bronchitis due to parainfluenza virus | Respiratory | ICD-10 |
| J205 | Acute bronchitis due to respiratory syncytial virus | Respiratory | ICD-10 |
| J206 | Acute bronchitis due to rhinovirus | Respiratory | ICD-10 |
| J207 | Acute bronchitis due to echovirus | Respiratory | ICD-10 |
| J208 | Acute bronchitis due to other specified organisms | Respiratory | ICD-10 |
| J209 | Acute bronchitis, unspecified | Respiratory | ICD-10 |
| J21. | Acute bronchiolitis | Respiratory | ICD-10 |
| J210 | Acute bronchiolitis due to respiratory syncytial virus | Respiratory | ICD-10 |
| J211 | Acute bronchiolitis due to human metapneumovirus | Respiratory | ICD-10 |
| J218 | Acute bronchiolitis due to other specified organisms | Respiratory | ICD-10 |
| J219 | Acute bronchiolitis, unspecified | Respiratory | ICD-10 |
| J22X | Unspecified acute lower respiratory infection | Respiratory | ICD-10 |
| J43. | Emphysema | Respiratory | ICD-10 |
| J430 | MacLeod's syndrome | Respiratory | ICD-10 |
| J431 | Panlobular emphysema | Respiratory | ICD-10 |
| J432 | Centrilobular emphysema | Respiratory | ICD-10 |
| J438 | Other emphysema | Respiratory | ICD-10 |
| J439 | Emphysema, unspecified | Respiratory | ICD-10 |
| J44. | Other chronic obstructive pulmonary disease | Respiratory | ICD-10 |
| J440 | Chronic obstructive pulmonary disease with acute lower respiratory infection | Respiratory | ICD-10 |
| J441 | Chronic obstructive pulmonary disease with acute exacerbation, unspecified | Respiratory | ICD-10 |
| J448 | Other specified chronic obstructive pulmonary disease | Respiratory | ICD-10 |
| J449 | Chronic obstructive pulmonary disease, unspecified | Respiratory | ICD-10 |
| J45. | Asthma | Respiratory | ICD-10 |
| J450 | Predominantly allergic asthma | Respiratory | ICD-10 |
| J451 | Nonallergic asthma | Respiratory | ICD-10 |
| J458 | Mixed asthma | Respiratory | ICD-10 |
| J459 | Asthma, unspecified | Respiratory | ICD-10 |
| J46X | Status asthmaticus | Respiratory | ICD-10 |
| P27. | Chronic respiratory disease originating in the perinatal period | Respiratory | ICD-10 |
| P270 | Wilson-Mikity syndrome | Respiratory | ICD-10 |
| P271 | Bronchopulmonary dysplasia originating in the perinatal period | Respiratory | ICD-10 |
| P278 | Other chronic respiratory diseases originating in the perinatal period | Respiratory | ICD-10 |
| P279 | Unspecified chronic respiratory disease originating in the perinatal period | Respiratory | ICD-10 |
| 1780. | Aspirin induced asthma | Respiratory | Read |
| 1781. | Asthma trigger - pollen | Respiratory | Read |
| 1782. | Asthma trigger - tobacco smoke | Respiratory | Read |
| 1783. | Asthma trigger - warm air | Respiratory | Read |
| 1784. | Asthma trigger - emotion | Respiratory | Read |
| 1785. | Asthma trigger - damp | Respiratory | Read |
| 1786. | Asthma trigger - animals | Respiratory | Read |
| 1787. | Asthma trigger - seasonal | Respiratory | Read |
| 1788. | Asthma trigger - cold air | Respiratory | Read |
| 1789. | Asthma trigger - respiratory infection | Respiratory | Read |
| 663e1 | Asthma severely restricts exercise | Respiratory | Read |
| 6635. | Increasing exercise wheeze | Respiratory | Read |
| 8793. | Asthma control step 0 | Respiratory | Read |
| 8794. | Asthma control step 1 | Respiratory | Read |
| 8795. | Asthma control step 2 | Respiratory | Read |
| 8796. | Asthma control step 3 | Respiratory | Read |
| 8797. | Asthma control step 4 | Respiratory | Read |
| 8798. | Asthma control step 5 | Respiratory | Read |
| 173A. | Exercise induced asthma | Respiratory | Read |
| 178.. | Asthma trigger | Respiratory | Read |
| 178A. | Asthma trigger - airborne dust | Respiratory | Read |
| 178B. | Asthma trigger - exercise | Respiratory | Read |
| 1J70. | Suspected asthma | Respiratory | Read |
| 1O2.. | Asthma confirmed | Respiratory | Read |
| 663.. | Respiratory disease monitoring | Respiratory | Read |
| 663d. | Emergency asthma admission since last appointment | Respiratory | Read |
| 663e. | Asthma restricts exercise | Respiratory | Read |
| 663f. | Asthma never restricts exercise | Respiratory | Read |
| 663j. | Asthma - currently active | Respiratory | Read |
| 663J. | Airways obstruction reversible | Respiratory | Read |
| 663m. | Asthma accident and emergency attendance since last visit | Respiratory | Read |
| 663n. | Asthma treatment compliance satisfactory | Respiratory | Read |
| 663N. | Asthma disturbing sleep | Respiratory | Read |
| 663N0 | Asthma causing night waking | Respiratory | Read |
| 663N1 | Asthma disturbs sleep weekly | Respiratory | Read |
| 663N2 | Asthma disturbs sleep frequently | Respiratory | Read |
| 663O. | Asthma not disturbing sleep | Respiratory | Read |
| 663O0 | Asthma never disturbs sleep | Respiratory | Read |
| 663p. | Asthma treatment compliance unsatisfactory | Respiratory | Read |
| 663P. | Asthma limiting activities | Respiratory | Read |
| 663P0 | Asthma limits activities 1 to 2 times per month | Respiratory | Read |
| 663P1 | Asthma limits activities 1 to 2 times per week | Respiratory | Read |
| 663P2 | Asthma limits activities most days | Respiratory | Read |
| 663q. | Asthma daytime symptoms | Respiratory | Read |
| 663Q. | Asthma not limiting activities | Respiratory | Read |
| 663r. | Asthma causes night symptoms 1 to 2 times per month | Respiratory | Read |
| 663s. | Asthma never causes daytime symptoms | Respiratory | Read |
| 663t. | Asthma causes daytime symptoms 1 to 2 times per month | Respiratory | Read |
| 663u. | Asthma causes daytime symptoms 1 to 2 times per week | Respiratory | Read |
| 663U. | Asthma management plan given | Respiratory | Read |
| 663v. | Asthma causes daytime symptoms most days | Respiratory | Read |
| 663V. | Asthma severity | Respiratory | Read |
| 663V0 | Occasional asthma | Respiratory | Read |
| 663V1 | Mild asthma | Respiratory | Read |
| 663V2 | Moderate asthma | Respiratory | Read |
| 663V3 | Severe asthma | Respiratory | Read |
| 663w. | Asthma limits walking up hills or stairs | Respiratory | Read |
| 663W. | Asthma prophylactic medication used | Respiratory | Read |
| 663x. | Asthma limits walking on the flat | Respiratory | Read |
| 663y. | Number of asthma exacerbations in past year | Respiratory | Read |
| 66Y9. | Step up change in asthma management plan | Respiratory | Read |
| 66YC. | Absent from work or school due to asthma | Respiratory | Read |
| 66YK. | Asthma follow-up | Respiratory | Read |
| 66YP. | Asthma night-time symptoms | Respiratory | Read |
| 66Yq. | Asthma causes night time symptoms 1 to 2 times per week | Respiratory | Read |
| 66Yr. | Asthma causes symptoms most nights | Respiratory | Read |
| 8H2P. | Emergency admission, asthma | Respiratory | Read |
| H3120 | Chronic asthmatic bronchitis | Respiratory | Read |
| H33.. | Asthma | Respiratory | Read |
| H330. | Extrinsic (atopic) asthma | Respiratory | Read |
| H3300 | Extrinsic asthma without status asthmaticus | Respiratory | Read |
| H3301 | Extrinsic asthma with status asthmaticus | Respiratory | Read |
| H330z | Extrinsic asthma NOS | Respiratory | Read |
| H331. | Intrinsic asthma | Respiratory | Read |
| H3310 | Intrinsic asthma without status asthmaticus | Respiratory | Read |
| H3311 | Intrinsic asthma with status asthmaticus | Respiratory | Read |
| H331z | Intrinsic asthma NOS | Respiratory | Read |
| H332. | Mixed asthma | Respiratory | Read |
| H333. | Acute exacerbation of asthma | Respiratory | Read |
| H334. | Brittle asthma | Respiratory | Read |
| H335. | Chronic asthma with fixed airflow obstruction | Respiratory | Read |
| H33z. | Asthma unspecified | Respiratory | Read |
| H33z0 | Status asthmaticus NOS | Respiratory | Read |
| H33z1 | Asthma attack | Respiratory | Read |
| H33z2 | Late-onset asthma | Respiratory | Read |
| H33zz | Asthma NOS | Respiratory | Read |
| H35y7 | Wood asthma | Respiratory | Read |
| H47y0 | Detergent asthma | Respiratory | Read |
| 1737. | Wheezing | Respiratory | Read |
| 2326. | O/E - expiratory wheeze | Respiratory | Read |
| 173B. | Nocturnal cough / wheeze | Respiratory | Read |
| 173e. | Viral wheeze | Respiratory | Read |
| 23D2. | O/E - rhonchi present | Respiratory | Read |
| 663a. | Oral steroids used since last appointment | Respiratory | Read |
| 663b. | Home nebuliser used since last appointment | Respiratory | Read |
| 663c. | Nebulisation since last appointment | Respiratory | Read |
| 663F. | Oral steroids started | Respiratory | Read |
| 663G. | Oral steroids stopped | Respiratory | Read |
| 663g1 | Using inhaled steroids - normal dose | Respiratory | Read |
| 663g2 | Using inhaled steroids - high dose | Respiratory | Read |
| 663g3 | Increases inhaled steroids appropriately | Respiratory | Read |
| 663g4 | Using inhaled steroids - low dose | Respiratory | Read |
| 663l. | Spacer device in use | Respiratory | Read |
| 663L. | Bronchodilators used more than once daily | Respiratory | Read |
| 663M. | Bronchodilators used a maximum of once daily | Respiratory | Read |
| 663S. | Peak flow meter at home | Respiratory | Read |
| 663T. | No peak flow meter at home | Respiratory | Read |
| 663Y. | Steroid dose inhaled daily | Respiratory | Read |
| 663z. | Number of times bronchodilator used in one week | Respiratory | Read |
| 663Z0 | Bronchodilator used infrequently | Respiratory | Read |
| 663Z1 | Bronchodilator not used in last month | Respiratory | Read |
| H3121 | Emphysematous bronchitis | Respiratory | Read |
| H32.. | Emphysema | Respiratory | Read |
| H4640 | Chronic emphysema due to chemical fumes | Respiratory | Read |
| H581. | Interstitial emphysema | Respiratory | Read |
| H5y16 | Bronchospasm | Respiratory | Read |
| R0609 | [D]Wheezing | Respiratory | Read |
| R060E | [D]Mild wheeze | Respiratory | Read |
| R060F | [D]Moderate wheeze | Respiratory | Read |
| R060G | [D]Severe wheeze | Respiratory | Read |
| R060H | [D]Very severe wheeze | Respiratory | Read |
| 14B3. | H/O: chr.obstr. airway disease | Respiratory | Read |
| 663K. | Airways obstructn irreversible | Respiratory | Read |
| 66YL. | Chronic obstructive pulmonary disease follow-up | Respiratory | Read |
| H31.. | Chronic bronchitis | Respiratory | Read |
| H36.. | Mild chronic obstructive pulmonary disease | Respiratory | Read |
| H37.. | Moderate chronic obstructive pulmonary disease | Respiratory | Read |
| H38.. | Severe chronic obstructive pulmonary disease | Respiratory | Read |
| H39.. | Very severe chronic obstructive pulmonary disease | Respiratory | Read |
| H3A.. | End stage chronic obstructive airways disease | Respiratory | Read |
| H3y.. | Other specified chronic obstructive airways disease | Respiratory | Read |
| H3z.. | Chronic obstructive airways disease NOS | Respiratory | Read |
| 1825. | Pleuritic pain | Respiratory | Read |
| 1827. | Painful breathing -pleurodynia | Respiratory | Read |
| 23D5. | O/E - pleural friction rub | Respiratory | Read |
| 4JU9. | Respiratory syncytial virus A detected | Respiratory | Read |
| 4JUA. | Respiratory syncytial virus B detected | Respiratory | Read |
| 4JUB. | Respiratory syncytial virus untyped strain detected | Respiratory | Read |
| 4JUK. | Mycoplasma pneumoniae detected | Respiratory | Read |
| A0222 | Salmonella pneumonia | Respiratory | Read |
| A521. | Varicella pneumonitis | Respiratory | Read |
| A54x4 | Herpes simplex pneumonia | Respiratory | Read |
| A551. | Postmeasles pneumonia | Respiratory | Read |
| A730. | Ornithosis with pneumonia | Respiratory | Read |
| A741. | Epidemic pleurodynia | Respiratory | Read |
| A7850 | Cytomegaloviral pneumonitis | Respiratory | Read |
| A79A. | Respiratory syncytial virus infection | Respiratory | Read |
| A7y02 | Resp syncytial virus as cause of dis class to other chapters | Respiratory | Read |
| AyuKN | [X]Resp syncytial virus/cause/diseases classfd/oth chapters | Respiratory | Read |
| H06.. | Acute bronchitis and bronchiolitis | Respiratory | Read |
| H060. | Acute bronchitis | Respiratory | Read |
| H0600 | Acute fibrinous bronchitis | Respiratory | Read |
| H0601 | Acute membranous bronchitis | Respiratory | Read |
| H0602 | Acute pseudomembranous bronchitis | Respiratory | Read |
| H0603 | Acute purulent bronchitis | Respiratory | Read |
| H0604 | Acute croupous bronchitis | Respiratory | Read |
| H0605 | Acute tracheobronchitis | Respiratory | Read |
| H0606 | Acute pneumococcal bronchitis | Respiratory | Read |
| H0607 | Acute streptococcal bronchitis | Respiratory | Read |
| H0608 | Acute haemophilus influenzae bronchitis | Respiratory | Read |
| H0609 | Acute neisseria catarrhalis bronchitis | Respiratory | Read |
| H060A | Acute bronchitis due to mycoplasma pneumoniae | Respiratory | Read |
| H060B | Acute bronchitis due to coxsackievirus | Respiratory | Read |
| H060C | Acute bronchitis due to parainfluenza virus | Respiratory | Read |
| H060D | Acute bronchitis due to respiratory syncytial virus | Respiratory | Read |
| H060E | Acute bronchitis due to rhinovirus | Respiratory | Read |
| H060F | Acute bronchitis due to echovirus | Respiratory | Read |
| H060v | Subacute bronchitis unspecified | Respiratory | Read |
| H060w | Acute viral bronchitis unspecified | Respiratory | Read |
| H060x | Acute bacterial bronchitis unspecified | Respiratory | Read |
| H060z | Acute bronchitis NOS | Respiratory | Read |
| H061. | Acute bronchiolitis | Respiratory | Read |
| H0610 | Acute capillary bronchiolitis | Respiratory | Read |
| H0611 | Acute obliterating bronchiolitis | Respiratory | Read |
| H0612 | Acute bronchiolitis with bronchospasm | Respiratory | Read |
| H0613 | Acute exudative bronchiolitis | Respiratory | Read |
| H0614 | Obliterating fibrous bronchiolitis | Respiratory | Read |
| H0615 | Acute bronchiolitis due to respiratory syncytial virus | Respiratory | Read |
| H0616 | Acute bronchiolitis due to other specified organisms | Respiratory | Read |
| H0617 | Acute bronchiolitis due to human metapneumovirus | Respiratory | Read |
| H061z | Acute bronchiolitis NOS | Respiratory | Read |
| H062. | Acute lower respiratory tract infection | Respiratory | Read |
| H06z. | Acute bronchitis or bronchiolitis NOS | Respiratory | Read |
| H06z1 | Lower resp tract infection | Respiratory | Read |
| H07.. | Chest cold | Respiratory | Read |
| H2... | Pneumonia and influenza | Respiratory | Read |
| H20.. | Viral pneumonia | Respiratory | Read |
| H200. | Pneumonia due to adenovirus | Respiratory | Read |
| H201. | Pneumonia due to respiratory syncytial virus | Respiratory | Read |
| H202. | Pneumonia due to parainfluenza virus | Respiratory | Read |
| H203. | Pneumonia due to human metapneumovirus | Respiratory | Read |
| H20y. | Viral pneumonia NEC | Respiratory | Read |
| H20y0 | Severe acute respiratory syndrome | Respiratory | Read |
| H20z. | Viral pneumonia NOS | Respiratory | Read |
| H21.. | Lobar (pneumococcal) pneumonia | Respiratory | Read |
| H22.. | Other bacterial pneumonia | Respiratory | Read |
| H23.. | Pneumonia due to other specified organisms | Respiratory | Read |
| H231. | Pneumonia due to mycoplasma pneumoniae | Respiratory | Read |
| H232. | Pneumonia due to pleuropneumonia like organisms | Respiratory | Read |
| H233. | Chlamydial pneumonia | Respiratory | Read |
| H23z. | Pneumonia due to specified organism NOS | Respiratory | Read |
| H24.. | Pneumonia with infectious diseases EC | Respiratory | Read |
| H240. | Pneumonia with measles | Respiratory | Read |
| H241. | Pneumonia with cytomegalic inclusion disease | Respiratory | Read |
| H243. | Pneumonia with whooping cough | Respiratory | Read |
| H24y. | Pneumonia with other infectious diseases EC | Respiratory | Read |
| H24y7 | Pneumonia with varicella | Respiratory | Read |
| H24yz | Pneumonia with other infectious diseases EC NOS | Respiratory | Read |
| H24z. | Pneumonia with infectious diseases EC NOS | Respiratory | Read |
| H25.. | Bronchopneumonia due to unspecified organism | Respiratory | Read |
| H26.. | Pneumonia due to unspecified organism | Respiratory | Read |
| H260. | Lobar pneumonia due to unspecified organism | Respiratory | Read |
| H261. | Basal pneumonia due to unspecified organism | Respiratory | Read |
| H263. | Pneumonitis, unspecified | Respiratory | Read |
| H270. | Influenza with pneumonia | Respiratory | Read |
| H2700 | Influenza with bronchopneumonia | Respiratory | Read |
| H2701 | Influenza with pneumonia, influenza virus identified | Respiratory | Read |
| H270z | Influenza with pneumonia NOS | Respiratory | Read |
| H28.. | Atypical pneumonia | Respiratory | Read |
| H2y.. | Other specified pneumonia or influenza | Respiratory | Read |
| H2z.. | Pneumonia or influenza NOS | Respiratory | Read |
| H30.. | Bronchitis unspecified | Respiratory | Read |
| H300. | Tracheobronchitis NOS | Respiratory | Read |
| H301. | Laryngotracheobronchitis | Respiratory | Read |
| H302. | Wheezy bronchitis | Respiratory | Read |
| H30z. | Bronchitis NOS | Respiratory | Read |
| H357. | Ventilation pneumonitis | Respiratory | Read |
| H5010 | Pleural abscess | Respiratory | Read |
| H5012 | Pleural empyema | Respiratory | Read |
| H5014 | Purulent pleurisy | Respiratory | Read |
| H51.. | Pleurisy | Respiratory | Read |
| H510. | Pleurisy without effusion or active tuberculosis | Respiratory | Read |
| H5103 | Acute dry pleurisy | Respiratory | Read |
| H5104 | Diaphragmatic pleurisy | Respiratory | Read |
| H5105 | Basal pleurisy | Respiratory | Read |
| H5109 | Pneumococcal pleurisy | Respiratory | Read |
| H510A | Staphylococcal pleurisy | Respiratory | Read |
| H510B | Streptococcal pleurisy | Respiratory | Read |
| H510z | Pleurisy without effusion or active tuberculosis NOS | Respiratory | Read |
| H511. | Bacterial pleurisy with effusion | Respiratory | Read |
| H5110 | Pneumococcal pleurisy with effusion | Respiratory | Read |
| H5111 | Staphylococcal pleurisy with effusion | Respiratory | Read |
| H511z | Bacterial pleurisy with effusion NOS | Respiratory | Read |
| H51y. | Other pleural effusion excluding mention of tuberculosis | Respiratory | Read |
| H5303 | Abscess of lung with pneumonia | Respiratory | Read |
| H564. | Bronchiolitis obliterans organising pneumonia | Respiratory | Read |
| H56y1 | Interstitial pneumonia | Respiratory | Read |
| Hyu08 | [X]Other viral pneumonia | Respiratory | Read |
| Hyu0A | [X]Other bacterial pneumonia | Respiratory | Read |
| Hyu0B | [X]Pneumonia due to other specified infectious organisms | Respiratory | Read |
| Hyu0D | [X]Pneumonia in viral diseases classified elsewhere | Respiratory | Read |
| Hyu0H | [X]Other pneumonia, organism unspecified | Respiratory | Read |
| Hyu1. | [X]Other acute lower respiratory infections | Respiratory | Read |
| Hyu10 | [X]Acute bronchitis due to other specified organisms | Respiratory | Read |
| Hyu11 | [X]Acute bronchiolitis due to other specified organisms | Respiratory | Read |
| R0654 | [D]Pleuritic pain | Respiratory | Read |
| R0655 | [D]Pleurodynia | Respiratory | Read |
| 1656. | Feverish cold | Respiratory | Read |
| 75311 | Drainage of peritonsillar abscess | Respiratory | Read |
| 16L.. | Influenza-like symptoms | Respiratory | Read |
| 1C3.. | Earache symptoms | Respiratory | Read |
| 1C32. | Unilateral earache | Respiratory | Read |
| 1C33. | Bilateral earache | Respiratory | Read |
| 1C3Z. | Earache symptom NOS | Respiratory | Read |
| 1C9.. | Sore throat symptom | Respiratory | Read |
| 1C92. | Has a sore throat | Respiratory | Read |
| 1C93. | Persistent sore throat | Respiratory | Read |
| 1C9Z. | Sore throat symptom NOS | Respiratory | Read |
| 1CB3. | Throat pain | Respiratory | Read |
| 2D95. | O/E - tympanic membrane red | Respiratory | Read |
| 2DB6. | O/E - follicular tonsillitis | Respiratory | Read |
| 2DB7. | O/E - exudate on tonsils | Respiratory | Read |
| 2DC1. | O/E - pharynx hyperaemic | Respiratory | Read |
| 2DC2. | O/E - granular pharyngitis | Respiratory | Read |
| 2DC3. | Inflamed throat | Respiratory | Read |
| 4J3L. | Influenza A virus H1N1 subtype detected | Respiratory | Read |
| 4JF40 | Throat swab culture positive | Respiratory | Read |
| 4JU0. | Influenza H1 virus detected | Respiratory | Read |
| 4JU1. | Influenza H2 virus detected | Respiratory | Read |
| 4JU2. | Influenza H3 virus detected | Respiratory | Read |
| 4JU3. | Influenza H5 virus detected | Respiratory | Read |
| 4JU4. | Influenza A virus, other or untyped strain detected | Respiratory | Read |
| 4JU5. | Influenza B virus detected | Respiratory | Read |
| 4JUE. | Human rhinovirus detected | Respiratory | Read |
| 4JUF. | Human parainfluenza virus detected | Respiratory | Read |
| 65VA. | Notification of whooping cough | Respiratory | Read |
| A32.. | Diphtheria | Respiratory | Read |
| A320. | Faucial diphtheria | Respiratory | Read |
| A321. | Nasopharyngeal diphtheria | Respiratory | Read |
| A322. | Anterior nasal diphtheria | Respiratory | Read |
| A323. | Laryngeal diphtheria | Respiratory | Read |
| A32yz | Other specified diphtheria NOS | Respiratory | Read |
| A32z. | Diphtheria NOS | Respiratory | Read |
| A33.. | Whooping cough | Respiratory | Read |
| A330. | Bordetella pertussis | Respiratory | Read |
| A331. | Bordetella parapertussis | Respiratory | Read |
| A33y. | Whooping cough - other specified organism | Respiratory | Read |
| A33y0 | Bordetella bronchiseptica | Respiratory | Read |
| A33yz | Other whooping cough NOS | Respiratory | Read |
| A33z. | Whooping cough NOS | Respiratory | Read |
| A34.. | Streptococcal sore throat and scarlatina | Respiratory | Read |
| A340. | Streptococcal sore throat | Respiratory | Read |
| A3400 | Streptococcal angina | Respiratory | Read |
| A3401 | Streptococcal laryngitis | Respiratory | Read |
| A3402 | Streptococcal pharyngitis | Respiratory | Read |
| A3403 | Streptococcal tonsillitis | Respiratory | Read |
| A340z | Streptococcal sore throat NOS | Respiratory | Read |
| A341. | Scarlet fever - scarlatina | Respiratory | Read |
| A34z. | Streptococcal sore throat with scarlatina NOS | Respiratory | Read |
| A552. | Postmeasles otitis media | Respiratory | Read |
| A793. | Rhinovirus | Respiratory | Read |
| Ayu38 | [X]Diphtheria, unspecified | Respiratory | Read |
| Ayu3A | [X]Whooping cough, unspecified | Respiratory | Read |
| F51.. | Nonsuppurative otitis media + eustachian tube disorders | Respiratory | Read |
| F510. | Acute non suppurative otitis media | Respiratory | Read |
| F5100 | Acute otitis media with effusion | Respiratory | Read |
| F5101 | Acute serous otitis media | Respiratory | Read |
| F5102 | Acute mucoid otitis media | Respiratory | Read |
| F5103 | Acute sanguinous otitis media | Respiratory | Read |
| F5104 | Acute allergic serous otitis media | Respiratory | Read |
| F5106 | Acute allergic sanguinous otitis media | Respiratory | Read |
| F510z | Acute nonsuppurative otitis media NOS | Respiratory | Read |
| F514. | Unspecified nonsuppurative otitis media | Respiratory | Read |
| F5141 | Serous otitis media NOS | Respiratory | Read |
| F5142 | Catarrhal otitis media NOS | Respiratory | Read |
| F5143 | Mucoid otitis media NOS | Respiratory | Read |
| F514z | Nonsuppurative otitis media NOS | Respiratory | Read |
| F515. | Eustachian tube salpingitis | Respiratory | Read |
| F5150 | Unspecified eustachian tube salpingitis | Respiratory | Read |
| F5151 | Acute eustachian tube salpingitis | Respiratory | Read |
| F515z | Eustachian tube salpingitis NOS | Respiratory | Read |
| F52.. | Suppurative and unspecified otitis media | Respiratory | Read |
| F520. | Acute suppurative otitis media | Respiratory | Read |
| F5200 | Acute suppurative otitis media tympanic membrane intact | Respiratory | Read |
| F5201 | Acute suppurative otitis media tympanic membrane ruptured | Respiratory | Read |
| F5203 | Acute suppurative otitis media due to disease EC | Respiratory | Read |
| F520z | Acute suppurative otitis media NOS | Respiratory | Read |
| F524. | Purulent otitis media NOS | Respiratory | Read |
| F5240 | Bilateral suppurative otitis media | Respiratory | Read |
| F525. | Recurrent acute otitis media | Respiratory | Read |
| F526. | Acute left otitis media | Respiratory | Read |
| F527. | Acute right otitis media | Respiratory | Read |
| F528. | Acute bilateral otitis media | Respiratory | Read |
| F52z. | Otitis media NOS | Respiratory | Read |
| F53.. | Mastoiditis and related conditions | Respiratory | Read |
| F530. | Acute mastoiditis | Respiratory | Read |
| F5300 | Acute mastoiditis without complications | Respiratory | Read |
| F5301 | Subperiosteal mastoid abscess | Respiratory | Read |
| F5302 | Gradenigo's syndrome | Respiratory | Read |
| F5303 | Acute mastoiditis with other complication | Respiratory | Read |
| F530z | Acute mastoiditis NOS | Respiratory | Read |
| F540. | Acute myringitis without otitis media | Respiratory | Read |
| F5401 | Unspecified acute tympanitis | Respiratory | Read |
| F5402 | Bullous myringitis | Respiratory | Read |
| F540z | Acute myringitis NOS | Respiratory | Read |
| F587. | Otalgia | Respiratory | Read |
| F5870 | Unspecified otalgia | Respiratory | Read |
| F5871 | Otogenic pain | Respiratory | Read |
| F587z | Otalgia NOS | Respiratory | Read |
| FyuP0 | [X]Other acute nonsuppurative otitis media | Respiratory | Read |
| FyuP3 | [X]Otitis media in bacterial diseases classified elsewhere | Respiratory | Read |
| FyuP4 | [X]Otitis media in viral diseases classified elsewhere | Respiratory | Read |
| FyuP5 | [X]Otitis media in other diseases classified elsewhere | Respiratory | Read |
| FyuP7 | [X]Other mastoiditis and related conditions | Respiratory | Read |
| H00.. | Acute nasopharyngitis | Respiratory | Read |
| H01.. | Acute sinusitis | Respiratory | Read |
| H010. | Acute maxillary sinusitis | Respiratory | Read |
| H011. | Acute frontal sinusitis | Respiratory | Read |
| H012. | Acute ethmoidal sinusitis | Respiratory | Read |
| H013. | Acute sphenoidal sinusitis | Respiratory | Read |
| H014. | Acute rhinosinusitis | Respiratory | Read |
| H01y. | Other acute sinusitis | Respiratory | Read |
| H01y0 | Acute pansinusitis | Respiratory | Read |
| H01yz | Other acute sinusitis NOS | Respiratory | Read |
| H01z. | Acute sinusitis NOS | Respiratory | Read |
| H02.. | Acute pharyngitis | Respiratory | Read |
| H020. | Acute gangrenous pharyngitis | Respiratory | Read |
| H021. | Acute phlegmonous pharyngitis | Respiratory | Read |
| H022. | Acute ulcerative pharyngitis | Respiratory | Read |
| H023. | Acute bacterial pharyngitis | Respiratory | Read |
| H0230 | Acute pneumococcal pharyngitis | Respiratory | Read |
| H0231 | Acute staphylococcal pharyngitis | Respiratory | Read |
| H023z | Acute bacterial pharyngitis NOS | Respiratory | Read |
| H024. | Acute viral pharyngitis | Respiratory | Read |
| H02z. | Acute pharyngitis NOS | Respiratory | Read |
| H03.. | Acute tonsillitis | Respiratory | Read |
| H030. | Acute erythematous tonsillitis | Respiratory | Read |
| H031. | Acute follicular tonsillitis | Respiratory | Read |
| H032. | Acute ulcerative tonsillitis | Respiratory | Read |
| H033. | Acute catarrhal tonsillitis | Respiratory | Read |
| H034. | Acute gangrenous tonsillitis | Respiratory | Read |
| H035. | Acute bacterial tonsillitis | Respiratory | Read |
| H0350 | Acute pneumococcal tonsillitis | Respiratory | Read |
| H0351 | Acute staphylococcal tonsillitis | Respiratory | Read |
| H035z | Acute bacterial tonsillitis NOS | Respiratory | Read |
| H036. | Acute viral tonsillitis | Respiratory | Read |
| H037. | Recurrent acute tonsillitis | Respiratory | Read |
| H03z. | Acute tonsillitis NOS | Respiratory | Read |
| H04.. | Acute laryngitis and tracheitis | Respiratory | Read |
| H040. | Acute laryngitis | Respiratory | Read |
| H0400 | Acute oedematous laryngitis | Respiratory | Read |
| H0401 | Acute ulcerative laryngitis | Respiratory | Read |
| H0402 | Acute catarrhal laryngitis | Respiratory | Read |
| H0403 | Acute phlegmonous laryngitis | Respiratory | Read |
| H0404 | Acute haemophilus influenzae laryngitis | Respiratory | Read |
| H0405 | Acute pneumococcal laryngitis | Respiratory | Read |
| H0406 | Acute suppurative laryngitis | Respiratory | Read |
| H040w | Acute viral laryngitis unspecified | Respiratory | Read |
| H040x | Acute bacterial laryngitis unspecified | Respiratory | Read |
| H040z | Acute laryngitis NOS | Respiratory | Read |
| H042. | Acute laryngotracheitis | Respiratory | Read |
| H0420 | Acute laryngotracheitis without obstruction | Respiratory | Read |
| H0421 | Acute laryngotracheitis with obstruction | Respiratory | Read |
| H042z | Acute laryngotracheitis NOS | Respiratory | Read |
| H043. | Acute epiglottitis (non strep) | Respiratory | Read |
| H0430 | Acute epiglottitis without obstruction | Respiratory | Read |
| H0431 | Acute epiglottitis with obstruction | Respiratory | Read |
| H0432 | Acute obstructive laryngitis | Respiratory | Read |
| H043z | Acute epiglottitis NOS | Respiratory | Read |
| H044. | Croup | Respiratory | Read |
| H04z. | Acute laryngitis and tracheitis NOS | Respiratory | Read |
| H05.. | Other acute upper respiratory infections | Respiratory | Read |
| H050. | Acute laryngopharyngitis | Respiratory | Read |
| H051. | Acute upper respiratory tract infection | Respiratory | Read |
| H052. | Pharyngotracheitis | Respiratory | Read |
| H053. | Tracheopharyngitis | Respiratory | Read |
| H054. | Recurrent upper respiratory tract infection | Respiratory | Read |
| H055. | Pharyngolaryngitis | Respiratory | Read |
| H05y. | Other upper respiratory infections of multiple sites | Respiratory | Read |
| H05z. | Upper respiratory infection NOS | Respiratory | Read |
| H1201 | Chronic catarrhal rhinitis | Respiratory | Read |
| H1211 | Atrophic pharyngitis | Respiratory | Read |
| H1212 | Granular pharyngitis | Respiratory | Read |
| H1213 | Hypertrophic pharyngitis | Respiratory | Read |
| H130. | Chronic maxillary sinusitis | Respiratory | Read |
| H131. | Chronic frontal sinusitis | Respiratory | Read |
| H135. | Recurrent sinusitis | Respiratory | Read |
| H13y1 | Pansinusitis | Respiratory | Read |
| H141. | Tonsil and/or adenoid hypertrophy | Respiratory | Read |
| H15.. | Peritonsillar abscess - quinsy | Respiratory | Read |
| H1y1z | Nasal cavity and sinus disease NOS | Respiratory | Read |
| H1y22 | Parapharyngeal abscess | Respiratory | Read |
| H1y23 | Retropharyngeal abscess | Respiratory | Read |
| H1y26 | Pharynx or nasopharynx abscess | Respiratory | Read |
| H27.. | Influenza | Respiratory | Read |
| H2710 | Influenza with laryngitis | Respiratory | Read |
| H2711 | Influenza with pharyngitis | Respiratory | Read |
| H27z. | Influenza NOS | Respiratory | Read |
| H29.. | Avian influenza | Respiratory | Read |
| H2A.. | Influenza due to Influenza A virus subtype H1N1 | Respiratory | Read |
| Hyu0. | [X]Acute upper respiratory infections | Respiratory | Read |
| Hyu00 | [X]Other acute sinusitis | Respiratory | Read |
| Hyu01 | [X]Acute pharyngitis due to other specified organisms | Respiratory | Read |
| Hyu02 | [X]Acute tonsillitis due to other specified organisms | Respiratory | Read |
| Hyu04 | [X]Flu+oth respiratory manifestations,'flu virus identified | Respiratory | Read |
| Hyu05 | [X]Influenza+other manifestations,influenza virus identified | Respiratory | Read |
| Hyu06 | [X]Influenza+oth respiratory manifestatns,virus not identifd | Respiratory | Read |
| Hyu07 | [X]Influenza+other manifestations, virus not identified | Respiratory | Read |
| R041. | [D]Throat pain | Respiratory | Read |
| R1532 | [D]Positive culture findings in throat | Respiratory | Read |
| 1780. | Aspirin induced asthma | Respiratory | Read |
| 1781. | Asthma trigger - pollen | Respiratory | Read |
| 1782. | Asthma trigger - tobacco smoke | Respiratory | Read |
| 1783. | Asthma trigger - warm air | Respiratory | Read |
| 1784. | Asthma trigger - emotion | Respiratory | Read |
| 1785. | Asthma trigger - damp | Respiratory | Read |
| 1786. | Asthma trigger - animals | Respiratory | Read |
| 1787. | Asthma trigger - seasonal | Respiratory | Read |
| 1788. | Asthma trigger - cold air | Respiratory | Read |
| 1789. | Asthma trigger - respiratory infection | Respiratory | Read |
| 663e1 | Asthma severely restricts exercise | Respiratory | Read |
| 6635. | Increasing exercise wheeze | Respiratory | Read |
| 8793. | Asthma control step 0 | Respiratory | Read |
| 8794. | Asthma control step 1 | Respiratory | Read |
| 8795. | Asthma control step 2 | Respiratory | Read |
| 8796. | Asthma control step 3 | Respiratory | Read |
| 8797. | Asthma control step 4 | Respiratory | Read |
| 8798. | Asthma control step 5 | Respiratory | Read |
| 173A. | Exercise induced asthma | Respiratory | Read |
| 178.. | Asthma trigger | Respiratory | Read |
| 178A. | Asthma trigger - airborne dust | Respiratory | Read |
| 178B. | Asthma trigger - exercise | Respiratory | Read |
| 1J70. | Suspected asthma | Respiratory | Read |
| 1O2.. | Asthma confirmed | Respiratory | Read |
| 663.. | Respiratory disease monitoring | Respiratory | Read |
| 663d. | Emergency asthma admission since last appointment | Respiratory | Read |
| 663e. | Asthma restricts exercise | Respiratory | Read |
| 663f. | Asthma never restricts exercise | Respiratory | Read |
| 663j. | Asthma - currently active | Respiratory | Read |
| 663J. | Airways obstruction reversible | Respiratory | Read |
| 663m. | Asthma accident and emergency attendance since last visit | Respiratory | Read |
| 663n. | Asthma treatment compliance satisfactory | Respiratory | Read |
| 663N. | Asthma disturbing sleep | Respiratory | Read |
| 663N0 | Asthma causing night waking | Respiratory | Read |
| 663N1 | Asthma disturbs sleep weekly | Respiratory | Read |
| 663N2 | Asthma disturbs sleep frequently | Respiratory | Read |
| 663O. | Asthma not disturbing sleep | Respiratory | Read |
| 663O0 | Asthma never disturbs sleep | Respiratory | Read |
| 663p. | Asthma treatment compliance unsatisfactory | Respiratory | Read |
| 663P. | Asthma limiting activities | Respiratory | Read |
| 663P0 | Asthma limits activities 1 to 2 times per month | Respiratory | Read |
| 663P1 | Asthma limits activities 1 to 2 times per week | Respiratory | Read |
| 663P2 | Asthma limits activities most days | Respiratory | Read |
| 663q. | Asthma daytime symptoms | Respiratory | Read |
| 663Q. | Asthma not limiting activities | Respiratory | Read |
| 663r. | Asthma causes night symptoms 1 to 2 times per month | Respiratory | Read |
| 663s. | Asthma never causes daytime symptoms | Respiratory | Read |
| 663t. | Asthma causes daytime symptoms 1 to 2 times per month | Respiratory | Read |
| 663u. | Asthma causes daytime symptoms 1 to 2 times per week | Respiratory | Read |
| 663U. | Asthma management plan given | Respiratory | Read |
| 663v. | Asthma causes daytime symptoms most days | Respiratory | Read |
| 663V. | Asthma severity | Respiratory | Read |
| 663V0 | Occasional asthma | Respiratory | Read |
| 663V1 | Mild asthma | Respiratory | Read |
| 663V2 | Moderate asthma | Respiratory | Read |
| 663V3 | Severe asthma | Respiratory | Read |
| 663w. | Asthma limits walking up hills or stairs | Respiratory | Read |
| 663W. | Asthma prophylactic medication used | Respiratory | Read |
| 663x. | Asthma limits walking on the flat | Respiratory | Read |
| 663y. | Number of asthma exacerbations in past year | Respiratory | Read |
| 66Y9. | Step up change in asthma management plan | Respiratory | Read |
| 66YC. | Absent from work or school due to asthma | Respiratory | Read |
| 66YK. | Asthma follow-up | Respiratory | Read |
| 66YP. | Asthma night-time symptoms | Respiratory | Read |
| 66Yq. | Asthma causes night time symptoms 1 to 2 times per week | Respiratory | Read |
| 66Yr. | Asthma causes symptoms most nights | Respiratory | Read |
| 8H2P. | Emergency admission, asthma | Respiratory | Read |
| H3120 | Chronic asthmatic bronchitis | Respiratory | Read |
| H33.. | Asthma | Respiratory | Read |
| H330. | Extrinsic (atopic) asthma | Respiratory | Read |
| H3300 | Extrinsic asthma without status asthmaticus | Respiratory | Read |
| H3301 | Extrinsic asthma with status asthmaticus | Respiratory | Read |
| H330z | Extrinsic asthma NOS | Respiratory | Read |
| H331. | Intrinsic asthma | Respiratory | Read |
| H3310 | Intrinsic asthma without status asthmaticus | Respiratory | Read |
| H3311 | Intrinsic asthma with status asthmaticus | Respiratory | Read |
| H331z | Intrinsic asthma NOS | Respiratory | Read |
| H332. | Mixed asthma | Respiratory | Read |
| H333. | Acute exacerbation of asthma | Respiratory | Read |
| H334. | Brittle asthma | Respiratory | Read |
| H335. | Chronic asthma with fixed airflow obstruction | Respiratory | Read |
| H33z. | Asthma unspecified | Respiratory | Read |
| H33z0 | Status asthmaticus NOS | Respiratory | Read |
| H33z1 | Asthma attack | Respiratory | Read |
| H33z2 | Late-onset asthma | Respiratory | Read |
| H33zz | Asthma NOS | Respiratory | Read |
| H35y7 | Wood asthma | Respiratory | Read |
| H47y0 | Detergent asthma | Respiratory | Read |
| 1737. | Wheezing | Respiratory | Read |
| 2326. | O/E - expiratory wheeze | Respiratory | Read |
| 173B. | Nocturnal cough / wheeze | Respiratory | Read |
| 173e. | Viral wheeze | Respiratory | Read |
| 23D2. | O/E - rhonchi present | Respiratory | Read |
| 663a. | Oral steroids used since last appointment | Respiratory | Read |
| 663b. | Home nebuliser used since last appointment | Respiratory | Read |
| 663c. | Nebulisation since last appointment | Respiratory | Read |
| 663F. | Oral steroids started | Respiratory | Read |
| 663G. | Oral steroids stopped | Respiratory | Read |
| 663g1 | Using inhaled steroids - normal dose | Respiratory | Read |
| 663g2 | Using inhaled steroids - high dose | Respiratory | Read |
| 663g3 | Increases inhaled steroids appropriately | Respiratory | Read |
| 663g4 | Using inhaled steroids - low dose | Respiratory | Read |
| 663l. | Spacer device in use | Respiratory | Read |
| 663L. | Bronchodilators used more than once daily | Respiratory | Read |
| 663M. | Bronchodilators used a maximum of once daily | Respiratory | Read |
| 663S. | Peak flow meter at home | Respiratory | Read |
| 663T. | No peak flow meter at home | Respiratory | Read |
| 663Y. | Steroid dose inhaled daily | Respiratory | Read |
| 663z. | Number of times bronchodilator used in one week | Respiratory | Read |
| 663Z0 | Bronchodilator used infrequently | Respiratory | Read |
| 663Z1 | Bronchodilator not used in last month | Respiratory | Read |
| H3121 | Emphysematous bronchitis | Respiratory | Read |
| H32.. | Emphysema | Respiratory | Read |
| H4640 | Chronic emphysema due to chemical fumes | Respiratory | Read |
| H581. | Interstitial emphysema | Respiratory | Read |
| H5y16 | Bronchospasm | Respiratory | Read |
| R0609 | [D]Wheezing | Respiratory | Read |
| R060E | [D]Mild wheeze | Respiratory | Read |
| R060F | [D]Moderate wheeze | Respiratory | Read |
| R060G | [D]Severe wheeze | Respiratory | Read |
| R060H | [D]Very severe wheeze | Respiratory | Read |
| H66. | Suppurative and unspecified otitis media | Respiratory | ICD-10 |
| H660 | Acute suppurative otitis media | Respiratory | ICD-10 |
| H661 | Chronic tubotympanic suppurative otitis media | Respiratory | ICD-10 |
| H662 | Chronic atticoantral suppurative otitis media | Respiratory | ICD-10 |
| H663 | Other chronic suppurative otitis media | Respiratory | ICD-10 |
| H664 | Suppurative otitis media, unspecified | Respiratory | ICD-10 |
| H669 | Otitis media, unspecified | Respiratory | ICD-10 |
| H67. | Otitis media in diseases classified elsewhere | Respiratory | ICD-10 |
| H670 | Otitis media in bacterial diseases classified elsewhere | Respiratory | ICD-10 |
| H671 | Otitis media in viral diseases classified elsewhere | Respiratory | ICD-10 |
| H678 | Otitis media in other diseases classified elsewhere | Respiratory | ICD-10 |
| 14B3. | H/O: chr.obstr. airway disease | Respiratory | Read |
| 663K. | Airways obstructn irreversible | Respiratory | Read |
| 66YL. | Chronic obstructive pulmonary disease follow-up | Respiratory | Read |
| H31.. | Chronic bronchitis | Respiratory | Read |
| H36.. | Mild chronic obstructive pulmonary disease | Respiratory | Read |
| H37.. | Moderate chronic obstructive pulmonary disease | Respiratory | Read |
| H38.. | Severe chronic obstructive pulmonary disease | Respiratory | Read |
| H39.. | Very severe chronic obstructive pulmonary disease | Respiratory | Read |
| H3A.. | End stage chronic obstructive airways disease | Respiratory | Read |
| H3y.. | Other specified chronic obstructive airways disease | Respiratory | Read |
| H3z.. | Chronic obstructive airways disease NOS | Respiratory | Read |
| J00X | Acute nasopharyngitis [common cold] | Respiratory | ICD-10 |
| J01. | Acute sinusitis | Respiratory | ICD-10 |
| J010 | Acute maxillary sinusitis | Respiratory | ICD-10 |
| J011 | Acute frontal sinusitis | Respiratory | ICD-10 |
| J012 | Acute ethmoidal sinusitis | Respiratory | ICD-10 |
| J013 | Acute sphenoidal sinusitis | Respiratory | ICD-10 |
| J014 | Acute pansinusitis | Respiratory | ICD-10 |
| J018 | Other acute sinusitis | Respiratory | ICD-10 |
| J019 | Acute sinusitis, unspecified | Respiratory | ICD-10 |
| J02. | Acute pharyngitis | Respiratory | ICD-10 |
| J020 | Streptococcal pharyngitis | Respiratory | ICD-10 |
| J028 | Acute pharyngitis due to other specified organisms | Respiratory | ICD-10 |
| J029 | Acute pharyngitis, unspecified | Respiratory | ICD-10 |
| J03. | Acute tonsillitis | Respiratory | ICD-10 |
| J030 | Streptococcal tonsillitis | Respiratory | ICD-10 |
| J038 | Acute tonsillitis due to other specified organisms | Respiratory | ICD-10 |
| J039 | Acute tonsillitis, unspecified | Respiratory | ICD-10 |
| J04. | Acute laryngitis and tracheitis | Respiratory | ICD-10 |
| J040 | Acute laryngitis | Respiratory | ICD-10 |
| J041 | Acute tracheitis | Respiratory | ICD-10 |
| J042 | Acute laryngotracheitis | Respiratory | ICD-10 |
| J05. | Acute obstructive laryngitis [croup] and epiglottitis | Respiratory | ICD-10 |
| J050 | Acute obstructive laryngitis [croup] | Respiratory | ICD-10 |
| J051 | Acute epiglottitis | Respiratory | ICD-10 |
| J06. | Acute upper respiratory infections of multiple and unspecified sites | Respiratory | ICD-10 |
| J060 | Acute laryngopharyngitis | Respiratory | ICD-10 |
| J068 | Other acute upper respiratory infections of multiple sites | Respiratory | ICD-10 |
| J069 | Acute upper respiratory infection, unspecified | Respiratory | ICD-10 |
| J09X | Influenza due to identified avian influenza virus | Respiratory | ICD-10 |
| J10. | Influenza due to other identified influenza virus | Respiratory | ICD-10 |
| J100 | Influenza with pneumonia, other influenza virus identified | Respiratory | ICD-10 |
| J101 | Influenza with other respiratory manifestations, other influenza virus identified | Respiratory | ICD-10 |
| J108 | Influenza with other manifestations, other influenza virus identified | Respiratory | ICD-10 |
| J11. | Influenza, virus not identified | Respiratory | ICD-10 |
| J110 | Influenza with pneumonia, virus not identified | Respiratory | ICD-10 |
| J111 | Influenza with other respiratory manifestations, virus not identified | Respiratory | ICD-10 |
| J118 | Influenza with other manifestations, virus not identified | Respiratory | ICD-10 |
| J12. | Viral pneumonia, not elsewhere classified | Respiratory | ICD-10 |
| J120 | Adenoviral pneumonia | Respiratory | ICD-10 |
| J121 | Respiratory syncytial virus pneumonia | Respiratory | ICD-10 |
| J122 | Parainfluenza virus pneumonia | Respiratory | ICD-10 |
| J123 | Human metapneumovirus pneumonia | Respiratory | ICD-10 |
| J128 | Other viral pneumonia | Respiratory | ICD-10 |
| J129 | Viral pneumonia, unspecified | Respiratory | ICD-10 |
| J13X | Pneumonia due to Streptococcus pneumoniae | Respiratory | ICD-10 |
| J14X | Pneumonia due to Haemophilus influenzae | Respiratory | ICD-10 |
| J15. | Bacterial pneumonia, not elsewhere classified | Respiratory | ICD-10 |
| J150 | Pneumonia due to Klebsiella pneumoniae | Respiratory | ICD-10 |
| J151 | Pneumonia due to Pseudomonas | Respiratory | ICD-10 |
| J152 | Pneumonia due to staphylococcus | Respiratory | ICD-10 |
| J153 | Pneumonia due to streptococcus, group B | Respiratory | ICD-10 |
| J154 | Pneumonia due to other streptococci | Respiratory | ICD-10 |
| J155 | Pneumonia due to Escherichia coli | Respiratory | ICD-10 |
| J156 | Pneumonia due to other aerobic Gram-negative bacteria | Respiratory | ICD-10 |
| J157 | Pneumonia due to Mycoplasma pneumoniae | Respiratory | ICD-10 |
| J158 | Other bacterial pneumonia | Respiratory | ICD-10 |
| J159 | Bacterial pneumonia, unspecified | Respiratory | ICD-10 |
| J16. | Pneumonia due to other infectious organisms, not elsewhere classified | Respiratory | ICD-10 |
| J160 | Chlamydial pneumonia | Respiratory | ICD-10 |
| J168 | Pneumonia due to other specified infectious organisms | Respiratory | ICD-10 |
| J17. | Pneumonia in diseases classified elsewhere | Respiratory | ICD-10 |
| J170 | Pneumonia in bacterial diseases classified elsewhere | Respiratory | ICD-10 |
| J171 | Pneumonia in viral diseases classified elsewhere | Respiratory | ICD-10 |
| J172 | Pneumonia in mycoses | Respiratory | ICD-10 |
| J173 | Pneumonia in parasitic diseases | Respiratory | ICD-10 |
| J178 | Pneumonia in other diseases classified elsewhere | Respiratory | ICD-10 |
| J18. | Pneumonia, organism unspecified | Respiratory | ICD-10 |
| J180 | Bronchopneumonia, unspecified | Respiratory | ICD-10 |
| J181 | Lobar pneumonia, unspecified | Respiratory | ICD-10 |
| J182 | Hypostatic pneumonia, unspecified | Respiratory | ICD-10 |
| J188 | Other pneumonia, organism unspecified | Respiratory | ICD-10 |
| J189 | Pneumonia, unspecified | Respiratory | ICD-10 |
| 1825. | Pleuritic pain | Respiratory | Read |
| 1827. | Painful breathing -pleurodynia | Respiratory | Read |
| 23D5. | O/E - pleural friction rub | Respiratory | Read |
| 4JU9. | Respiratory syncytial virus A detected | Respiratory | Read |
| 4JUA. | Respiratory syncytial virus B detected | Respiratory | Read |
| 4JUB. | Respiratory syncytial virus untyped strain detected | Respiratory | Read |
| 4JUK. | Mycoplasma pneumoniae detected | Respiratory | Read |
| A0222 | Salmonella pneumonia | Respiratory | Read |
| A521. | Varicella pneumonitis | Respiratory | Read |
| A54x4 | Herpes simplex pneumonia | Respiratory | Read |
| A551. | Postmeasles pneumonia | Respiratory | Read |
| A730. | Ornithosis with pneumonia | Respiratory | Read |
| A741. | Epidemic pleurodynia | Respiratory | Read |
| A7850 | Cytomegaloviral pneumonitis | Respiratory | Read |
| A79A. | Respiratory syncytial virus infection | Respiratory | Read |
| A7y02 | Resp syncytial virus as cause of dis class to other chapters | Respiratory | Read |
| AyuKN | [X]Resp syncytial virus/cause/diseases classfd/oth chapters | Respiratory | Read |
| H06.. | Acute bronchitis and bronchiolitis | Respiratory | Read |
| H060. | Acute bronchitis | Respiratory | Read |
| H0600 | Acute fibrinous bronchitis | Respiratory | Read |
| H0601 | Acute membranous bronchitis | Respiratory | Read |
| H0602 | Acute pseudomembranous bronchitis | Respiratory | Read |
| H0603 | Acute purulent bronchitis | Respiratory | Read |
| H0604 | Acute croupous bronchitis | Respiratory | Read |
| H0605 | Acute tracheobronchitis | Respiratory | Read |
| H0606 | Acute pneumococcal bronchitis | Respiratory | Read |
| H0607 | Acute streptococcal bronchitis | Respiratory | Read |
| H0608 | Acute haemophilus influenzae bronchitis | Respiratory | Read |
| H0609 | Acute neisseria catarrhalis bronchitis | Respiratory | Read |
| H060A | Acute bronchitis due to mycoplasma pneumoniae | Respiratory | Read |
| H060B | Acute bronchitis due to coxsackievirus | Respiratory | Read |
| H060C | Acute bronchitis due to parainfluenza virus | Respiratory | Read |
| H060D | Acute bronchitis due to respiratory syncytial virus | Respiratory | Read |
| H060E | Acute bronchitis due to rhinovirus | Respiratory | Read |
| H060F | Acute bronchitis due to echovirus | Respiratory | Read |
| H060v | Subacute bronchitis unspecified | Respiratory | Read |
| H060w | Acute viral bronchitis unspecified | Respiratory | Read |
| H060x | Acute bacterial bronchitis unspecified | Respiratory | Read |
| H060z | Acute bronchitis NOS | Respiratory | Read |
| H061. | Acute bronchiolitis | Respiratory | Read |
| H0610 | Acute capillary bronchiolitis | Respiratory | Read |
| H0611 | Acute obliterating bronchiolitis | Respiratory | Read |
| H0612 | Acute bronchiolitis with bronchospasm | Respiratory | Read |
| H0613 | Acute exudative bronchiolitis | Respiratory | Read |
| H0614 | Obliterating fibrous bronchiolitis | Respiratory | Read |
| H0615 | Acute bronchiolitis due to respiratory syncytial virus | Respiratory | Read |
| H0616 | Acute bronchiolitis due to other specified organisms | Respiratory | Read |
| H0617 | Acute bronchiolitis due to human metapneumovirus | Respiratory | Read |
| H061z | Acute bronchiolitis NOS | Respiratory | Read |
| H062. | Acute lower respiratory tract infection | Respiratory | Read |
| H06z. | Acute bronchitis or bronchiolitis NOS | Respiratory | Read |
| H06z1 | Lower resp tract infection | Respiratory | Read |
| H07.. | Chest cold | Respiratory | Read |
| H2... | Pneumonia and influenza | Respiratory | Read |
| H20.. | Viral pneumonia | Respiratory | Read |
| H200. | Pneumonia due to adenovirus | Respiratory | Read |
| H201. | Pneumonia due to respiratory syncytial virus | Respiratory | Read |
| H202. | Pneumonia due to parainfluenza virus | Respiratory | Read |
| H203. | Pneumonia due to human metapneumovirus | Respiratory | Read |
| H20y. | Viral pneumonia NEC | Respiratory | Read |
| H20y0 | Severe acute respiratory syndrome | Respiratory | Read |
| H20z. | Viral pneumonia NOS | Respiratory | Read |
| H21.. | Lobar (pneumococcal) pneumonia | Respiratory | Read |
| H22.. | Other bacterial pneumonia | Respiratory | Read |
| H23.. | Pneumonia due to other specified organisms | Respiratory | Read |
| H231. | Pneumonia due to mycoplasma pneumoniae | Respiratory | Read |
| H232. | Pneumonia due to pleuropneumonia like organisms | Respiratory | Read |
| H233. | Chlamydial pneumonia | Respiratory | Read |
| H23z. | Pneumonia due to specified organism NOS | Respiratory | Read |
| H24.. | Pneumonia with infectious diseases EC | Respiratory | Read |
| H240. | Pneumonia with measles | Respiratory | Read |
| H241. | Pneumonia with cytomegalic inclusion disease | Respiratory | Read |
| H243. | Pneumonia with whooping cough | Respiratory | Read |
| H24y. | Pneumonia with other infectious diseases EC | Respiratory | Read |
| H24y7 | Pneumonia with varicella | Respiratory | Read |
| H24yz | Pneumonia with other infectious diseases EC NOS | Respiratory | Read |
| H24z. | Pneumonia with infectious diseases EC NOS | Respiratory | Read |
| H25.. | Bronchopneumonia due to unspecified organism | Respiratory | Read |
| H26.. | Pneumonia due to unspecified organism | Respiratory | Read |
| H260. | Lobar pneumonia due to unspecified organism | Respiratory | Read |
| H261. | Basal pneumonia due to unspecified organism | Respiratory | Read |
| H263. | Pneumonitis, unspecified | Respiratory | Read |
| H270. | Influenza with pneumonia | Respiratory | Read |
| H2700 | Influenza with bronchopneumonia | Respiratory | Read |
| H2701 | Influenza with pneumonia, influenza virus identified | Respiratory | Read |
| H270z | Influenza with pneumonia NOS | Respiratory | Read |
| H28.. | Atypical pneumonia | Respiratory | Read |
| H2y.. | Other specified pneumonia or influenza | Respiratory | Read |
| H2z.. | Pneumonia or influenza NOS | Respiratory | Read |
| H30.. | Bronchitis unspecified | Respiratory | Read |
| H300. | Tracheobronchitis NOS | Respiratory | Read |
| H301. | Laryngotracheobronchitis | Respiratory | Read |
| H302. | Wheezy bronchitis | Respiratory | Read |
| H30z. | Bronchitis NOS | Respiratory | Read |
| H357. | Ventilation pneumonitis | Respiratory | Read |
| H5010 | Pleural abscess | Respiratory | Read |
| H5012 | Pleural empyema | Respiratory | Read |
| H5014 | Purulent pleurisy | Respiratory | Read |
| H51.. | Pleurisy | Respiratory | Read |
| H510. | Pleurisy without effusion or active tuberculosis | Respiratory | Read |
| H5103 | Acute dry pleurisy | Respiratory | Read |
| H5104 | Diaphragmatic pleurisy | Respiratory | Read |
| H5105 | Basal pleurisy | Respiratory | Read |
| H5109 | Pneumococcal pleurisy | Respiratory | Read |
| H510A | Staphylococcal pleurisy | Respiratory | Read |
| H510B | Streptococcal pleurisy | Respiratory | Read |
| H510z | Pleurisy without effusion or active tuberculosis NOS | Respiratory | Read |
| H511. | Bacterial pleurisy with effusion | Respiratory | Read |
| H5110 | Pneumococcal pleurisy with effusion | Respiratory | Read |
| H5111 | Staphylococcal pleurisy with effusion | Respiratory | Read |
| H511z | Bacterial pleurisy with effusion NOS | Respiratory | Read |
| H51y. | Other pleural effusion excluding mention of tuberculosis | Respiratory | Read |
| H5303 | Abscess of lung with pneumonia | Respiratory | Read |
| H564. | Bronchiolitis obliterans organising pneumonia | Respiratory | Read |
| H56y1 | Interstitial pneumonia | Respiratory | Read |
| Hyu08 | [X]Other viral pneumonia | Respiratory | Read |
| Hyu0A | [X]Other bacterial pneumonia | Respiratory | Read |
| Hyu0B | [X]Pneumonia due to other specified infectious organisms | Respiratory | Read |
| Hyu0D | [X]Pneumonia in viral diseases classified elsewhere | Respiratory | Read |
| Hyu0H | [X]Other pneumonia, organism unspecified | Respiratory | Read |
| Hyu1. | [X]Other acute lower respiratory infections | Respiratory | Read |
| Hyu10 | [X]Acute bronchitis due to other specified organisms | Respiratory | Read |
| Hyu11 | [X]Acute bronchiolitis due to other specified organisms | Respiratory | Read |
| R0654 | [D]Pleuritic pain | Respiratory | Read |
| R0655 | [D]Pleurodynia | Respiratory | Read |
| J20. | Acute bronchitis | Respiratory | ICD-10 |
| J200 | Acute bronchitis due to Mycoplasma pneumoniae | Respiratory | ICD-10 |
| J201 | Acute bronchitis due to Haemophilus influenzae | Respiratory | ICD-10 |
| J202 | Acute bronchitis due to streptococcus | Respiratory | ICD-10 |
| J203 | Acute bronchitis due to coxsackievirus | Respiratory | ICD-10 |
| J204 | Acute bronchitis due to parainfluenza virus | Respiratory | ICD-10 |
| J205 | Acute bronchitis due to respiratory syncytial virus | Respiratory | ICD-10 |
| J206 | Acute bronchitis due to rhinovirus | Respiratory | ICD-10 |
| J207 | Acute bronchitis due to echovirus | Respiratory | ICD-10 |
| J208 | Acute bronchitis due to other specified organisms | Respiratory | ICD-10 |
| J209 | Acute bronchitis, unspecified | Respiratory | ICD-10 |
| J21. | Acute bronchiolitis | Respiratory | ICD-10 |
| J210 | Acute bronchiolitis due to respiratory syncytial virus | Respiratory | ICD-10 |
| J211 | Acute bronchiolitis due to human metapneumovirus | Respiratory | ICD-10 |
| J218 | Acute bronchiolitis due to other specified organisms | Respiratory | ICD-10 |
| J219 | Acute bronchiolitis, unspecified | Respiratory | ICD-10 |
| J22X | Unspecified acute lower respiratory infection | Respiratory | ICD-10 |
| 1656. | Feverish cold | Respiratory | Read |
| 75311 | Drainage of peritonsillar abscess | Respiratory | Read |
| 16L.. | Influenza-like symptoms | Respiratory | Read |
| 1C3.. | Earache symptoms | Respiratory | Read |
| 1C32. | Unilateral earache | Respiratory | Read |
| 1C33. | Bilateral earache | Respiratory | Read |
| 1C3Z. | Earache symptom NOS | Respiratory | Read |
| 1C9.. | Sore throat symptom | Respiratory | Read |
| 1C92. | Has a sore throat | Respiratory | Read |
| 1C93. | Persistent sore throat | Respiratory | Read |
| 1C9Z. | Sore throat symptom NOS | Respiratory | Read |
| 1CB3. | Throat pain | Respiratory | Read |
| 2D95. | O/E - tympanic membrane red | Respiratory | Read |
| 2DB6. | O/E - follicular tonsillitis | Respiratory | Read |
| 2DB7. | O/E - exudate on tonsils | Respiratory | Read |
| 2DC1. | O/E - pharynx hyperaemic | Respiratory | Read |
| 2DC2. | O/E - granular pharyngitis | Respiratory | Read |
| 2DC3. | Inflamed throat | Respiratory | Read |
| 4J3L. | Influenza A virus H1N1 subtype detected | Respiratory | Read |
| 4JF40 | Throat swab culture positive | Respiratory | Read |
| 4JU0. | Influenza H1 virus detected | Respiratory | Read |
| 4JU1. | Influenza H2 virus detected | Respiratory | Read |
| 4JU2. | Influenza H3 virus detected | Respiratory | Read |
| 4JU3. | Influenza H5 virus detected | Respiratory | Read |
| 4JU4. | Influenza A virus, other or untyped strain detected | Respiratory | Read |
| 4JU5. | Influenza B virus detected | Respiratory | Read |
| 4JUE. | Human rhinovirus detected | Respiratory | Read |
| 4JUF. | Human parainfluenza virus detected | Respiratory | Read |
| 65VA. | Notification of whooping cough | Respiratory | Read |
| A32.. | Diphtheria | Respiratory | Read |
| A320. | Faucial diphtheria | Respiratory | Read |
| A321. | Nasopharyngeal diphtheria | Respiratory | Read |
| A322. | Anterior nasal diphtheria | Respiratory | Read |
| A323. | Laryngeal diphtheria | Respiratory | Read |
| A32yz | Other specified diphtheria NOS | Respiratory | Read |
| A32z. | Diphtheria NOS | Respiratory | Read |
| A33.. | Whooping cough | Respiratory | Read |
| A330. | Bordetella pertussis | Respiratory | Read |
| A331. | Bordetella parapertussis | Respiratory | Read |
| A33y. | Whooping cough - other specified organism | Respiratory | Read |
| A33y0 | Bordetella bronchiseptica | Respiratory | Read |
| A33yz | Other whooping cough NOS | Respiratory | Read |
| A33z. | Whooping cough NOS | Respiratory | Read |
| A34.. | Streptococcal sore throat and scarlatina | Respiratory | Read |
| A340. | Streptococcal sore throat | Respiratory | Read |
| A3400 | Streptococcal angina | Respiratory | Read |
| A3401 | Streptococcal laryngitis | Respiratory | Read |
| A3402 | Streptococcal pharyngitis | Respiratory | Read |
| A3403 | Streptococcal tonsillitis | Respiratory | Read |
| A340z | Streptococcal sore throat NOS | Respiratory | Read |
| A341. | Scarlet fever - scarlatina | Respiratory | Read |
| A34z. | Streptococcal sore throat with scarlatina NOS | Respiratory | Read |
| A552. | Postmeasles otitis media | Respiratory | Read |
| A793. | Rhinovirus | Respiratory | Read |
| Ayu38 | [X]Diphtheria, unspecified | Respiratory | Read |
| Ayu3A | [X]Whooping cough, unspecified | Respiratory | Read |
| F51.. | Nonsuppurative otitis media + eustachian tube disorders | Respiratory | Read |
| F510. | Acute non suppurative otitis media | Respiratory | Read |
| F5100 | Acute otitis media with effusion | Respiratory | Read |
| F5101 | Acute serous otitis media | Respiratory | Read |
| F5102 | Acute mucoid otitis media | Respiratory | Read |
| F5103 | Acute sanguinous otitis media | Respiratory | Read |
| F5104 | Acute allergic serous otitis media | Respiratory | Read |
| F5106 | Acute allergic sanguinous otitis media | Respiratory | Read |
| F510z | Acute nonsuppurative otitis media NOS | Respiratory | Read |
| F514. | Unspecified nonsuppurative otitis media | Respiratory | Read |
| F5141 | Serous otitis media NOS | Respiratory | Read |
| F5142 | Catarrhal otitis media NOS | Respiratory | Read |
| F5143 | Mucoid otitis media NOS | Respiratory | Read |
| F514z | Nonsuppurative otitis media NOS | Respiratory | Read |
| F515. | Eustachian tube salpingitis | Respiratory | Read |
| F5150 | Unspecified eustachian tube salpingitis | Respiratory | Read |
| F5151 | Acute eustachian tube salpingitis | Respiratory | Read |
| F515z | Eustachian tube salpingitis NOS | Respiratory | Read |
| F52.. | Suppurative and unspecified otitis media | Respiratory | Read |
| F520. | Acute suppurative otitis media | Respiratory | Read |
| F5200 | Acute suppurative otitis media tympanic membrane intact | Respiratory | Read |
| F5201 | Acute suppurative otitis media tympanic membrane ruptured | Respiratory | Read |
| F5203 | Acute suppurative otitis media due to disease EC | Respiratory | Read |
| F520z | Acute suppurative otitis media NOS | Respiratory | Read |
| F524. | Purulent otitis media NOS | Respiratory | Read |
| F5240 | Bilateral suppurative otitis media | Respiratory | Read |
| F525. | Recurrent acute otitis media | Respiratory | Read |
| F526. | Acute left otitis media | Respiratory | Read |
| F527. | Acute right otitis media | Respiratory | Read |
| F528. | Acute bilateral otitis media | Respiratory | Read |
| F52z. | Otitis media NOS | Respiratory | Read |
| F53.. | Mastoiditis and related conditions | Respiratory | Read |
| F530. | Acute mastoiditis | Respiratory | Read |
| F5300 | Acute mastoiditis without complications | Respiratory | Read |
| F5301 | Subperiosteal mastoid abscess | Respiratory | Read |
| F5302 | Gradenigo's syndrome | Respiratory | Read |
| F5303 | Acute mastoiditis with other complication | Respiratory | Read |
| F530z | Acute mastoiditis NOS | Respiratory | Read |
| F540. | Acute myringitis without otitis media | Respiratory | Read |
| F5401 | Unspecified acute tympanitis | Respiratory | Read |
| F5402 | Bullous myringitis | Respiratory | Read |
| F540z | Acute myringitis NOS | Respiratory | Read |
| F587. | Otalgia | Respiratory | Read |
| F5870 | Unspecified otalgia | Respiratory | Read |
| F5871 | Otogenic pain | Respiratory | Read |
| F587z | Otalgia NOS | Respiratory | Read |
| FyuP0 | [X]Other acute nonsuppurative otitis media | Respiratory | Read |
| FyuP3 | [X]Otitis media in bacterial diseases classified elsewhere | Respiratory | Read |
| FyuP4 | [X]Otitis media in viral diseases classified elsewhere | Respiratory | Read |
| FyuP5 | [X]Otitis media in other diseases classified elsewhere | Respiratory | Read |
| FyuP7 | [X]Other mastoiditis and related conditions | Respiratory | Read |
| H00.. | Acute nasopharyngitis | Respiratory | Read |
| H01.. | Acute sinusitis | Respiratory | Read |
| H010. | Acute maxillary sinusitis | Respiratory | Read |
| H011. | Acute frontal sinusitis | Respiratory | Read |
| H012. | Acute ethmoidal sinusitis | Respiratory | Read |
| H013. | Acute sphenoidal sinusitis | Respiratory | Read |
| H014. | Acute rhinosinusitis | Respiratory | Read |
| H01y. | Other acute sinusitis | Respiratory | Read |
| H01y0 | Acute pansinusitis | Respiratory | Read |
| H01yz | Other acute sinusitis NOS | Respiratory | Read |
| H01z. | Acute sinusitis NOS | Respiratory | Read |
| H02.. | Acute pharyngitis | Respiratory | Read |
| H020. | Acute gangrenous pharyngitis | Respiratory | Read |
| H021. | Acute phlegmonous pharyngitis | Respiratory | Read |
| H022. | Acute ulcerative pharyngitis | Respiratory | Read |
| H023. | Acute bacterial pharyngitis | Respiratory | Read |
| H0230 | Acute pneumococcal pharyngitis | Respiratory | Read |
| H0231 | Acute staphylococcal pharyngitis | Respiratory | Read |
| H023z | Acute bacterial pharyngitis NOS | Respiratory | Read |
| H024. | Acute viral pharyngitis | Respiratory | Read |
| H02z. | Acute pharyngitis NOS | Respiratory | Read |
| H03.. | Acute tonsillitis | Respiratory | Read |
| H030. | Acute erythematous tonsillitis | Respiratory | Read |
| H031. | Acute follicular tonsillitis | Respiratory | Read |
| H032. | Acute ulcerative tonsillitis | Respiratory | Read |
| H033. | Acute catarrhal tonsillitis | Respiratory | Read |
| H034. | Acute gangrenous tonsillitis | Respiratory | Read |
| H035. | Acute bacterial tonsillitis | Respiratory | Read |
| H0350 | Acute pneumococcal tonsillitis | Respiratory | Read |
| H0351 | Acute staphylococcal tonsillitis | Respiratory | Read |
| H035z | Acute bacterial tonsillitis NOS | Respiratory | Read |
| H036. | Acute viral tonsillitis | Respiratory | Read |
| H037. | Recurrent acute tonsillitis | Respiratory | Read |
| H03z. | Acute tonsillitis NOS | Respiratory | Read |
| H04.. | Acute laryngitis and tracheitis | Respiratory | Read |
| H040. | Acute laryngitis | Respiratory | Read |
| H0400 | Acute oedematous laryngitis | Respiratory | Read |
| H0401 | Acute ulcerative laryngitis | Respiratory | Read |
| H0402 | Acute catarrhal laryngitis | Respiratory | Read |
| H0403 | Acute phlegmonous laryngitis | Respiratory | Read |
| H0404 | Acute haemophilus influenzae laryngitis | Respiratory | Read |
| H0405 | Acute pneumococcal laryngitis | Respiratory | Read |
| H0406 | Acute suppurative laryngitis | Respiratory | Read |
| H040w | Acute viral laryngitis unspecified | Respiratory | Read |
| H040x | Acute bacterial laryngitis unspecified | Respiratory | Read |
| H040z | Acute laryngitis NOS | Respiratory | Read |
| H042. | Acute laryngotracheitis | Respiratory | Read |
| H0420 | Acute laryngotracheitis without obstruction | Respiratory | Read |
| H0421 | Acute laryngotracheitis with obstruction | Respiratory | Read |
| H042z | Acute laryngotracheitis NOS | Respiratory | Read |
| H043. | Acute epiglottitis (non strep) | Respiratory | Read |
| H0430 | Acute epiglottitis without obstruction | Respiratory | Read |
| H0431 | Acute epiglottitis with obstruction | Respiratory | Read |
| H0432 | Acute obstructive laryngitis | Respiratory | Read |
| H043z | Acute epiglottitis NOS | Respiratory | Read |
| H044. | Croup | Respiratory | Read |
| H04z. | Acute laryngitis and tracheitis NOS | Respiratory | Read |
| H05.. | Other acute upper respiratory infections | Respiratory | Read |
| H050. | Acute laryngopharyngitis | Respiratory | Read |
| H051. | Acute upper respiratory tract infection | Respiratory | Read |
| H052. | Pharyngotracheitis | Respiratory | Read |
| H053. | Tracheopharyngitis | Respiratory | Read |
| H054. | Recurrent upper respiratory tract infection | Respiratory | Read |
| H055. | Pharyngolaryngitis | Respiratory | Read |
| H05y. | Other upper respiratory infections of multiple sites | Respiratory | Read |
| H05z. | Upper respiratory infection NOS | Respiratory | Read |
| H1201 | Chronic catarrhal rhinitis | Respiratory | Read |
| H1211 | Atrophic pharyngitis | Respiratory | Read |
| H1212 | Granular pharyngitis | Respiratory | Read |
| H1213 | Hypertrophic pharyngitis | Respiratory | Read |
| H130. | Chronic maxillary sinusitis | Respiratory | Read |
| H131. | Chronic frontal sinusitis | Respiratory | Read |
| H135. | Recurrent sinusitis | Respiratory | Read |
| H13y1 | Pansinusitis | Respiratory | Read |
| H141. | Tonsil and/or adenoid hypertrophy | Respiratory | Read |
| H15.. | Peritonsillar abscess - quinsy | Respiratory | Read |
| H1y1z | Nasal cavity and sinus disease NOS | Respiratory | Read |
| H1y22 | Parapharyngeal abscess | Respiratory | Read |
| H1y23 | Retropharyngeal abscess | Respiratory | Read |
| H1y26 | Pharynx or nasopharynx abscess | Respiratory | Read |
| H27.. | Influenza | Respiratory | Read |
| H2710 | Influenza with laryngitis | Respiratory | Read |
| H2711 | Influenza with pharyngitis | Respiratory | Read |
| H27z. | Influenza NOS | Respiratory | Read |
| H29.. | Avian influenza | Respiratory | Read |
| H2A.. | Influenza due to Influenza A virus subtype H1N1 | Respiratory | Read |
| Hyu0. | [X]Acute upper respiratory infections | Respiratory | Read |
| Hyu00 | [X]Other acute sinusitis | Respiratory | Read |
| Hyu01 | [X]Acute pharyngitis due to other specified organisms | Respiratory | Read |
| Hyu02 | [X]Acute tonsillitis due to other specified organisms | Respiratory | Read |
| Hyu04 | [X]Flu+oth respiratory manifestations,'flu virus identified | Respiratory | Read |
| Hyu05 | [X]Influenza+other manifestations,influenza virus identified | Respiratory | Read |
| Hyu06 | [X]Influenza+oth respiratory manifestatns,virus not identifd | Respiratory | Read |
| Hyu07 | [X]Influenza+other manifestations, virus not identified | Respiratory | Read |
| R041. | [D]Throat pain | Respiratory | Read |
| R1532 | [D]Positive culture findings in throat | Respiratory | Read |
| J43. | Emphysema | Respiratory | ICD-10 |
| J430 | MacLeod's syndrome | Respiratory | ICD-10 |
| J431 | Panlobular emphysema | Respiratory | ICD-10 |
| J432 | Centrilobular emphysema | Respiratory | ICD-10 |
| J438 | Other emphysema | Respiratory | ICD-10 |
| J439 | Emphysema, unspecified | Respiratory | ICD-10 |
| J44. | Other chronic obstructive pulmonary disease | Respiratory | ICD-10 |
| J440 | Chronic obstructive pulmonary disease with acute lower respiratory infection | Respiratory | ICD-10 |
| J441 | Chronic obstructive pulmonary disease with acute exacerbation, unspecified | Respiratory | ICD-10 |
| J448 | Other specified chronic obstructive pulmonary disease | Respiratory | ICD-10 |
| J449 | Chronic obstructive pulmonary disease, unspecified | Respiratory | ICD-10 |
| J45. | Asthma | Respiratory | ICD-10 |
| J450 | Predominantly allergic asthma | Respiratory | ICD-10 |
| J451 | Nonallergic asthma | Respiratory | ICD-10 |
| J458 | Mixed asthma | Respiratory | ICD-10 |
| J459 | Asthma, unspecified | Respiratory | ICD-10 |
| J46X | Status asthmaticus | Respiratory | ICD-10 |
| J40. | Bronchitis, not specified as acute or chronic | Respiratory | ICD-10 |
| J41. | Simple and mucopurulent chronic bronchitis | Respiratory | ICD-10 |
| J42. | Unspecified chronic bronchitis | Respiratory | ICD-10 |
| J43. | Emphysema | Respiratory | ICD-10 |
| J44. | Other chronic obstructive pulmonary disease | Respiratory | ICD-10 |
| 8795. | Asthma control step 2 | Respiratory | Read |
| H3300 | Extrinsic asthma without status asthmaticus | Respiratory | Read |
| H331. | Late onset asthma | Respiratory | Read |
| 679J. | Health education - asthma | Respiratory | Read |
| H334. | Brittle asthma | Respiratory | Read |
| 663d. | Emergency asthma admission since last appointment | Respiratory | Read |
| 9OJ5. | Asthma monitor 2nd letter | Respiratory | Read |
| 8B3j. | Asthma medication review | Respiratory | Read |
| H33z1 | Asthma attack | Respiratory | Read |
| 1782. | Asthma trigger - tobacco smoke | Respiratory | Read |
| 663t. | Asthma causes daytime symptoms 1 to 2 times per month | Respiratory | Read |
| 66YQ. | Asthma monitoring by nurse | Respiratory | Read |
| 388t. | Royal College of Physicians asthma assessment | Respiratory | Read |
| 663Q. | Asthma not limiting activities | Respiratory | Read |
| 66YC. | Absent from work or school due to asthma | Respiratory | Read |
| 663V2 | Moderate asthma | Respiratory | Read |
| 66YK. | Asthma follow-up | Respiratory | Read |
| H33.. | Bronchial asthma | Respiratory | Read |
| H3301 | Extrinsic asthma with asthma attack | Respiratory | Read |
| 663V. | Asthma severity | Respiratory | Read |
| H33zz | Asthma NOS | Respiratory | Read |
| 663q. | Asthma daytime symptoms | Respiratory | Read |
| H331. | Intrinsic asthma | Respiratory | Read |
| 9OJ2. | Refuses asthma monitoring | Respiratory | Read |
| 663.. | Asthma sometimes restricts exercise | Respiratory | Read |
| H33z1 | Asthma attack NOS | Respiratory | Read |
| 1785. | Asthma trigger - damp | Respiratory | Read |
| 663y. | Number of asthma exacerbations in past year | Respiratory | Read |
| 663N. | Asthma disturbing sleep | Respiratory | Read |
| 663P1 | Asthma limits activities 1 to 2 times per week | Respiratory | Read |
| 663N2 | Asthma disturbs sleep frequently | Respiratory | Read |
| 663j. | Asthma - currently active | Respiratory | Read |
| 9OJB. | Asthma monitorng invit SMS (short message servce) txt messge | Respiratory | Read |
| 8797. | Asthma control step 4 | Respiratory | Read |
| H3311 | Intrinsic asthma with asthma attack | Respiratory | Read |
| 1780. | Aspirin induced asthma | Respiratory | Read |
| 663W. | Asthma prophylactic medication used | Respiratory | Read |
| 663.. | Asthma monitoring | Respiratory | Read |
| H332. | Mixed asthma | Respiratory | Read |
| 679J1 | Health education - structured asthma discussion | Respiratory | Read |
| H33z. | Asthma unspecified | Respiratory | Read |
| 1788. | Asthma trigger - cold air | Respiratory | Read |
| 9OJ3. | Asthma monitor offer default | Respiratory | Read |
| 663r. | Asthma causes night symptoms 1 to 2 times per month | Respiratory | Read |
| 661N1 | Asthma self-management plan review | Respiratory | Read |
| 66Y9. | Step up change in asthma management plan | Respiratory | Read |
| 663O. | Asthma not disturbing sleep | Respiratory | Read |
| 66Yz0 | Asthma management plan declined | Respiratory | Read |
| 66YA. | Step down change in asthma management plan | Respiratory | Read |
| 9NNX. | Under care of asthma specialist nurse | Respiratory | Read |
| 663V1 | Mild asthma | Respiratory | Read |
| H3300 | Hay fever with asthma | Respiratory | Read |
| H3311 | Intrinsic asthma with status asthmaticus | Respiratory | Read |
| 66Yq. | Asthma causes night time symptoms 1 to 2 times per week | Respiratory | Read |
| 8794. | Asthma control step 1 | Respiratory | Read |
| H33z2 | Late-onset asthma | Respiratory | Read |
| 679J2 | Health education - structured patient focused asthma discuss | Respiratory | Read |
| 663w. | Asthma limits walking up hills or stairs | Respiratory | Read |
| H33z. | Hyperreactive airways disease | Respiratory | Read |
| 9OJ8. | Asthma monitor phone invite | Respiratory | Read |
| 1783. | Asthma trigger - warm air | Respiratory | Read |
| 663P0 | Asthma limits activities 1 to 2 times per month | Respiratory | Read |
| 663N1 | Asthma disturbs sleep weekly | Respiratory | Read |
| H331z | Intrinsic asthma NOS | Respiratory | Read |
| 178A. | Asthma trigger - airborne dust | Respiratory | Read |
| 663U. | Asthma management plan given | Respiratory | Read |
| 9OJA. | Asthma monitored | Respiratory | Read |
| 66YJ. | Asthma annual review | Respiratory | Read |
| 178.. | Asthma trigger | Respiratory | Read |
| 8791. | Further asthma - drug prevent. | Respiratory | Read |
| 663p. | Asthma treatment compliance unsatisfactory | Respiratory | Read |
| 9OJ1. | Attends asthma monitoring | Respiratory | Read |
| 38DT. | Asthma control questionnaire | Respiratory | Read |
| H330. | Extrinsic (atopic) asthma | Respiratory | Read |
| 679J0 | Health education - asthma self management | Respiratory | Read |
| 9OJ.. | Asthma clinic administration | Respiratory | Read |
| 1786. | Asthma trigger - animals | Respiratory | Read |
| 663x. | Asthma limits walking on the flat | Respiratory | Read |
| 388t0 | Royal College Physician asthma assessment 3 question score | Respiratory | Read |
| 66Yp. | Asthma review using Roy Colleg of Physicians three questions | Respiratory | Read |
| 8H2P. | Emergency admission, asthma | Respiratory | Read |
| 663V0 | Occasional asthma | Respiratory | Read |
| 8796. | Asthma control step 3 | Respiratory | Read |
| 663m. | Asthma accident and emergency attendance since last visit | Respiratory | Read |
| 66Ys. | Asthma never causes night symptoms | Respiratory | Read |
| 9OJZ. | Asthma monitoring admin.NOS | Respiratory | Read |
| H3310 | Intrinsic asthma without status asthmaticus | Respiratory | Read |
| 9OJ.. | Asthma monitoring admin. | Respiratory | Read |
| H335. | Chronic asthma with fixed airflow obstruction | Respiratory | Read |
| 38DL. | Asthma control test | Respiratory | Read |
| 663e. | Asthma restricts exercise | Respiratory | Read |
| 9OJ6. | Asthma monitor 3rd letter | Respiratory | Read |
| 663u. | Asthma causes daytime symptoms 1 to 2 times per week | Respiratory | Read |
| 66YP. | Asthma night-time symptoms | Respiratory | Read |
| 1781. | Asthma trigger - pollen | Respiratory | Read |
| 1789. | Asthma trigger - respiratory infection | Respiratory | Read |
| 663N0 | Asthma causing night waking | Respiratory | Read |
| 173A. | Exercise induced asthma | Respiratory | Read |
| 1O2.. | Asthma confirmed | Respiratory | Read |
| 38DV. | Mini asthma quality of life questionnaire | Respiratory | Read |
| 663n. | Asthma treatment compliance satisfactory | Respiratory | Read |
| H33.. | Asthma | Respiratory | Read |
| H3301 | Extrinsic asthma with status asthmaticus | Respiratory | Read |
| 66Yr. | Asthma causes symptoms most nights | Respiratory | Read |
| H33zz | Allergic bronchitis NEC | Respiratory | Read |
| H33zz | Allergic asthma NEC | Respiratory | Read |
| 66Y5. | Change in asthma management plan | Respiratory | Read |
| H33zz | Exercise induced asthma | Respiratory | Read |
| 663f. | Asthma never restricts exercise | Respiratory | Read |
| 38QM. | Childhood Asthma Control Test | Respiratory | Read |
| 663v. | Asthma causes daytime symptoms most days | Respiratory | Read |
| H33z0 | Status asthmaticus NOS | Respiratory | Read |
| 1784. | Asthma trigger - emotion | Respiratory | Read |
| 9OJ7. | Asthma monitor verbal invite | Respiratory | Read |
| H47y0 | Detergent asthma | Respiratory | Read |
| H330z | Extrinsic asthma NOS | Respiratory | Read |
| 663O0 | Asthma never disturbs sleep | Respiratory | Read |
| 66YE. | Asthma monitoring due | Respiratory | Read |
| 66Yu. | Number days absent from school due to asthma in past 6 month | Respiratory | Read |
| 178B. | Asthma trigger - exercise | Respiratory | Read |
| 9OJA. | Asthma monitoring check done | Respiratory | Read |
| H35y7 | Wood asthma | Respiratory | Read |
| 8798. | Asthma control step 5 | Respiratory | Read |
| H330. | Pollen asthma | Respiratory | Read |
| H330. | Hay fever with asthma | Respiratory | Read |
| 66YZ. | Does not have asthma management plan | Respiratory | Read |
| H330. | Childhood asthma | Respiratory | Read |
| H330. | Allergic asthma | Respiratory | Read |
| 66300 | Asthma severely restricts exercise | Respiratory | Read |
| H333. | Acute exacerbation of asthma | Respiratory | Read |
| 8CR0. | Asthma clinical management plan | Respiratory | Read |
| H33z0 | Severe asthma attack | Respiratory | Read |
| 1787. | Asthma trigger - seasonal | Respiratory | Read |
| 9OJ4. | Asthma monitor 1st letter | Respiratory | Read |
| H3120 | Chronic asthmatic bronchitis | Respiratory | Read |
| 663s. | Asthma never causes daytime symptoms | Respiratory | Read |
| 66YR. | Asthma monitoring by doctor | Respiratory | Read |
| 663P. | Asthma limiting activities | Respiratory | Read |
| 661M1 | Asthma self-management plan agreed | Respiratory | Read |
| 663P2 | Asthma limits activities most days | Respiratory | Read |
| 663V3 | Severe asthma | Respiratory | Read |
| 8CMA0 | Patient has a written asthma personal action plan | Respiratory | Read |
| 13O.. | Sickness/invalidity benefit | SICK | Read |
| 13O1. | Sickness benefit | SICK | Read |
| 13O2. | Statutory sick pay | SICK | Read |
| 9C8.. | LOC1/2/3- notific. of sickness | SICK | Read |
| 9C83. | LOC 3-sickness payment record | SICK | Read |
| 9DK.. | Sick note generated from secondary care done by practice | SICK | Read |
| 9K8.. | Sickness certificate | SICK | Read |
