# Supplementary figures and images for: Healthcare use attributable to COVID-19: a propensity-matched national electronic health records cohort study of 249,390 people in Wales, UK
Source: BMC Med. 2023 Jul 19;21:259. doi: 10.1186/s12916-023-02897-5 (PMC10354936; doi:10.1186/s12916-023-02897-5)

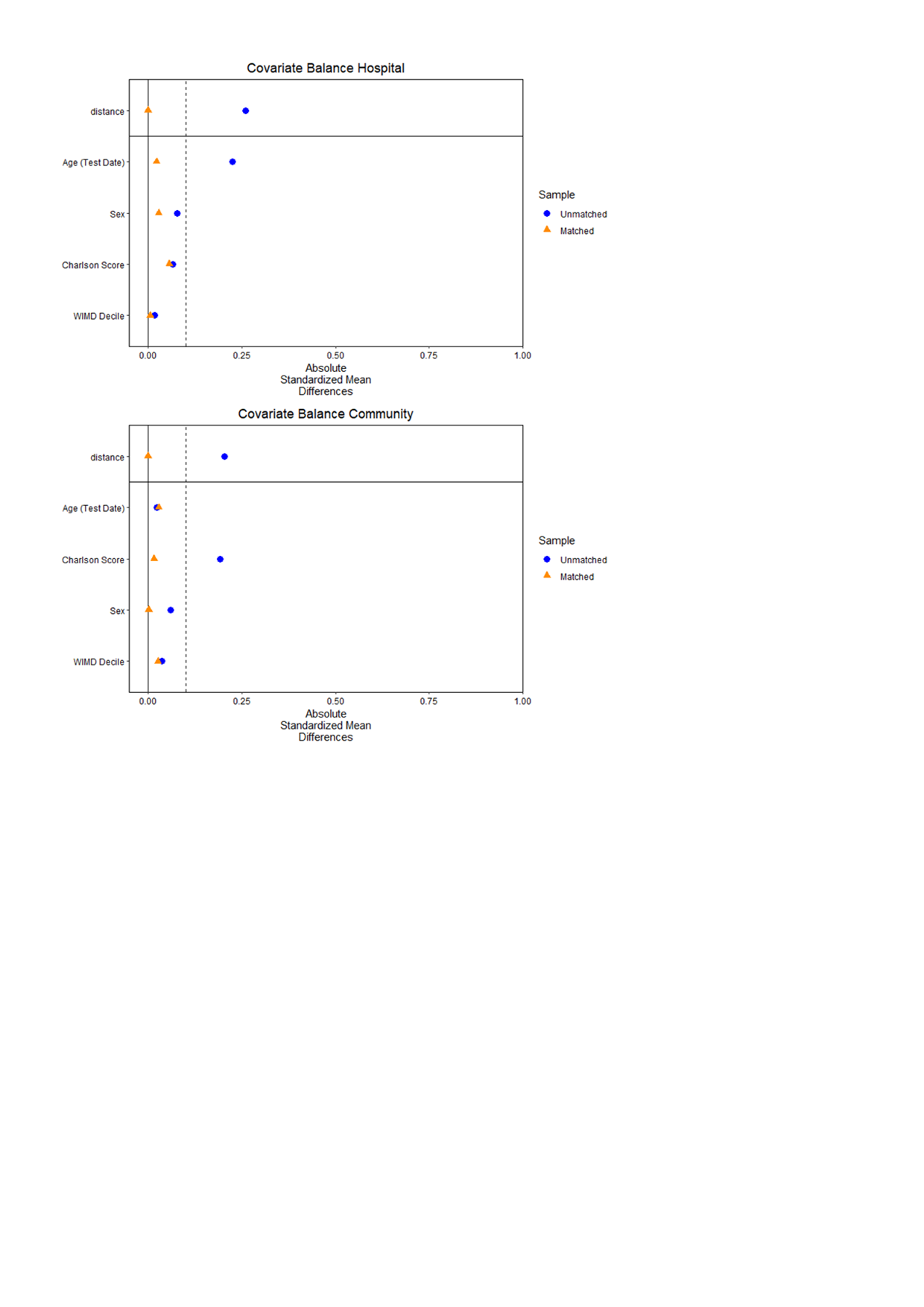

Supplement: Supplementary file 4 — Additional file 4: Fig S1. Shows ‘Love Plots’ for the main covariates before and after the propensity matching had taken place for community and hospital tested individuals. WIMD – Welsh Index of Multiple Deprivation. [file 12916_2023_2897_MOESM4_ESM.tif]

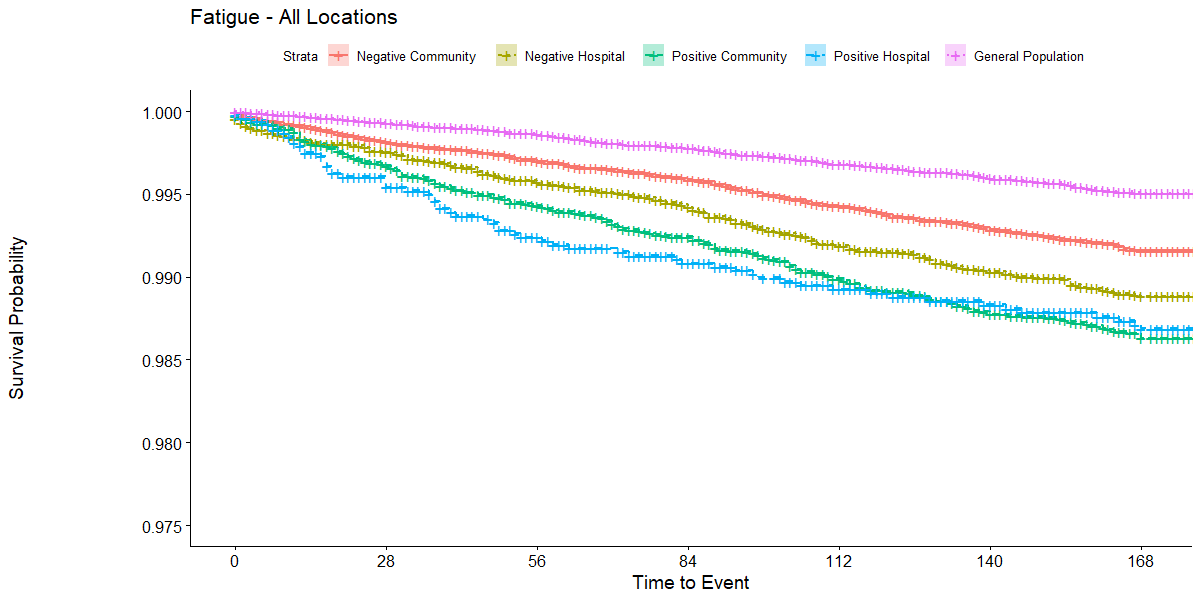

Supplement: Supplementary file 6 — Additional file 6: Fig S2. Survival for the full 6-month follow-up for the fatigue outcome. “General population” in the figure refers to the never tested population. [file 12916_2023_2897_MOESM6_ESM.png]

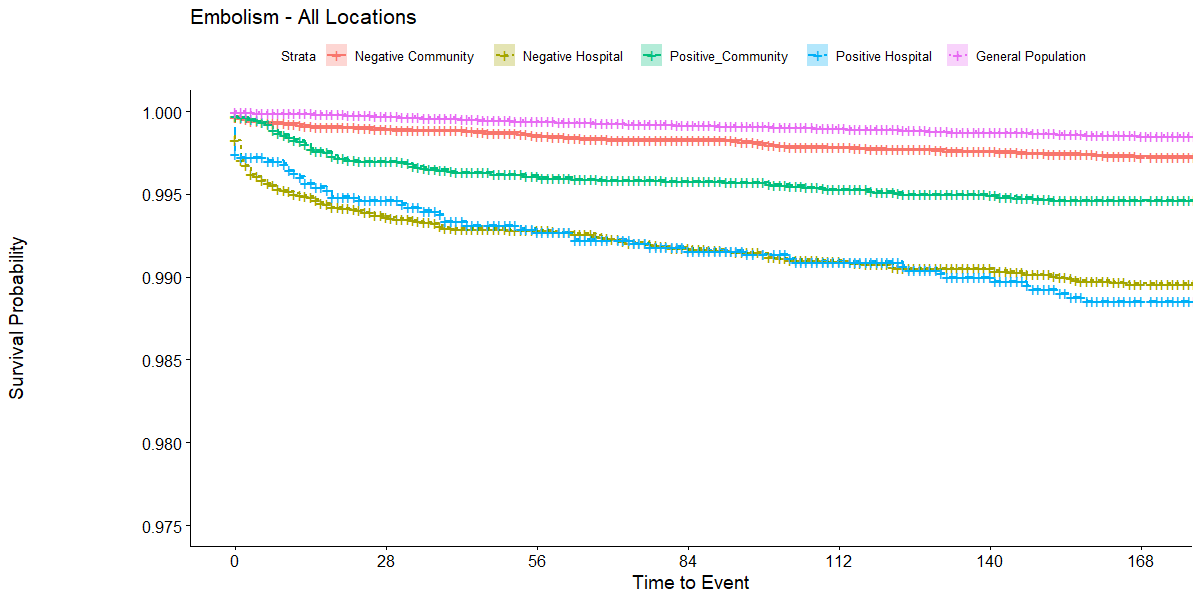

Supplement: Supplementary file 7 — Additional file 7: Fig S3. Survival for the full 6-month follow-up for the embolism outcome. “General population” in the figure refers to the never tested population. [file 12916_2023_2897_MOESM7_ESM.png]
