# Supplementary material for: Healthcare use attributable to COVID-19: a propensity-matched national electronic health records cohort study of 249,390 people in Wales, UK
Source: BMC Med. 2023 Jul 19;21:259. doi: 10.1186/s12916-023-02897-5 (PMC10354936; doi:10.1186/s12916-023-02897-5)
Supplement: Supplementary file 8 — Additional file 8: Table S5. Underlying life table data for death outcome only. [file 12916_2023_2897_MOESM8_ESM.docx]

| Deaths/100,000 | | | | | | |
| --- | --- | --- | --- | --- | --- | --- |
| Time (Weeks) | 1-4 | 5-8 | 9-12 | 13-16 | 17-20 | 21-24 |
| Negative | 980.36 | 557.53 | 344.63 | 294.23 | 250.13 | 199.08 |
| Positive | 3019.77 | 552.76 | 303.06 | 180.42 | 156.34 | 106.11 |
| Untested | 128.23 | 140.55 | 127.75 | 107.39 | 91.89 | 77.29 |
